# Investigation of a Limited but Explosive COVID-19 Outbreak in a German Secondary School

**DOI:** 10.3390/v14010087

**Published:** 2022-01-04

**Authors:** Sigrid Baumgarte, Felix Hartkopf, Martin Hölzer, Max von Kleist, Sabine Neitz, Martin Kriegel, Kirsten Bollongino

**Affiliations:** 1 Infection Protection, Local Health Authority Hamburg-Nord, 20249 Hamburg, Germany; sabine.neitz@hamburg-nord.hamburg.de (S.N.); kirsten.bollongino@hamburg-nord.hamburg.de (K.B.); 2 Methodology and Research Infrastructure, Genome Sequencing and Genomic Epidemiology, Robert Koch Institute, 13353 Berlin, Germany; HartkopfF@rki.de; 3 Methodology and Research Infrastructure, Bioinformatics, Robert Koch Institute, 13353 Berlin, Germany; HoelzerM@rki.de; 4 Systems Medicine of Infectious Disease, Robert Koch Institute, 13353 Berlin, Germany; KleistM@rki.de; 5 Herrmann Rietschel-Institute, Technical University of Berlin, 10587 Berlin, Germany; m.kriegel@tu-berlin.de

**Keywords:** SARS-CoV-2, school outbreak, superspreading event, aerosols, non-pharmaceutical interventions

## Abstract

The role of schools as a source of infection and driver in the coronavirus-pandemic has been controversial and is still not completely clarified. To prevent harm and disadvantages for children and adolescents, but also adults, detailed data on school outbreaks is needed, especially when talking about open schools employing evidence-based safety concepts. Here, we investigated the first significant COVID-19 school outbreak in Hamburg, Germany, after the re-opening of schools in 2020. Using clinical, laboratory, and contact data and spatial measures for epidemiological and environmental studies combined with whole-genome sequencing (WGS) analysis, we examined the causes and the course of the secondary school outbreak. The potential index case was identified by epidemiological tracking and the lessons in classrooms with presumably high virus spreading rates and further infection chains in the setting. Sequence analysis of samples detected one sample of a different virus lineage and 25 virus genomes with almost identical sequences, of which 21 showed 100% similarity. Most infections occurred in connection with two lesson units of the primary case. Likely, 31 students (12–14 years old), two staff members, and three family members were infected in the school or the typical household. Sequence analysis revealed an outbreak cluster with a single source that was epidemiologically identified as a member of the educational staff. In lesson units, two superspreading events of varying degrees with airborne transmission took place. These were influenced by several parameters including the exposure times, the use of respiratory masks while speaking and spatial or structural conditions at that time.

## 1. Introduction

Subsequently to the declaration of the coronavirus disease 2019 (COVID-19) as a pandemic by the World Health Organization (WHO) in March 2020, in many countries, schools were closed as a part of lockdown concepts. To prevent irreversible harm to children and adolescents, workable solutions and safe models for open schools that reduce the risk of virus transmission in and outside the school were needed. Evidence-based hygiene concepts in schools and effective non-pharmaceutical interventions (NPI) became even more critical with the increasing worldwide spread of highly infectious virus variants of concern (VOC) of the severe acute respiratory syndrome coronavirus type 2 (SARS-CoV-2). Therefore, there is a need for public health research data from school outbreak investigations, including information about the detailed course, the various influencing factors, and risks for virus transmissions in schools.

### Background Information

On 16 March 2020, a shutdown was put into effect in Germany, which included the closure of schools and kindergartens, assuming that these settings may play a leading role in the transmission dynamics of SARS-CoV-2 analogous to the influenza virus. In the following months, the role of children and schools in the pandemic was widely discussed, with conflicting opinions expressed. Various studies revealed that children of all age groups are susceptible to SARS-CoV-2 infections but develop milder symptoms than adults, with severe courses and fatal outcomes being rare [1,2,3]. Initially, it was considered unlikely that children may be the main drivers in the pandemic [4].

An early investigation of outbreaks in school settings found no references for transmission among children in Ireland and concluded that schools might play a subordinate role in spreading SARS-CoV-2 [5]. In contrast, the description of a significant school outbreak in Israel showed numerous COVID-19 infections in May 2020, when favorable circumstances for a high SARS-CoV-2 transmission rate accumulated [6]. In Australia, where schools stayed open during the first wave, students and teachers did not contribute significantly to virus transmissions in the Australian population after attending educational institutions [7].

The dynamics of the first and the following waves of the COVID-19 pandemic differ in several aspects. Hence, the assessment of the role of children changed to some extent. At the end of 2020, some studies suspected children to be a potential source of contagion in this pandemic [2] and play an important role in virus transmission in India [8]. In high-income countries with older populations, like Germany, research on outbreaks in educational settings did not find schools to be pandemic drivers [9]. Instead, they showed a significant correlation between school infection rates and overall COVID-19 incidence, as did a comprehensive study from England [10]. In 2021 the increasing dominance of the Delta variant changed the situation worldwide. In contrast to summer 2020 in Australia a five-fold higher rate of transmission of this VOC was observed in outbreaks in schools and preschool institutions (ECEC services). The children only showed none or mild symptoms of COVID-19 and 2% of them were hospitalized [11]. An investigation of a COVID-19 primary school outbreak in Marin County, USA, concluded that the Delta variant is highly infectious [12]. 

Many disadvantages of school closures were described, especially for children and adolescents’ physical and mental health [13]. In the meantime, public health organizations, such as the WHO, CDC, and ECDC, agreed that closing schools should be the last option due to their fundamental importance for children and society. While vaccination efforts are ongoing and the impact of emerging VOCs on schools are unclear, striking school outbreaks need to be analyzed for detailed risk factors to develop and validate safe approaches for open schools. This should include the data of environmental conditions and the quality of ventilation, as well as the use of masks and the behavior of the people involved. These aspects play an important role in the dynamic of COVID-19 outbreaks due to the frequent aerosol transmission, often at super spreading events [14,15,16,17,18,19,20]. 

After the summer vacations in 2020, when less than 15 COVID-19 incidences were reported in Hamburg (Germany), and the risk of infection for children was assumed to be low, schools were re-opened with new hygiene concepts following a masterplan developed by the city [21]. Some of the plan’s cornerstones were separating grade levels with the suspension of normal rules for physical distance within these cohorts, mandatory hand hygiene, and general mask requirements outside the classrooms. During the second wave of the pandemic, a COVID-19 school outbreak in Germany led to an extended investigation by the responsible local health department. This paper describes the retrospective analysis of epidemiological data, whole-genome sequencing, and the complex influences on virus transmission in the school setting. This includes a timeline of the symptom onset of cases combined with sequence data and containment measures from the health department and spatial and ventilation conditions in the school.

## 2. Materials and Methods

### 2.1. Measures of the Local Health Authority

**Figure 1:**
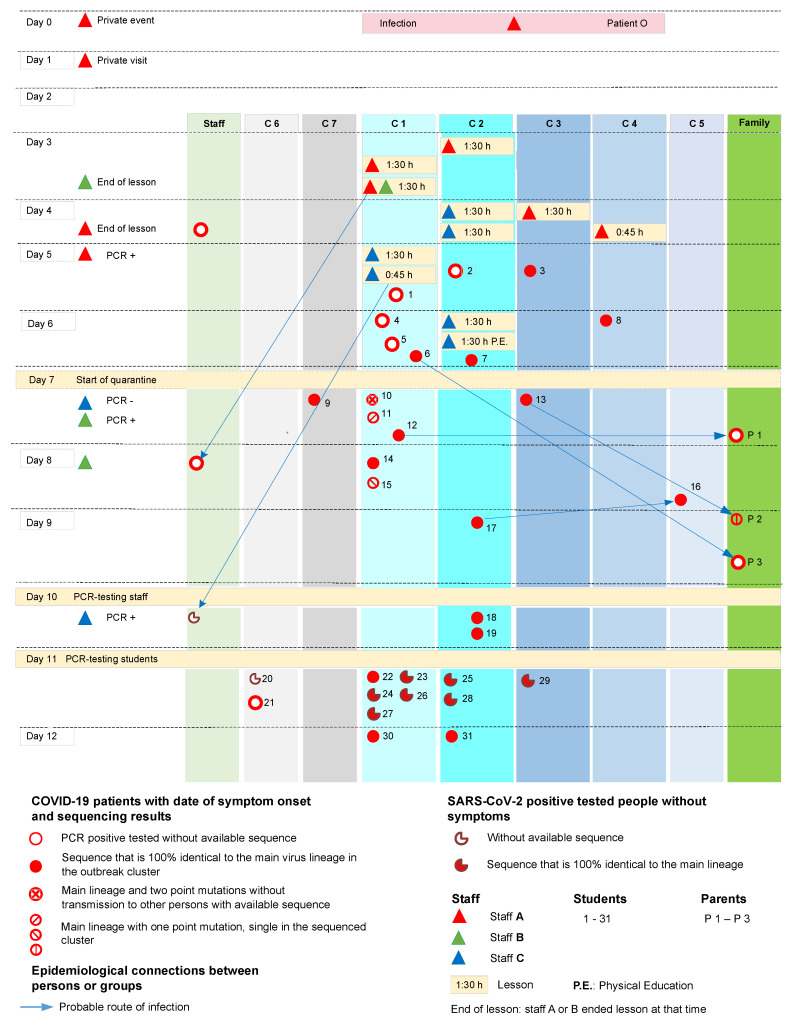
Chronological overview over the outbreak course. The chronological assumption of relevant data for the course of the outbreak is shown. This includes results of epidemiological and sequence analysis as well as nonpharmaceutical interventions (NPI). The date of their first positive PCR-test was used to match asymptomatic individuals on a timeline instead of symptom onset of the symptomatic COVID-19 patients. Numbers of students and parents follow the chronology of symptom onset and positive results of the PCR tests.

The first case of an educational staff member who tested positive for SARS-CoV-2 in the school was reported to the Health Department in September 2020 (day 6 in Figure 1). On the same day, a further report of a student who tested positive, followed and the suspicion of an outbreak was communicated to the school management, other health departments, and the health authorities of the city. An initial quarantine was implemented on day 7. The measures to contain the SARS-CoV-2 spreading and the data collected were based on regional public health service guidelines. These included PCR tests, the use of a questionnaire-based telephone inquiry about patient data, and track and trace of close contacts as quarantine measures.

The data has been entered into the national digital recording system (“Octoware”) and recorded manually in an Excel table. This table included personal data (name, date of birth, age, gender, student, or staff), dates of symptom onset, dates of the last day in school, dates and results of PCR tests, symptoms, close contacts, and family members. Two mass PCR tests for all affected grade level of students and all staff members of the affected building were organized via a central distribution system. They took place on day 7 and 11 for educational staff and students. Oropharyngeal swab samples were taken to test for SARS-CoV-2 and analyzed by RT-qPCR in accredited laboratories.

On day 11, the health department commissioned a whole-genome sequencing (WGS) for the outbreak analysis and asked 15 laboratories of the network “FAST-Track”, carrying out the primary PCR-diagnostics, to send the anonymously coded positive samples to the sequencing laboratory. Following the school concept of separation of cohorts, a quarantine for all affected grades and all staff members with potential risk was ordered, starting on day 7. This quarantine was revoked on day 13 for symptom-free people with a negative PCR test result and went on for 14 days for SARS-CoV-2 positive tested people. Infected persons were contacted daily by telephone, and close contact persons were contacted every 2 to 3 days to ask about their condition and symptoms.

In the management of this outbreak, COVID-19 cases were defined as those that tested positive for SARS-CoV-2 by PCR test in accordance with a part of the case definition of the Robert Koch Institute, which is used as the basis for the official infection report [22]. Cases with COVID-19 symptoms and negative test results were not observed. People with COVID-19-like symptoms were generally excluded from attending school. All family members in the same household of infected students and teachers were classified as close contacts with a high risk of infection and were quarantined for 14 days. The families were instructed to isolate the infectious person in their room and separate activities as far as possible.

The last onset of symptoms in patients with a secure connection to the outbreak was reported on day 12. One family member with an additional possibility of infection at his workplace had COVID-19 symptoms on day 24 and was excluded from the outbreak investigation. The outbreak was contained, and all measures were lifted 35 days after the onset of the outbreak.

### 2.2. Retrospective Follow-Up and Detailed Examination of the People Involved, Background, and Environmental Data in School at That Time

The whole outbreak was analyzed retrospectively using and validating the data of the health department and the school management. The school director, all SARS-CoV-2 positive tested and associated staff members, some of the students involved (especially those who were tested positive but were not present in lessons with the primary case on day 3 and 4), and some family members were interviewed by phone after the consensus of the concerned or responsible persons.

Spatial conditions were analyzed with building plans and local data. The main classrooms, washing rooms, and corridors were inspected and professionally measured. This data was used for the creation of seating plans and the calculation of room space volumes.

### 2.3. Statistics: Infection Risk during the Lessons of Staff A on Day 3 and 4

Statistical analysis was performed after grouping lessons of day 3 and day 4. We used the Kaplan-Meier estimator provided in MatSurv [23] to calculate the probability of being non-infected on day 3 and day 4 and the hazard ratio (HR) between those days. Actual infection times were assumed to occur uniformly over the class duration, and statistical differences between the infection rates were computed using a log-rank test implemented in MatSurv.

### 2.4. Laboratory Diagnostics, Whole-Genome Sequencing, and Bioinformatics Analysis

#### 2.4.1. Sample Collection and Primary PCR-Diagnostic

Oropharyngeal swab samples were taken by employees of the health department in large series during two days of mass testing in the school. SARS-CoV-2 RT-qPCR diagnostics were carried out by 15 accredited laboratories in Hamburg, which used different commercial PCR kits. Additional samples from two employees and two parents were taken privately and tested positive in other laboratories.

Three laboratories of the FAST-Track network generated positive test results. They mainly used the Cobas Roche system but also Anchor Diagnostic, Biopharm, or Altona Diagnostic PCR-Kits.

#### 2.4.2. Second SARS-CoV-2 RT-PCR

28 positive tested samples from three primary laboratories of the network were sent for independent SARS-CoV-2 RT-qPCR confirmation and virus whole genome sequencing to the Institute for Medical Microbiology, Virology and Hygiene, University Medical Center Hamburg-Eppendorf (UKE). For RT-qPCR, samples were mixed at a ratio of 1:1 with Roche PCR Media kit buffer (Roche, Basel, Switzerland). SARS-CoV-2 qPCR was performed according to authors description [24,25].

#### 2.4.3. SARS-CoV-2 Amplicon Sequencing and Bioinformatic Analysis

After excluding two samples, that tested negative, 26 positive samples (25 from students and one from a family member) were sequenced as described earlier using an amplicon-based approach, the ARTIC V3 primer scheme, and Illumina short-read sequencing [26]. Resulting data was compared to other regional, national, and international SARS-CoV-2 sequences in data collections and international sequence databases (collection of UKE und Heinrich Pette Institute, NCBI, GISAID). In 2021, raw sequence data was provided to the Robert Koch Institute (RKI) in Berlin for genome reconstruction, investigations for connections before and after the outbreak using phylogenetic analysis and determining the lineage of the specimens involved.

#### 2.4.4. Genome Reconstruction

Amplicon sequencing data was analyzed using CovPipe v3.0.5 [27]. CovPipe provides a fully automated and reproducible workflow for reconstructing genome sequences from amplicon-based Illumina sequencing data given a reference sequence. In short, reads were quality- and adapter-trimmed and human host contamination were removed utilizing a custom database of SARS-CoV-2 genomes and the human reference genome. Then processed reads were mapped to the Wuhan reference genome (NC_045512.2), and ARTIC V3 primers were clipped from the resulting BAM files. Finally, variants were identified with freebayes v1.3.2 [28] and filtered using the default parameters of CovPipe. The resulting consensus sequences were quality-checked by read coverage and the number of ambiguous N bases and SARS-CoV-2 lineages assigned using Pangolin v3.1.5 [29] and pangoLEARN 2021-06-15. To further reduce potential sequencing or reconstruction errors (e.g., of unique mutations only found in few samples), we 1. additionally searched our national collection of German sequences and GISAID for convergent occurrence of the mutations in other lineages and 2. performed manual examinations of unique sites via a genome browser, which revealed high read support even for unique sites. 

#### 2.4.5. Phylogeny

The assembled sequences were used to calculate a phylogeny to show the divergence of the outbreak samples and other available international sequences. The tree was calculated using a custom pipeline with various tools, including Augur [30] (utilizing IQ-TREE, TreeTime, MAFFT), Pangolin [29], and sequences from GISAID [31], Charité Berlin, the German Electronic Sequencing Data Hub (DESH), and the Integrated Molecular Surveillance program at the RKI.

Before processing, 3311 sequences were identified as closely related to the outbreak using the GISAID AudacityInstant functionality and were added to the outbreak sequences. Additionally, international clade representatives were selected, and sequences from Germany were randomly selected to increase the total number of sequences to at least 25 for each German division per month if enough sequences were available. This subsampling yielded 9471 sequences after filtering with an N-threshold of 5% and removing samples with missing metadata. Based on their importance due to epidemiological data, three of the outbreak samples were added back to the analysis disregarding the N-threshold, namely 13 (5.2%), 23 (9.44%), and 11 (16.75%). Wuhan-Hu-1 (MN908947.1) was used as a reference for all subsequent analyses. From the calculated tree, a subtree with all B.1.177 outbreak samples and closely related sequences was extracted using the ETE3 toolkit [32] and was partially annotated and visualized with iTOL [33].

### 2.5. Hygiene Concept in the School in 2020

In addition, the hygiene concept of the school and the city’s masterplan for re-opening schools in 2020 have been examined. At that time, the schools in Hamburg implemented a hygiene concept following a hygiene master plan of the city [21]. The most important cornerstones at that date included:

- Exclusion from school if COVID-19 symptoms occur (except for mild rhinorrhea).
- Separation of grade levels: “cohorting”.
- Abolition of the 1.5 m distance rules within the grade levels, in classrooms and other places, where it is challenging to keep a distance.
- It was mandatory to wash or disinfect hands immediately before class.
- Mask requirement in corridors, staircases, toilets, and outside of the building.
- No obligation to wear masks in the classroom, voluntary use of mouth/nose protection for students and staff.
- Recommendation to ventilate several times a day through fully opened windows via intermittent or cross ventilation, usually during breaks and only occasionally during class.

The concept was prepared during vacations and communicated at the re-opening of schools.

## 3. Results

### 3.1. General Data of the Outbreak in the School Setting

In a Secondary School building, which is part of a big school complex, where three grade levels are taught, the affected two grade levels with a total of 368 students were initially quarantined and tested for SARS-CoV-2. 33 of them and three educational staff members out of all staff in the building had positive results (Table 1). 

Two of the infected students turned out to have other sources of infection that were not connected to the outbreak. The most affected class with 15 CoV-positive tested students and two positive tested educational staff members were class C.1, followed by classes C.2, C.3, and C.4. One further student of class C.5 and three of C.6 and C.7 were also infected (Figure 1). Out of the seven family contacts who were tested positive at that time, four had other sources of infection (see Section 3.3.1). 

### 3.2. Clinical Data

The most common symptoms of the outbreak’s COVID-19 cases were rhinorrhea (71%), sore throat (42%), headache and cough (each 39.5%), fatigue (36.8%), fever, diarrhea/ nausea, and muscle pain (each 18.4%) and loss of taste or smell (13.5%). Only light or asymptomatic COVID-19 disease courses were observed during the outbreak. Complications, hospitalizations, or deaths are not known.

### 3.3. Potential Infection Chains in the Outbreak Cluster

#### 3.3.1. Exclusion of Potential Index Cases, the People Involved, and Transmission Events

**Figure 2:**
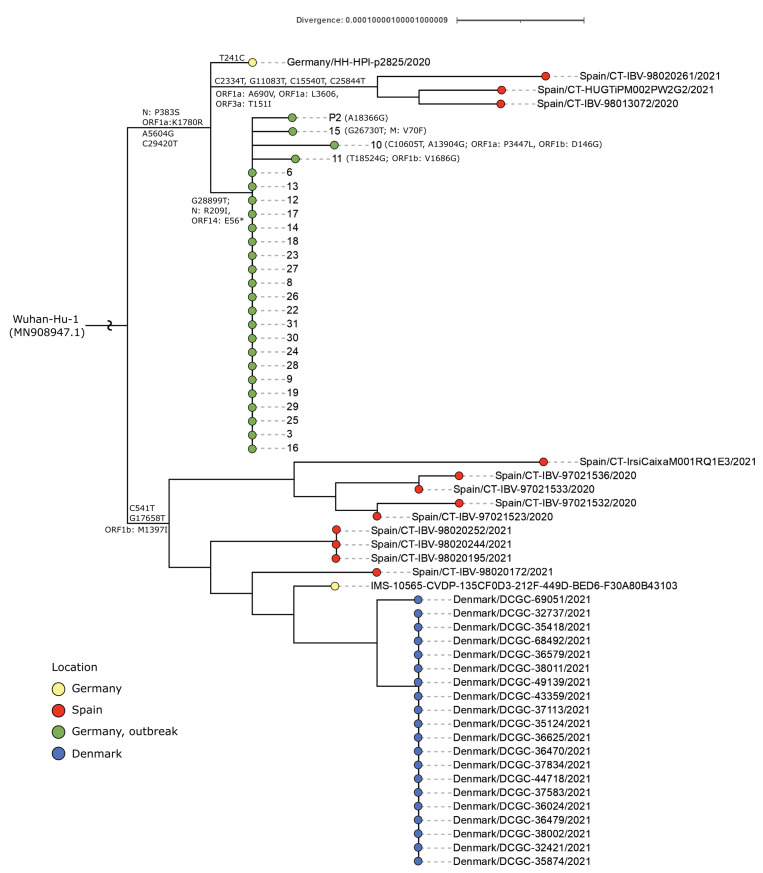
Phylogeny of the outbreak B.1.177 samples in context with international samples. Sample numbers of the green circles follow the numbers of students (1–31) and parents (P1–3) with available sequenced samples shown in Figure 1. A phylogenetic tree was calculated using preselected 9471 German and international SARS-CoV-2 sequences in a custom pipeline (see Methods). The subtree of the Pangolin lineage B.1.177 shown above was extracted and annotated. It shows six geographical clusters: two clusters with sequences originating from Spain (red), one cluster from Denmark (blue), three clusters from Hamburg, Germany. The clusters from Germany can be split into the investigated outbreak (green) and two clusters (yellow) with only one sequence.

Two students, who showed the earliest onset of COVID-19 symptoms, can be excluded as index patients. In the first case, a sample sequence analysis revealed a different lineage for the detected virus (B.1.258) and ruled out the student as part of the described outbreak. Detailed research led to a later date for the symptom onset of the second case (1) *, which initially showed other symptoms than those inherent to COVID-19. (* Numbers in round brackets are used to identify student or parent numbers in Figure 1 and sample numbers in Figure 2). 

Various opportunities for virus transmission, such as team meetings of educational staff, break times in the staff room, or parent evenings, can be excluded as spreading events since these events were not associated with later COVID-19 cases. One infected student and his family without available sequence data were excluded as part of a separate outbreak based on the time of symptom onset. An infected parent from another family was excluded because of the same reason.

#### 3.3.2. The Index Case, Superspreading Events, and Infected Educational Staff Members

An educational staff member was identified as the primary case (staff A) despite missing sequence data. His first onset of COVID-19 symptoms, the established source of his infection, and the comprehensible infection routes following his school lessons indicated staff A as the index case. His removal combined with large quarantine measures stopped the expansion of the outbreak.

Staff A tested negative in a SARS-CoV-2 PCR six days before his COVID-19 symptom onset, before starting private visits over two days (day 1 and 2). On the evening of day 0, staff A took part in a sportive leisure event, which later turned out to be a superspreading event with infections of all susceptible participants. The index case of that event was infected in another known superspreading event from this period. On day 1 and 2, staff A carried out the visits as planned. None of the contacts became infected.

On day 3, staff A gave lessons for 1.5 h in class C.2 and two 1.5 h lessons with a 30-min break in class C.1. For protection, A only used a simple face mask with one layer of cotton in direct contact with the students, but no mask at the desk or in front of the class. Most of the lesson was done by lecturing while standing in front of the class without mouth/nose protection.

On day 4, staff A noticed light rhinorrhea in the morning. Staff A went to work but changed behavior slightly by wearing the cotton mask continuously, holding a distance of more than 1.5 m consistently and speaking less. Staff A gave lessons for 1.5 h in class C.3 in room 3 and stopped the following lesson in class C.4 after 45 min because of light Flu-like symptoms.

**Figure 3:**
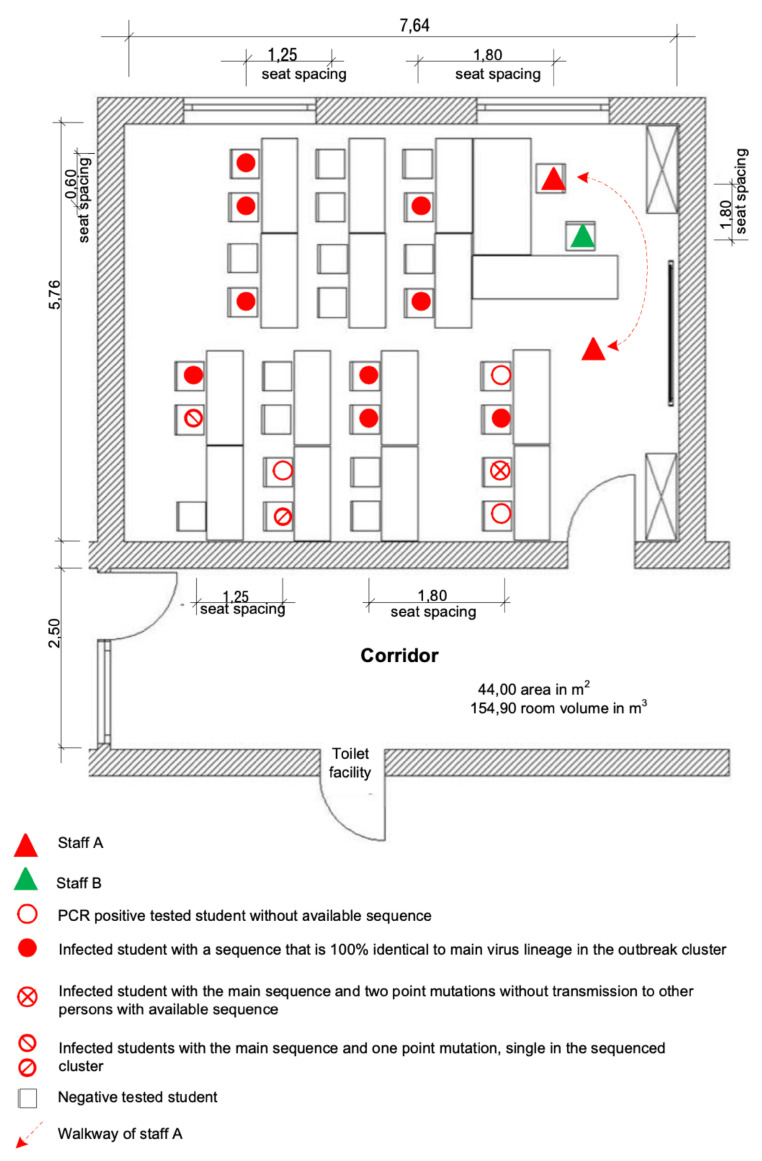
Seating plan at the time of the outbreak and SARS-CoV-2 infected persons in room 1 of class C.1.

A second staff member, B, was present at the second lesson in class C.1 on day 3. B used no mask and sat on the writing desk in front of the door, close to staff A (Figure 3). Staff B often left this place and got in touch with students, sometimes together with A. This 1.5 h of the lesson were staff B’s only close and unprotected contact with staff A and students in that week and consequently the most likely source of B’s SARS-CoV-2 infection. No other private contacts of B are known to have been SARS-CoV-2 positive at that time.

The third staff member C, who also got infected, had several junctions to staff A and the affected classes. Staff C used a barely occupied team room (44 m${}^{2}$) with A on day 3 and 4 without a mask with 3 m between them. No other colleagues, who used the same room at that time, got infected.

The most probable event of infection of staff C was a lesson of 3 units (1.5 h of lesson, 30 min break, and 45 min lesson) on day 5 in class C.1 in classroom 1. Staff member C always used a simple mask with one layer of cotton during all lessons while the children had no mouth/nose protection. On that day, probably up to 11 infected children in their incubation period of COVID-19 were in the classroom. One of them (1) got symptom onset later the same day, three of them (4, 5, 6) on the following day. Nevertheless, there was a second possibility of infection of staff C from students on day 6 during two lessons of 1.5 h in class C.2. The second lesson was physical education (P.E. in Figure 1). On that day, up to five present students of class C.2 were in their incubation period. One of them (7) got symptoms later that day. Subjectively, staff C suspected that the infection happened on day 5 due to the poor ventilation and tightness in room 1 of class C.1. This thesis is supported by the number of infected present students in this class.

#### 3.3.3. Further Possible Routes of Infection: Additional Connections and Contacts

On day 4, a minor emergency meeting occurred during the first break in room 1 of class C.1. Here, three students, two positive and one negative tested student, and two staff members, including the index case A, had gathered to help a fourth student. The two students, who tested SARS-CoV-2 positive, also took part in class the day before in room 1. Staff member D was present for 30 min, used an FFP2 mask and stayed SARS-CoV-2 negative. Staff A was present for 10 min while using a mask with one layer of cotton, keeping distance, and not talking a lot. At this event, the number of people attending was small, and some windows were open. The transmission was significantly less probable than in the previous day’s lessons because, apart from the environmental conditions, the exposure time was short, and A had already changed the behavior (distance, less speaking, constant use of mask).

Four students of two grade levels got infected, although they did not have lessons with A on day 3 or 4. Family members were likely infected at home. 

##### Class C.5

One student (16) with the main outbreak sequence spent the breaks in close contact with two friends. One of them probably got infected in class C.2 and showed the same sequence pattern.

##### Class C.6

The two students (20, 21) of class C.6, who had no available sequence data, sat across from each other at a table and had further close contact in the classroom. One of them played ball games coincidentally with students from the most infected class level during the breaks.

##### Class C.7

One student (9) who was found to have the main outbreak sequence spent his breaks playing various sporting games with other students of different grades, including those who attended staff member A’s classes.

##### Family Members

Three close family members were infected by their children in the home environment. One of them (P2) presented the cluster sequence, the other two can easily be assigned epidemiologically to their infected children (P1–P3 in Figure 1).

#### 3.3.4. Further Infections by the Attendees of the Lessons with Staff A

Only one student (8) of class C.4 who attended the lesson of staff A on day 4 got symptoms 2 days later and subsequently tested positive for SARS-CoV-2. Staff A ended that lesson after half of the time when he started to feel sick. This student was probably infected directly by staff A, but also had close contact to friends from class C.1, which might have been an alternate source of infection. 

The classes taught by staff A on day 3 and 4 stayed in school for two to three more days. It cannot be ruled out that further infections between students took place during this time. Especially in close contacts and crowded situations, as in the washing rooms and toilets, aerosol infections and without mouth/nose protection also droplet infections were possible.

### 3.4. Lineage Assignment and Phylogenetic Analysis of the Sequences

Of the 26 samples acquired and sequenced from the outbreak (Table 1), 25 sequences were assigned to the B.1.177 Pangolin lineage and showed a low diversity level. The mutation N:R209I and the deletion ORF14:E56* can be observed in all these samples and distinguish this outbreak from other national and international collected samples (Figure 2).

Only four sequences show unique mutations not found in the other sequenced outbreak samples, as displayed in Figure 1. The phylogeny reveals five additional clusters that differ in geographical information: one cluster with 20 samples in Southern Denmark, two clusters with nine or three samples in Catalonia, Spain, and two single sequences from Hamburg, Germany. Only one of these two sequences was sampled before the Hamburg outbreak on August 24, 2020 and was not uploaded to GISAID until 2021 (ID: Germany/HH-HPI-p2825/2020 in Figure 2). The other one was found in 2021 without further detailed information. Sample 32 was assigned to the Pangolin lineage B.1.258 and is not closely related to the other outbreak samples. Therefore, sample 32 is not listed in Figure 2, but similar sequences were detected in Denmark, England, and Scotland.

### 3.5. Spatial Conditions and Ventilation

The COVID-19 outbreak was limited to one old building of the entire school’s complex. All the rooms used by the COVID-19 infected people are spread over two floors in the immediate vicinity. The spatial conditions are partly cramped, while ventilation options were limited and often deficient in stairwells and toilets and classrooms at that time due to safety reasons. Narrow toilets and washrooms, separated for girls and boys, are shared by one floor.

#### 3.5.1. Classroom 1 of Class C.1

Room 1, used by class C.1 (Figure 3), shows some unique features as it only has two windows and smaller folding windows compared to other similar classrooms (Supplement Figure S1). Because of the heavy traffic in front of the classroom door, it was usually locked up and ventilation was therefore only one-sided without a draft. At the time of the outbreak in 2020, the windows were open during the breaks and mostly closed during the class, except for two small flaps at the top of the windows.

On day 3, one day before the onset of symptoms of staff A, 27 students, and staff A and B were in classroom 1 for 1.5 h without the supply of fresh air until the break of 30 min. Afterwards, a second 1.5 h lesson followed (Figure 3). Only four of six little flaps and two of six casements of the windows in the room could have been opened during the break.

Fifteen out of 27 students and both educational staff members present, of which one, staff A, turned out to be the primary case, tested SARS-CoV-2 positive after this lesson. Staff B did not have any further close contact with A or other SARS-CoV-2 positive tested people until his positive PCR test and the onset of symptoms.

A chronology of the whole outbreak course with known sequence data and interventions is shown in Figure 1.

#### 3.5.2. Room 2, 3, and 4 of Classes C.2, C.3, and C.4

**Figure 4:**
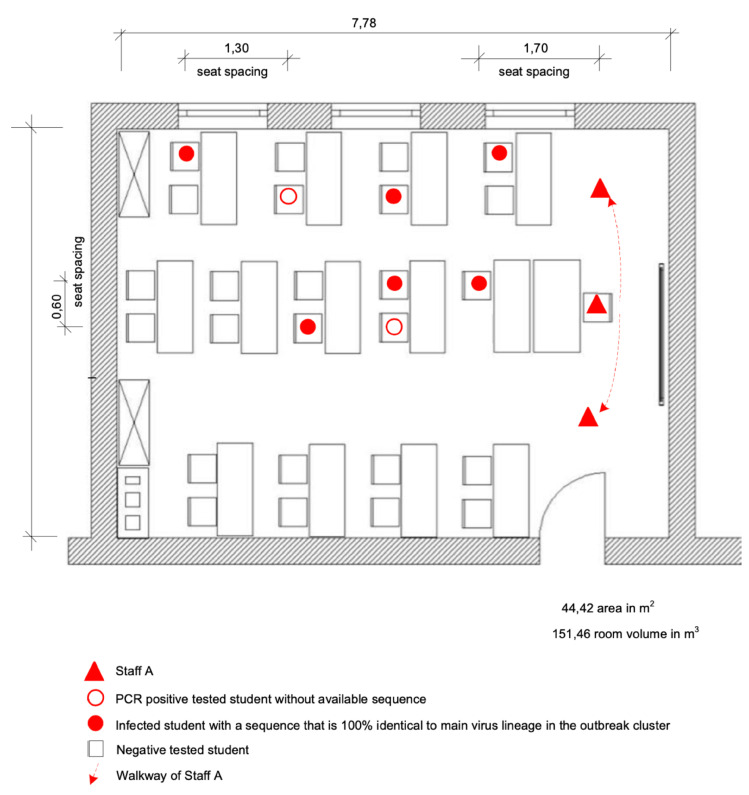
Seating plan at the time of the outbreak and SARS-CoV-2 infected persons in room 2 of class C.2.

**Figure 5:**
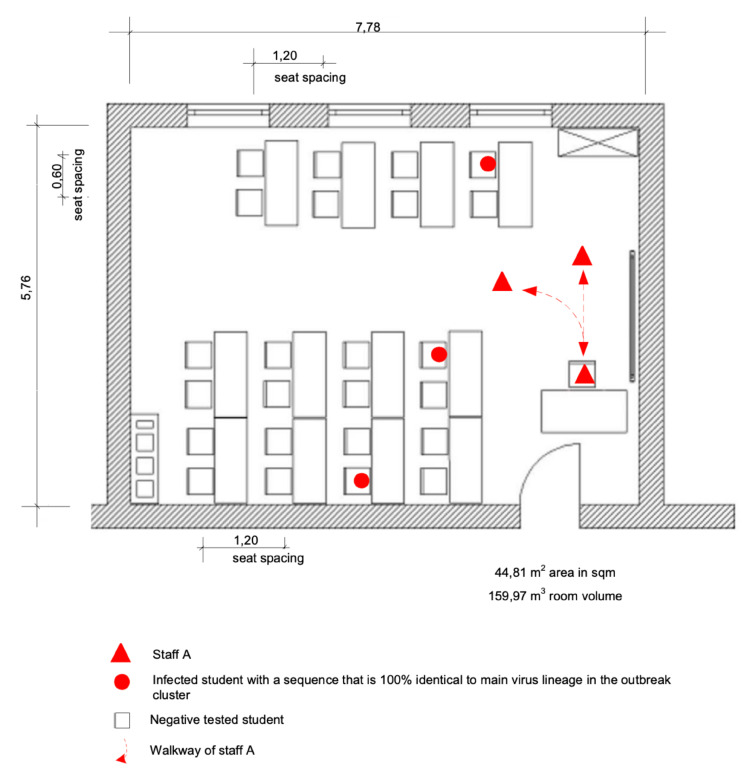
Seating plan at the time of the outbreak and SARS-CoV-2 infected persons in room 3 of class C.3.

Room 2 (Figure 4), room 3 (Figure 5), and room 4 are nearly identical in construction.

They have three big windows with three big flaps. 2/3 of the big windows and all flaps could be opened, which is significantly more than in room 1 (Supplement Figure S1). Staff A taught class C.2 on day 3 in room 2, mostly without mouth/nose protection.

The seating plan of room 4 is not shown because the room has the same design as rooms 2 and 3, and only one student got infected (Figure 1, Table 2).

On day 4, staff A taught in class C.3 in room 3 (Figure 5) and in class C.4 in room 4. That day staff A felt well and only had very light rhinorrhea in the morning. As A realized flu-like symptoms throughout the morning, the staff member ended the lesson in class C.4 directly after the first 45 min (Figure 1, end of lesson). Staff A used mouth/nose protection during the entire lesson on day 4, spoke little, and kept a considerable distance from the students. An overview of the changing conditions and influencing factors during the two days of teaching by staff A is shown in Table 2.

## 4. Discussion

Here we describe a COVID-19 school outbreak with SARS-CoV-2 transmissions starting from a pre-symptomatic educational staff to presumably 23 adolescent students and another staff member during superspreading events (SSE) in classrooms. Additionally, direct transmissions from the primary case on the following day and later infections from student-to-student, student-to-family members, and probably also student-to-staff took place. The analysis of 25 sequences from positive-tested students and one family member showed nearly similar sequences of the virus lineage B.1.177 up to one exception with another virus lineage (B.1.258) and revealed a common source of the outbreak. The collection and analysis of clinical and laboratory data, the hygienic concept at that time, epidemiological investigations of the infection chains, and the spatial and ventilation conditions in the concerning classrooms clarified the context and causes of the outbreak.

Our results show that SSEs and transmissions in all directions can happen in a school setting if different unfavorable circumstances coincide. At the same time, preventive concepts can avoid or minimize infection clusters, and every single protective measure may reduce the risk or change the dynamic of infections. According to our observations, apart from the grade of infectiousness and the behavior of the primary case, the exposure time, the protection with masks of the involved persons and, the environmental conditions determine the number of transmissions. Changeable circumstances as exposure time during lessons, consistent indoor use of masks and individual safety behavior, number of people present, space and distances between individuals in rooms, and ventilation quality are the most important factors in avoiding SARS-CoV-2 transmissions and SSEs in schools. Immediate and broad-based quarantine measures at the first suspicion of an outbreak and mass testing of all potentially affected persons effectively stopped the spreading.

Although students were suspected of playing a less important role than adult staff members in school outbreaks [10,34], we detected 31 SARS-CoV-2 positive students and only three impacted staff members in our setting. This is consistent with the European surveillance data, where educational staff and other adults in schools were not at higher risk of infection than students [35,36].

A recent investigation of an elementary school outbreak with the Delta variant of SARS-CoV-2 in the US [12] also identified an unvaccinated teacher as the index case in this outbreak. The teacher read aloud to the class without a mask and probably infected 50% of the students present in the room with most infections detected in the first two rows. The authors do not provide more detailed information about the exposure times but reported open windows during the lesson. Finally, they infer the high attack rate (AR) of 50% in the classroom from the high infection potential of the Delta variant. We observed an AR of 57% during one lesson with infected persons positioned throughout the classroom and very different AR under varying conditions even though the causative SARS-CoV-2 (B.1.177) was not classified as a VOC or VOI. It is possible that better ventilation in the primary school classroom resulted in comparable AR in both outbreaks, although higher infectiousness of the Delta variant is assumed. NPI and masking are found to be essential in both investigations.

Here we also describe a probable transmission of SARS-CoV-2 from children to adult staff members in the school setting, namely from students to staff C during lessons on day 5 or day 6. Children are not suspected to be a significant risk of infection for school staff [37], but they seem to have a higher potential to transmit SARS-CoV-2 in household contacts [38]. We could confirm a low infection risk for adults in school and a higher level of transmission to close contacts at home with three infections. Nevertheless, the infection rate in household contacts might have been positively influenced by the health department’s advice to isolate the infected children in their rooms and separate functional areas as far as possible.

Overall, the disease progression of the COVID-19 patients in this outbreak was mild and straightforward. Although children are known to be susceptible and able to transmit SARS-CoV-2, surveillance data of Europe showed lower rates of hospitalization, severe courses of the sickness, and reported deaths of children. The affected age group of children between 11 and 14 years in this outbreak was described as the second most common group (12–14 years) in the 14-day notification rate selected in Europe in September 2020 [35]. Meanwhile, since March 2021, a high increase of COVID-19 cases was observed in young people between 16 and 18 years old, and the second most increase was observed in children aged between 12 and 15 years [36].

Incubation periods of COVID-19 in children in the outbreak in Hamburg started at one day for a single student and went up to 9 days. One-day incubation periods are out of the typical statistical range of 2.33–17.6 days [39], but the WHO and other health institutions used an estimation from 1 to 14 days until the second quarter of 2021, which was later changed to 2–14 days. Perhaps it should still be considered in quarantine concepts that incubation periods of 1 day can rarely occur COVID-19 when facing the high virus loads and infectivity of the emerging VOCs like Delta or Omicron.

The PCR-mass testing revealed six SARS-CoV-2 positive students and one educational staff member without symptoms, who would not have been found otherwise. Although these persons are potential asymptomatic carriers, they are difficult to consider in epidemiological curves because of their lack of symptom onset. Some investigators exclude those cases in outbreak analysis and thus, only describe relatively small outbreaks [9]. A recent study in Belgium with weekly PCR-testing of a cohort in a primary school found numerous infections with SARS-CoV-2 at students and staff in the school setting, with transmission into households. The authors conclude that it is necessary to go on with intensified testing and protection measures [40].

It is impossible to determine how many infections of the students took place during the two to three days of lessons between the first SSE’s and the start of the quarantine. It cannot be ruled out that some children, who were infected during the lessons of staff A on day 3 or 4, spread the virus to other students of the same class with a later onset of symptoms in school on day 5 or 6. Two or three days after the virus exposure in lessons, the students were still in close contact without mouth/nose protection in classrooms or with simple cotton masks in the restrooms, washrooms, and corridors. The fact that only four students of classes, who were not present in the lessons of staff A, were infected highlights that most infections are associated with these lessons. Further sporadic infections did not play a significant role in the transmission during this outbreak (Figure 1).

### 4.1. Genomic Sequencing Data Supporting Epidemiological Findings

Excluding one exception, the sequencing of all samples confirmed the same SARS-CoV-2 virus lineage B.1.177. This was the first recorded case of this lineage in the city at the time, but later sequencing revealed another single related sequence that had been sampled the week before the outbreak. In 2021 a further single related sequence was found in Hamburg, submitted without detailed information. The circulation of related viruses in Hamburg fits the researched source of infection of the index case from this school outbreak. This was a sportive superspreading event, which followed another SSE with international attendance. In addition, the phylogenetic analyses showed that the similar sequences from Hamburg, Spain and Denmark are not precursors or descendants of the outbreak cluster described here, which indicates that the outbreak was stopped successfully by the measurements taken.

Due to the high sequence similarity between the sequences of the outbreak samples it is impossible to derive detailed infection chains based on sequence data (Figure 2). Only four sequences of the cluster showed one or two unique point mutations, which were not detected in other sequenced samples. The lack of genetic divergence in most of the outbreak samples is likely due to the small size and short course of the outbreak as well as to the almost simultaneous sampling. The elaborated epidemiological data confirm the theory of explosive superspreading events with high transmission rates for short periods. Only a few unique mutations emerged in the viruses during the outbreak, but the low number of transmissions did not develop high genetic diversity. The analysis of the sequences revealed a cluster of a homogeneous outbreak from a single source.

### 4.2. Spatial Conditions and Ventilation

The outbreak was confined to an old school building, which, in parts, can be cramped and poorly ventilated. In the current pandemic, some windows that are usually closed for security reasons were opened to improve the ventilation. However, this way of improving the ventilation did not seem sufficient to avoid any aerosol transmissions in classrooms.

As partially shown in Figure 3, room 1 of class C.1 had only two windows with small ventilation areas and insufficient flow due to missing counter draft or wind because of the usually closed door. The temperature difference between the outside and inside of the room was low on that day, with a reported outside temperature of 20 ${}^{\circ }$C [41]. Apart from this, the windows were not regularly opened for an exchange of air. These determining factors reduced the quality of ventilation on that day. Additionally, the high number of people present in room 1 of class C.1 (29 people) led to an increase of virus-laden particles in the air and the long exposure time to a high concentration.

The seating plan of the first lesson on day 3 with class C.2 (Figure 4) showed a noticeable concentration of infected people on the window side of room 2, which might be correlated to the distribution of aerosols in the room. Several parameters can influence the aerosol concentration in the room under certain conditions. A follow-up examination of the indoor airflow conditions could be carried out in further research to clear up the detailed background of this constellation.

In classroom 3 of class C.3 (Figure 5) on the second day of lessons (day 4) from staff A, two infected students sat in the first row, close to A, while one of them sat on the corner of the second row. Close contacts are known to be more often infected, but the virus load in other places in the room enormously varies in different situations. The outdoor temperature on day 4 was 21 ${}^{\circ }$C [41]. The single infected student from class C.4 might have been infected by a friend of class C.1 in other situations but the infection was associated to the lesson on day 4. In the restrooms and washrooms, which are used by several students of different grades on the same corridor, the stipulated distances could often not be kept. The hygiene plan of that time demanded the washing or disinfecting hands before every lesson, which sometimes produced a cramped situation in the washrooms right before lessons. This was one of several possibilities for the infections of the three students of C.6 and C.7 and one student from class C.5, who were not taught by staff A. Further infections between students of classes C.1, C.2, C.3, and C.4 could also have happened in these locations, but this is less likely than the infection in the classroom.

Airborne transmissions via aerosol in longtime exposure of pupils without mouth/ nose protection in crowded and poorly ventilated classrooms led to most infections. Since the students did not wear masks in classrooms and inconsistently elsewhere indoors, infections could have also occurred via droplets in direct contact. Such indoor transmissions of SARS-CoV-2 turned out to be the main infection route in a Chinese public health evaluation of outbreaks. Here, transmissions in the home were prevalent, and outbreaks involving children were rare [38].

### 4.3. Lessons and Super Spreading Events

SSEs of various degrees happened during lessons on day 3 in classrooms, triggered by the same educational staff member and depending on several parameters (Figure 1, Table 2). Statistics revealed a significant difference (*p* < 0.05) in the day 3 vs. day 4 rate of infection (0.21 fraction infected/hour vs. 0.07 fraction infected/hour), but not between the respective classes. On the second day of lessons, when exposure times decreased and the index case changed behavior, only four infections followed in total after a total exposure time of 2.25 h, and the infection rate decreased approximately three-fold. These four lessons on 2 days provide a model with information on the decisive levers to prevent SSEs (Table 2).

Our findings suggest that the primary case was temporally highly infectious on these two days, especially on the first day. Several researchers claimed the highest virus load and infectivity of SARS-CoV-2 in the upper respiratory tract to be around or directly before the onset of symptoms [15,42,43]. Superspreaders or “superemitters” in SSEs are suspected to be individuals who are highly infectious because of a special biological constitution. One previous study has reported even individual differences at the level of aerosol production without SARS-CoV-2 infection. The production increased with Body Mass Index (BMI) and age [44], which did not match the primary case in this outbreak. Apart from the classroom events, staff A did not infect colleagues in the teachers or team rooms or private contacts, not even his housemate.

This outbreak reflects typical characteristics of the SARS-CoV-2 transmission: a highly infectious spreader one day before symptom onset, unaware of his infectivity, infecting many people in SSEs via an airborne transmission [16]. The dynamics of SARS-CoV-2 are shaped by SSEs in which disproportionally large numbers of people are infected by one source [14]. A coextensive study in India revealed a dominance of SSEs, where 5% of the infected people affected 80% of the cases [8].

During the lessons in classrooms on the first day, the educational staff A spoke loudly without a mask in front of the class in close distance to students in the first seating rows and, in the lesson of class C.1, to the second staff member B. Speaking loudly is known to produce increased amounts of aerosol particles, about eight to ten times higher than only breathing [17]. The dominance of the airborne transmission via droplets and aerosols, especially in overcrowded and poorly ventilated rooms, is already known [18]. Researchers agree that the indoor environment is of high importance not only during the pandemic [19].

The chronological overview (Figure 1) over the outbreak shows the compact course and the concentration of the infected cases in classes C.1 to C.4. Only a few infections took place outside this block and affected other single students of the classes C.5, C.6, C.7, and three close family members at home. The start of the first quarantine efficiently stopped further spreading in the school setting.

Until adequate protection is achieved in the community, it is necessary to wear a mask, keep distance, and avoid all conditions that promote infections with SARS-CoV-2. These conclusions are further supported by data from Belgium [40]. 

### 4.4. Limitations

This was the first time that a local health department in Hamburg, Germany, has used whole-genome sequencing of SARS-CoV-2 positive samples for outbreak analysis. Fifteen different laboratories made primary diagnostic PCR tests and positive samples were distributed to at least seven laboratories. Only three of them still could send samples to the sequencing laboratory. Therefore, sequence results are only available for most students and one parent but not for the educational staff and other family members. The primary case had to be inferred through detailed epidemiological follow-up. Especially, the SARS-CoV-2-positive people without available sequence, who had no known direct contact with staff A, are difficult to correlate to the outbreak. In these cases, exact dates of infection and incubation periods were partially missing. Two students of the class C.6, whose room is next to the classroom of class C.2, were probably part of the outbreak, but this is not clearly verifiable.

## 5. Conclusions

A transmission of SARS-CoV-2 from educational staff to students and other staff, among prepubescent students and, probably via a student-to-staff route, in a school setting and outside to close family members took place. Two major superspreading events with the airborne transmission in classrooms caused most of the infections, one day before the onset of symptoms at the primary case. Further infections independent of the mentioned events occurred, but probably only a few. The number of infected people in classrooms varied in different lessons of the infectious staff member, depending on several factors. These were the duration of exposure, the use of mouth/nose protection and the behavior of the infectious person with the way of speaking and keeping of safe distances, the quality of ventilation, and the number of people present. All measures that limit the number of exposed people before or after the first infections are suitable to limit the outbreak. These include preventive downsizing of functional units (e.g., classes, working groups, team meetings) or separating of bigger groups.

Cohorting grade levels as part of the safety concept in schools could limit the distribution of the virus in the setting. Consistent measures of the health department helped to contain the outbreak, such as mass testing and immediate quarantines for the whole cluster of affected grade levels, their educational staff, close contacts, and their families. Only three family contacts were infected, and no entry of the detected virus lineage in the population has been observed.

Asymptomatic spreaders, especially pre-symptomatic spreaders with high virus loads as the index case here, could be detected in finely resolving test concepts. These findings support the importance of modern concepts with bundle measures against SARS-CoV-2 and show tools to prevent schools from being pandemic drivers.

### Perspective

Independent of the COVID-19 pandemic ventilation concepts for large common rooms in schools, preferably via controlled ventilation systems, should be established for general infection prevention and as a part of a healthy learning environment. Education on the basics of hygiene could be firmly integrated into the lesson plan of adolescent students as a necessary competence and part of general infection prevention.

## Figures and Tables

**Table 1:** People involved in the affected school building as well as related family members, laboratory diagnostics and clinical data and quarantine measures.

	Students Aged 11–14 Years	Staff Members	Family Contacts
Total of tested persons	368	117	93
SARS-CoV-2 PCR + from the total No.	33 (8.96%) f:17, m:16	3 (1.69%)	7 (7.52%)
Outbreak associated of total	31 (8.42%) f:16, m:15	3 (1.69%)	3 (3.22%)
Sequenced from positive *	25 (75.75%)	0	1 (14.28%)
Same sequence cluster *	24 (72.72%)	0	1 (14.28%)
COVID-19 symptoms *	26 (78.78%)	2 (66.66%)	7 (100%)
Without symptoms *	7 (21.21%)	1 (33.33%)	0
Incubation period in days	1–9	4–5	2–4
No. of affected classes	7	4	2
Close contacts with the quarantine of 14 days	238	40	93
After exclusion of negative tested persons **	151	29	93

f = female, m= male. * Percentage based on the number of SARS-CoV-2 PCR positive tested persons. ** Quarantine was stopped for students and staff after negative test results of SARS-CoV-2 PCR.

**Table 2:** Conditions, behavior, and statistics during the lessons of staff A (index case) on day 3 and day 4.

Conditions, Behavior and Statistics	Lesson 1 Class C.2	Lesson 2 Class C.1	Lesson 3 Class C.3	Lesson 4 Class C.4
**Staff A: personal condition + conduct**				
Showing symptoms	−	−	−	+
Speaking loudly in front of class	++	+++	+	+
Estimated amount of time spent speaking	∼60%	>60%	<50%	<40%
Keeping distance to students	−	−	+	+
**Use of mouth-nose-protection (MNP)**				
Staff A				
In near contact with students at their places	+	+	+	+
In front of the class teaching	−	−	+	+
Students in classrooms	−	−	−	−
**Spatial conditions/ventilation**				
Classroom	2	1	3 (like 2)	4 (like 2)
No. of normal windows to be opened (always open at breaks, sporadically)	2/3 large	2/6 small	2/3 large	2/3 large
No. of open window flaps (always open)	3/3 large	4/6 small	3/3 large	3/3 large
Room volume m${}^{3}$	157	154	157	157
Open door	+/−	−	+/−	+/−
Smallest distance between the seats	0.60 m	0.60 m	0.60 m	0.60 m
Defined ventilation intervals	−	−	−	−
Close contact: possible droplet infection	+	+	−	−
**Statistics**				
Exposure time	1:30 h	3 h	1:30 h	0:45 h
No. of present people	25	29	25	28
No. of known infectious people	1	1	1	1
No. of infected susceptible people	8	16	3	1
Attack rate (AR)	33.33%	57.14%	12.5%	3.7%
Infection rate (1/h)	0.22	0.19	0.08	0.05
Pooled infection rate (1/h) on day 3 vs. day 4	0.21		0.07	
Hazard ratio (HR), day 3 vs. day 4	2.73 (1.23–6.07) *			

* p-value < 0.05. The infection rate was 0.22 and 0.19 (1/h) for the two classes on day 3 vs. 0.08 and 0.05 (1/h) for the classes on day 4. It was significantly larger on day 3 vs. day 4 (hazard ratio 2.73 [1.23–6.07], p-value = 0.0298), but insignificantly different for the respective classes that took place on the same day. +++, ++, +: the amount of time A reportedly spent speaking in front of the class.

## Data Availability

All sequence data and reconstructed genomes used in this study were uploaded to https://doi.org/10.17605/OSF.IO/KCRM7 (accessed on 23 November 2021).

## Supplementary Materials

The following are available online at https://www.mdpi.com/article/10.3390/v14010087/s1, Figure S1: View of window fronts, Table S1: GISAID acknowledgment.
