# Supplementary material for: Investigation of a Limited but Explosive COVID-19 Outbreak in a German Secondary School
Source: Viruses. 2022 Jan 4;14(1):87. doi: 10.3390/v14010087 (PMC8780098; doi:10.3390/v14010087)
Supplement: Supplementary file 1 [file viruses-14-00087-s001.zip › gisaid_acknowledgement.pdf]

We gratefully acknowledge the following Authors from the Originating laboratories responsible for obtaining the specimens, as well as the Submitting laboratories where the genome data were generated and shared via GISAID, on which this research is based.

All Submitters of data may be contacted directly via www.gisaid.org

Authors are sorted alphabetically.

Accession ID	Originating Laboratory	Submitting Laboratory	Authors
EPI_ISL_1410761	1. Glowny Inspektorat Sanitarny. 2. Diagnostyka. Laboratoria Medyczne.	1. ViroGenetics - BSL3 Laboratory of Virology, Malopolska Centre of Biotechnology, Jagiellonian University; 2. genXone SA, Research & Development Labor Laboratory	Aleksandra Gidlewicz; Anna Brylak; Gromowski, T.; Grzegorz Nowicki; Jakub Grabowski; Karol Szeszko; Kowalski, M.; Labaj; Lukasz Krych; Maciej Sykulski; Mazur-Panasiuk, N.; Michal Kaszuba; Natalia Drweska-Matelska; P.P.; Pyrc, K.; Sylwia Januszczak; Szulc, P.
EPI_ISL_1265418	1. Wojewódzka Stacja Sanitarno - Epidemiologiczna w Katowicach (WSSE Katowice); 2. Wojewódzka Stacja Sanitarno - Epidemiologiczna w Kielcach (WSSE Kielce); 3. Wojewódzka Stacja Sanitarno - Epidemiologiczna w Szczecinie (WSSE Szczecin); 4. Wojewódzka Stacja Sanitarno - Epidemiologiczna w Lublinie (WSSE Lublin); 5. Wojewódzka Stacja Sanitarno - Epidemiologiczna w Gorzowie Wielkopolskim (WSSE Gorzów Wielkopolski); 6. Wojewódzka Stacja Sanitarno-Epidemiologiczna w Gdańsku (WSSE Gdańsk); 7. Wojewódzka Stacja Sanitarno-Epidemiologiczna w Poznaniu (WSSE Poznań); 8. Wojewódzka Stacja Sanitarno-Epidemiologiczna w Opolu (WSSE Opole); 9. Wojewódzka Stacja Sanitarno-Epidemiologiczna we Wrocławiu (WSSE Wrocław); 10. Wojewódzka Stacja Sanitarno-Epidemiologiczna w Krakowie (WSSE Kraków); 11. Wojewódzka Stacja Sanitarno-Epidemiologiczna w Olsztynie (WSSE Olsztyn); 12. Wojewódzka Stacja Sanitarno-Epidemiologiczna w Bydgoszczy (WSSE Bydgoszcz); 13. Wojewódzka Stacja Sanitarno-Epidemiologiczna w Rzeszowie (WSSE Rzeszów); 14. Wojewódzka Stacja Sanitarno-Epidemiologiczna w Białymstoku (WSSE Białystok);	1. ViroGenetics - BSL3 Laboratory of Virology, Małopolska Centre of Biotechnology, Jagiellonian University; 2. Human Genome Variation Research Group, Malopolska Centre of Biotechnology, Jagiellonian University;	Branicki, W.; Gromowski, T.; Klajmon, A.; Kowalski, M.; Labaj; Marszalek, K.; Mazur-Panasiuk, N.; P.P.; Pyrc, K.; Szulc, P.
EPI_ISL_1166073	24023 PLATEFORME MGI COVID	CNR Virus des Infections Respiratoires - France SUD	Antonin Bal; Bruno Lina; Gregory Destras; Gwendolyne Burfin; Hadrien Regue; Laurence Josset; Martine Valette; Quentin Semanas
EPI_ISL_1840895	3. Medizinische Abteilung, Hanusch Krankenhaus	Bergthaler laboratory, CeMM Research Center for Molecular Medicine of the Austrian Academy of Sciences	Andreas Bergthaler; Anna Schedl; Bekir Erguner; Benedikt Agerer; Christoph Bock; Fabian Amman; Jan Laine; Lukas Endler; Maelle Le Moing; Martin Senekowitsch; Michael Schuster; Petr Triska; Thomas Penz
EPI_ISL_729733	A. Krumbholz, Labor Dr. Krause und Kollegen MVZ GmbH, Kiel	Charité Universitätsmedizin Berlin, Institut für Virologie	Barbara Mühlemann; Christian Drosten; Julia Schneider; Jörn Beheim-Schwarzbach; Talitha Veith; Terry Jones; Victor M Corman
EPI_ISL_2359205	ADMED	Laboratory of genomics and metagenomics	Claire Bertelli; Damien Jacot; Gilbert Greub; Sébastien Aeby; Trestan Pillonel
EPI_ISL_2491572	Akershus University Hospital, Department for Microbiology and Infectious Disease Control	Norwegian Institute of Public Health, Department of Virology	Atiya R Ali; Debech Nadia; Engebretsen Serina Beate; Garcia Llorente Ignacio; Hilde Elshaug; Hilde Vollan; Jon Bråte; Kamilla Heddeland Instefjord; Karoline Bragstad; Kathrine Stene-Johansen; Line Victoria Moen; Marie Paulsen Madsen; Olav Hungnes; Pedersen Benedikte Nevjen; Rasmus Riis Kopperud
EPI_ISL_2158333	Alab Laboratoria Sp. z o.o.	1. National Institute of Public Health - National Institute of Hygiene; 2. Eurofins Genomics Europe Sequencing GmbH	ECDC COVID-19 WGS support team; Eurofins Genomics Europe Sequencing Team; Gierczyński Rafał; Sadkowska-Todys Małgorzata; Wołkowicz Tomasz; Zacharczuk Katarzyna
EPI_ISL_1008167	Austrian Agency for Health and Food Safety (AGES)	Bergthaler laboratory, CeMM Research Center for Molecular Medicine of the Austrian Academy of Sciences	Andreas Bergthaler; Anna Schedl; Bekir Erguner; Benedikt Agerer; Christoph Bock; Jan Laine; Lukas Endler; Maelle Le Moing; Martin Senekowitsch; Michael Schuster; Thomas Penz
EPI_ISL_1185839, EPI_ISL_1222806	Azienda Sanitaria dell'Alto Adige Laboratorio Aziendale di Microbiologia e Virologia	Istituto di Genomica Applicata	Davide Scaglione; Eleonora Paparelli; Elisa Masi; Elisabetta Giacobazzi; Elisabetta Pagani; Gabriele Magris; Irena Jurman; Irene Bianconi; Michele Morgante; Stefanie Wieser; Vera Vendramin
EPI_ISL_529217	Beijing Institute of Microbiology and Epidemiology	Beijing Institute of Microbiology and Epidemiology	Cui, Y.; Fan; Guo, Y.; Hang; Hou, J.; Li, B.; Mi, Z.; Mu, J.; Qin, E.; Song; Teng; Wu, Y.; Xu, Z.; Yajun.; Yang, R.; Yong, Y.; Yue; Zhang, X.
EPI_ISL_2128588	Bioinformatics and Biostatistics Lab, Advanced Sequencing Facility	COVID-19 Genomics UK (COG-UK) Consortium	Aengus Stewart; Chelsea Sawyer; Harshil Patel; Jerome Nicod; Laura Cubitt; Margaret Crawford
EPI_ISL_2270072, EPI_ISL_2270076	Biolytix AG	Clinical Bacteriology	Adrian Egli; Alfredo Mari; Hans Hirsch; Helena MB Seth-Smith; Julia Bielicki; Karoline Leuzinger; Madlen Stange; Manuel Battegay; Tim Roloff
EPI_ISL_2232078, EPI_ISL_2232081, EPI_ISL_2232082, EPI_ISL_2232084, EPI_ISL_2232085, EPI_ISL_2232086, EPI_ISL_2232087			
see above	Biopathology - Clinical Microbiology, Department Clinical and Laboratory Research, University of Thessaly	Greek Genome Center, Biomedical Research Foundation of the Academy of Athens (BRFAA)	Dimitrios Thanos; Efthimia Petinaki; Emmanouil Athanasiadis; Giannis Vatsellas; Katerina Zoi; Theodoros Loupis
EPI_ISL_1141449, EPI_ISL_1211312, EPI_ISL_1216253, EPI_ISL_1843063, EPI_ISL_1848742	Bioscientia Labor Wermsdorf	Robert Koch Institute	
EPI_ISL_1843558, EPI_ISL_1843635, EPI_ISL_1843691, EPI_ISL_1847302, EPI_ISL_1848685, EPI_ISL_2109326	Bioscientia MVZ Labor Karlsruhe GmbH	Robert Koch Institute	
EPI_ISL_732531, EPI_ISL_732532, EPI_ISL_732534, EPI_ISL_732535, EPI_ISL_732536, EPI_ISL_732537, EPI_ISL_732538, EPI_ISL_732539, EPI_ISL_732540, EPI_ISL_732541, EPI_ISL_732544, EPI_ISL_732550, EPI_ISL_732551, EPI_ISL_732553, EPI_ISL_732554, EPI_ISL_732555, EPI_ISL_732556, EPI_ISL_732557, EPI_ISL_732558, EPI_ISL_732559, EPI_ISL_732561, EPI_ISL_732564, EPI_ISL_732658			
see above	Bundeswehr Institute of Microbiology	Bundeswehr Institute of Microbiology	Alexandra Rehn; Elham Khatamzas; Enrico Georgi; Malena Bestehorn-Willmann; Markus Antwerpen; Mathias Walter; Michael von Bergwelt-Baildon; Roman Wölfel; Sabine Zange
EPI_ISL_1833975	Bundeswehrzentralkrankenhaus Koblenz	Bundeswehr Institute of Microbiology	Alexandra Rehn; Enrico Georgi; Malena Bestehorn-Willmann; Markus Antwerpen; Mathias Walter; Mike Pillukat; Roman Wölfel; Sabine Zange
EPI_ISL_1322387	C.H.R.U. MONTPIED	CNR Virus des Infections Respiratoires - France SUD	Antonin Bal; Bruno Lina; Gregory Destras; Gwendolyne Burfin; Hadrien Regue; Laurence Josset; Martine Valette; Quentin Semanas
EPI_ISL_1757728	CH. CLEMENCEAU	Department of Virology, Henri Mondor University Hospital, Assistance Publique Hôpitaux de Paris, Université Paris-Est Créteil, INSERM U955	Alexandre Soulier; Christophe Rodriguez; Elisabeth Trawinski; Guillaume Gricourt; Jean-Michel Pawlotsky; Melissa N'Debi; Slim Fourati; Vanessa Demontant
EPI_ISL_2278673, EPI_ISL_2351581	CHU Purpan - Laboratoire de Virologie - Institut Fédératif de Biologie	CHU Purpan - Laboratoire de Virologie - Institut Fédératif de Biologie	Bulach T.; Donnadieu C.; Izopet J.; Latour J.; Milhes M.; Nicot F.; Ranger N.; Salin G.; Tremeaux P.
EPI_ISL_2359198, EPI_ISL_2359470, EPI_ISL_2359478	CHUV	Laboratory of genomics and metagenomics	Claire Bertelli; Damien Jacot; Gilbert Greub; Sébastien Aeby; Trestan Pillonel
EPI_ISL_1908193	CONSEJERIA DE SANIDAD Y ASUNTOS SOCIALES	Instituto de Salud Carlos III	A. Monzón; F. Casas; GONZALO; I. GUTIERREZ AVILA; I. Jiménez; M. Camarero; P. Zaballos; S. Cuesta; S. Iglesias-Caballero; S. Pozo; S. Varona; Sandonís; V. Vázquez-Morón
EPI_ISL_523950	Center of Medical Microbiology, Virology, and Hospital Hygiene, University of Duesseldorf	Center of Medical Microbiology, Virology, and Hospital Hygiene, Heinrich Heine University Düsseldorf	Alexander Dilthey; Andreas Walker; Daniel Strelow; Hendrik Streeck; Jessica Nicolai; Jörg Timm; Klaus Pfeffer; Malte Kohns Vasconcelos; Marek Korencak; Maximilian Damagnez; Tobias Wienemann; Torsten Houwaart
EPI_ISL_413488, EPI_ISL_414497, EPI_ISL_414498, EPI_ISL_414499, EPI_ISL_414504, EPI_ISL_414505, EPI_ISL_414506, EPI_ISL_414508, EPI_ISL_414509, EPI_ISL_414574, EPI_ISL_417459, EPI_ISL_417462, EPI_ISL_419546, EPI_ISL_425126, EPI_ISL_425131, EPI_ISL_523937, EPI_ISL_523946, EPI_ISL_523947, EPI_ISL_523948, EPI_ISL_602515, EPI_ISL_602533, EPI_ISL_602536, EPI_ISL_602539, EPI_ISL_832366, EPI_ISL_1346719, EPI_ISL_1346744, EPI_ISL_1346754, EPI_ISL_1346764, EPI_ISL_1346768, EPI_ISL_1346778, EPI_ISL_1346944, EPI_ISL_1346952, EPI_ISL_1346959, EPI_ISL_1347208, EPI_ISL_1347211, EPI_ISL_1347229, EPI_ISL_1988673, EPI_ISL_1990025			
see above	Center of Medical Microbiology, Virology, and Hospital Hygiene, University of Duesseldorf	Center of Medical Microbiology, Virology, and Hospital Hygiene, University of Duesseldorf	Alexander Dilthey; Andreas Walker; Ashley-Jane Duplessis; Bjorn-Erik Jensen; Björn-Erik Jensen; Daniel Strelow; Detlef Kindgen-Milles; Hendrik Streeck; Jessica Nicolai; Jorg Timm; Jörg Timm; Klaus Pfeffer; Lisanna Hülse; Malte Kohns Vasconcelos; Marcel Andree; Marek Korencak; Maximilian Damagnez; Nadine Lübke; Ortwin Adams; Sandra Hauka; Teresa Tamayo; Tina Senff; Tobias Wienemann; Torsten Feldt; Torsten Houwaart
EPI_ISL_815260, EPI_ISL_815261, EPI_ISL_815262, EPI_ISL_815264, EPI_ISL_815265, EPI_ISL_815266, EPI_ISL_815267, EPI_ISL_815268, EPI_ISL_815270, EPI_ISL_815271, EPI_ISL_815274, EPI_ISL_815275, EPI_ISL_815277, EPI_ISL_815281, EPI_ISL_815282, EPI_ISL_815284, EPI_ISL_815285, EPI_ISL_815286, EPI_ISL_815289, EPI_ISL_815290, EPI_ISL_815291, EPI_ISL_815293, EPI_ISL_815295, EPI_ISL_815296, EPI_ISL_815297, EPI_ISL_815299, EPI_ISL_815300, EPI_ISL_815301, EPI_ISL_815302, EPI_ISL_815304, EPI_ISL_815312, EPI_ISL_815313, EPI_ISL_815314, EPI_ISL_815315, EPI_ISL_815317, EPI_ISL_815321, EPI_ISL_815323, EPI_ISL_815324, EPI_ISL_815325, EPI_ISL_815327, EPI_ISL_815331, EPI_ISL_815332, EPI_ISL_815334, EPI_ISL_815336, EPI_ISL_815338, EPI_ISL_815339, EPI_ISL_815341, EPI_ISL_815342, EPI_ISL_815344, EPI_ISL_815345, EPI_ISL_815346, EPI_ISL_815349, EPI_ISL_815350, EPI_ISL_815352, EPI_ISL_815353, EPI_ISL_815356, EPI_ISL_815357, EPI_ISL_815361, EPI_ISL_815364, EPI_ISL_815365, EPI_ISL_815366, EPI_ISL_815368, EPI_ISL_815369, EPI_ISL_815370, EPI_ISL_815371, EPI_ISL_815372, EPI_ISL_815373, EPI_ISL_815374, EPI_ISL_815376, EPI_ISL_815377, EPI_ISL_815379, EPI_ISL_815380, EPI_ISL_815381, EPI_ISL_815384, EPI_ISL_815385, EPI_ISL_815386, EPI_ISL_815387, EPI_ISL_815388, EPI_ISL_815389			
see above	Centogene	Centogene	Krishna Kumar Kandaswamy; Peter Bauer; Vivi Hue-Trang Lieu
EPI_ISL_457750, EPI_ISL_459962,	Centogene AG	Centogene AG	Dr. Krishna Kumar Kandaswamy; Prof. Dr. Peter Bauer

EPI_ISL_459963, EPI_ISL_459964			
EPI_ISL_2214925	Centre Hospitalier Universitaire Clermont-Ferrand	CHU Clermont-Ferrand, service de virologie	Bisseux Maxime; Combes Patricia; Henquell Cécile; Mirand Audrey
EPI_ISL_2354656	Centre for Clinical Infection and Diagnostics Research and Genomics Innovation Unit, Guy's and St. Thomas' NHS Trust	COVID-19 Genomics UK (COG-UK) Consortium	Ali Raza Awan; Chloe Fisher; Jonathan Edgeworth; Luke Snell; Penny Cliff; Rahul Batra
EPI_ISL_841866, EPI_ISL_1979903, EPI_ISL_2127945, EPI_ISL_2127950, EPI_ISL_2128097, EPI_ISL_2128124	Centre for Enzyme Innovation, University of Portsmouth / Translational Research Laboratory, Portsmouth Hospitals NHS Trust	COVID-19 Genomics UK (COG-UK) Consortium	Allyson Lloyd; Angela Beckett; Anoop Chauhan; Christopher Fearn; Ethan Butcher; Garry Scarlett; Kate Cook; Katie Loveson; Kelly Bicknell; Robert Impey; Salman Goudarzi; Samuel Robson; Sarah Wyllie; Scott Elliott; Sharon Glaysher; Yann Bourgeois
EPI_ISL_2149035, EPI_ISL_2149054	Centro Nacional de Gripe (Valladolid)	SeqCOVID-SPAIN consortium/IBV(CSIC)	Diana Perez San José and SeqCOVID-SPAIN consortium; Iván Sanz Muñoz
EPI_ISL_1849228	Clemenshospital - Alexianer Clemenshospital MÃ¼nster	Robert Koch Institute	
EPI_ISL_2367217	Clinical Virology	Clinical Bacteriology	Adrian Egli; Alfredo Mari; Fanny Wegner; Hans Hirsch; Helena MB Seth-Smith; Julia Bielicki; Karoline Leuzinger; Manuel Battegay; Tim Roloff
EPI_ISL_2035563, EPI_ISL_2035734	Clinical Virology	Clinical Virology	Anissa Chouikha; Henda Triki; Kais Ghedira; Mariem Gdoura; Sondos Haddad; Wasfi Fares; Wasfi Fraes
EPI_ISL_700492	Conville CDC wc CVC	NHLS/UCT	Arash Iranzadeh; Bruna Galvao; Carolyn Williamson; Deelan Doolabh; Diana Hardie; Houriiyah Tegally; Innocent Mudau; Kruger Marais; Lynn Tyers; Marvin Hsiao; Stephen Korsman
EPI_ISL_2308901	Croatian Institute of Public Health	Croatian Institute of Public Health	Irena Tabain; Ivana Ferenčak
EPI_ISL_949235, EPI_ISL_1938886, EPI_ISL_1939012, EPI_ISL_1939014, EPI_ISL_1939015, EPI_ISL_1939022, EPI_ISL_1939031, EPI_ISL_2147325, EPI_ISL_2271116, EPI_ISL_2271118, EPI_ISL_2271121, EPI_ISL_2271122, EPI_ISL_2271123, EPI_ISL_2271127, EPI_ISL_2271130, EPI_ISL_2271243, EPI_ISL_2465703, EPI_ISL_2465709, EPI_ISL_2465711, EPI_ISL_2465719			
see above	Departamento de Microbiología, CDB, Hospital Clínic, Barcelona	SeqCOVID-SPAIN consortium/IBV(CSIC)	Aida Peiró and SeqCOVID-SPAIN consortium; Andrea Vergara; Elisa Rubio; Jéssica Navero; Mikel Martínez
EPI_ISL_2432247	Department of Bacteria, Parasites and Fungi, Statens Serum Institut, Copenhagen, Denmark	Statens Serum Institut Bioinformatics and Microbial Genomics	Danish Covid-19 Genome Consortium
EPI_ISL_872070	Department of Clinical Microbiology	GIGA Medical Genomics	Bouchra Boujemla; Cécile Meex; Keith Durkin; Maria Artesi; Marie-Pierre Hayette; Pierrette Melin; Raphaël Boreux; Sébastien Bontems; Vincent Bours
EPI_ISL_1866507, EPI_ISL_1873531, EPI_ISL_1875912, EPI_ISL_1876795, EPI_ISL_1881460, EPI_ISL_1887349, EPI_ISL_1891473, EPI_ISL_1892464			
see above	Department of Clinical Microbiology and Center for Genomic Medicine, Rigshospitalet, Copenhagen, Denmark	Aalborg University	Danish Covid-19 Genome Consortium
EPI_ISL_1866607, EPI_ISL_1873130, EPI_ISL_1885642	Department of Clinical Microbiology, Odense University Hospital, Odense, Denmark	Aalborg University	Danish Covid-19 Genome Consortium
EPI_ISL_2137099, EPI_ISL_2137111	Department of Laboratory Medicine, Division of Clinical Virology, University of Medicine, Vienna	Bergthaler laboratory, CeMM Research Center for Molecular Medicine of the Austrian Academy of Sciences	Andreas Bergthaler; Anna Schedl; Bekir Erguner; Benedikt Agerer; Christoph Bock; Fabian Amman; Jan Laine; Lukas Endler; Maelle Le Moing; Martin Senekowitsch; Michael Schuster; Petr Triska; Thomas Penz
EPI_ISL_2333494	Department of Medical Microbiology, St. Olavs hospital	Norwegian Institute of Public Health, Department of Virology	'Kathrine Stene-Johansen; Atiya R Ali; Debech Nadia; Engebretsen Serina Beate; Garcia Llorente Ignacio; Hilde Elshaug; Hilde Vollan; Jon Bråte; Kamilla Heddeland Instefjord; Karoline Bragstad; Line Victoria Moen; Marie Paulsen Madsen; Olav Hungnes; Pedersen Benedikte Nevjen; Rasmus Riis Kopperud
EPI_ISL_1164660, EPI_ISL_1164665, EPI_ISL_1164685, EPI_ISL_1164694	Department of Molecular Virology, Cyprus Institute of Neurology and Genetics	Department of Molecular Virology, Cyprus Institute of Neurology and Genetics	Anastasis Oulas; Andreas Hadjisavvas; Christina Christodoulou; Christina Tryfonos; Dana Koptides; Denise Alexandrou; George Krashias; George Spyrou; Jan Richter; Maria Loizidou; Mihalis Panayiotidis; Olga Kalakouta; Pavlos Fanis; Stavros Bashiardes
EPI_ISL_679158, EPI_ISL_813159	Department of Pathology, University of Cambridge	COVID-19 Genomics UK (COG-UK) Consortium	Aminu S. Jahun; Grant Hall; Ian Goodfellow; Iliana Georgana; Malte Pinckert; Martin D. Curran; Myra Hosmillo; Surendra Parmar; Yasmin Chaudhry
EPI_ISL_1841556, EPI_ISL_1841614, EPI_ISL_1841809, EPI_ISL_1842053, EPI_ISL_1842121	Department of Virology and Immunology, University of Helsinki and Helsinki University Hospital, Huslab Finland	Department of Virology, Faculty of Medicine, University of Helsinki, Helsinki, Finland	Essi Korhonen; Hanna Jarva; Hanna Liimatainen; Hannimari Kallio-Kokko; Harri Kangas; Hussein Alburkat; Jenni Virtanen; Maija Lappalainen; Maija Suvanto; Olli Vapalahti; Pekka Ellonen; Phuoc Truong; Ravi Kant; Sari Hannula; Satu Kurkela; Teemu Smura
EPI_ISL_1015291	Department of Virology, Pitié-Salpêtrière hospital	Department of Virology, Pitié-Salpêtrière hospital	Anne-Geneviève Marcelin; Aude Jary; Karen Zafilaza; Stéphane Marot; Valentin Leducq; Vincent Calvez
EPI_ISL_855616, EPI_ISL_855623, EPI_ISL_855718, EPI_ISL_855737, EPI_ISL_855744, EPI_ISL_855748, EPI_ISL_855756, EPI_ISL_855779, EPI_ISL_855785, EPI_ISL_855787, EPI_ISL_855792, EPI_ISL_855799, EPI_ISL_855806, EPI_ISL_855837, EPI_ISL_855869, EPI_ISL_855871, EPI_ISL_855873, EPI_ISL_855915, EPI_ISL_855924, EPI_ISL_855982, EPI_ISL_855991, EPI_ISL_855996, EPI_ISL_856007, EPI_ISL_856015, EPI_ISL_856019, EPI_ISL_856040, EPI_ISL_856189, EPI_ISL_856222, EPI_ISL_856374, EPI_ISL_856420, EPI_ISL_856443, EPI_ISL_856535, EPI_ISL_856563, EPI_ISL_856602, EPI_ISL_856631, EPI_ISL_856642, EPI_ISL_856671, EPI_ISL_856675, EPI_ISL_869425, EPI_ISL_869485, EPI_ISL_869585, EPI_ISL_869606, EPI_ISL_869662, EPI_ISL_869715, EPI_ISL_869724, EPI_ISL_869741, EPI_ISL_869808, EPI_ISL_869819, EPI_ISL_869866, EPI_ISL_869898, EPI_ISL_870071, EPI_ISL_870171, EPI_ISL_870250, EPI_ISL_870262, EPI_ISL_870285, EPI_ISL_870292, EPI_ISL_870420, EPI_ISL_870441, EPI_ISL_870489, EPI_ISL_870555, EPI_ISL_870572, EPI_ISL_870589, EPI_ISL_870618, EPI_ISL_870632, EPI_ISL_870646, EPI_ISL_870681, EPI_ISL_870715, EPI_ISL_870738, EPI_ISL_870796, EPI_ISL_870806, EPI_ISL_870867, EPI_ISL_870876, EPI_ISL_870898, EPI_ISL_870912, EPI_ISL_870919, EPI_ISL_870961, EPI_ISL_870969, EPI_ISL_870981, EPI_ISL_871025, EPI_ISL_871048, EPI_ISL_871083, EPI_ISL_871214, EPI_ISL_871228, EPI_ISL_871241, EPI_ISL_871242, EPI_ISL_871253, EPI_ISL_871257, EPI_ISL_871265, EPI_ISL_871281, EPI_ISL_871294, EPI_ISL_871371, EPI_ISL_871401, EPI_ISL_871414, EPI_ISL_871423, EPI_ISL_871525, EPI_ISL_871571, EPI_ISL_871659, EPI_ISL_871663, EPI_ISL_871731, EPI_ISL_925979, EPI_ISL_925983, EPI_ISL_926103, EPI_ISL_926146, EPI_ISL_926264, EPI_ISL_926432, EPI_ISL_926559, EPI_ISL_926593, EPI_ISL_926644, EPI_ISL_926683, EPI_ISL_926845, EPI_ISL_926941, EPI_ISL_926976, EPI_ISL_927009, EPI_ISL_927015, EPI_ISL_927161, EPI_ISL_927165, EPI_ISL_927191, EPI_ISL_927207, EPI_ISL_927209, EPI_ISL_927240, EPI_ISL_927247, EPI_ISL_927369, EPI_ISL_927411, EPI_ISL_927459, EPI_ISL_927477, EPI_ISL_927526, EPI_ISL_927562, EPI_ISL_927571, EPI_ISL_927573, EPI_ISL_927593, EPI_ISL_927611, EPI_ISL_927620, EPI_ISL_927663, EPI_ISL_927703, EPI_ISL_927720, EPI_ISL_927766, EPI_ISL_927778, EPI_ISL_927863, EPI_ISL_928009, EPI_ISL_928045, EPI_ISL_928068, EPI_ISL_928130, EPI_ISL_928212, EPI_ISL_928254, EPI_ISL_928272, EPI_ISL_928285, EPI_ISL_928349, EPI_ISL_928420, EPI_ISL_928440, EPI_ISL_928444, EPI_ISL_928609, EPI_ISL_928618, EPI_ISL_928724, EPI_ISL_928817, EPI_ISL_928961, EPI_ISL_928975, EPI_ISL_929053, EPI_ISL_929086, EPI_ISL_929143, EPI_ISL_929152, EPI_ISL_929257, EPI_ISL_929388, EPI_ISL_929433, EPI_ISL_929438, EPI_ISL_929464, EPI_ISL_929528, EPI_ISL_929529, EPI_ISL_929534, EPI_ISL_929545, EPI_ISL_929699, EPI_ISL_929738, EPI_ISL_929758, EPI_ISL_929833, EPI_ISL_929875, EPI_ISL_929919, EPI_ISL_929937, EPI_ISL_929995, EPI_ISL_930010, EPI_ISL_930350, EPI_ISL_930441, EPI_ISL_930444, EPI_ISL_930474, EPI_ISL_971140, EPI_ISL_971169, EPI_ISL_971226, EPI_ISL_971498, EPI_ISL_971586, EPI_ISL_971717, EPI_ISL_971801, EPI_ISL_971842, EPI_ISL_971877, EPI_ISL_971890, EPI_ISL_972111, EPI_ISL_972164, EPI_ISL_972237, EPI_ISL_972246, EPI_ISL_972263, EPI_ISL_972270, EPI_ISL_972596, EPI_ISL_972697, EPI_ISL_972752, EPI_ISL_972805, EPI_ISL_972816, EPI_ISL_972893, EPI_ISL_972917, EPI_ISL_972929, EPI_ISL_972987, EPI_ISL_972994, EPI_ISL_973028, EPI_ISL_973040, EPI_ISL_973056, EPI_ISL_973207, EPI_ISL_973309, EPI_ISL_973754, EPI_ISL_1023672, EPI_ISL_1023970, EPI_ISL_1024020, EPI_ISL_1024618, EPI_ISL_1024946, EPI_ISL_1025096, EPI_ISL_1025168, EPI_ISL_1025198, EPI_ISL_1025299, EPI_ISL_1025340, EPI_ISL_1025402, EPI_ISL_1025404, EPI_ISL_1025405, EPI_ISL_1025428, EPI_ISL_1025526, EPI_ISL_1025549, EPI_ISL_1025559, EPI_ISL_1025580, EPI_ISL_1025604, EPI_ISL_1025613, EPI_ISL_1025615, EPI_ISL_1025617, EPI_ISL_1025685, EPI_ISL_1025689, EPI_ISL_1025697, EPI_ISL_1025701, EPI_ISL_1025712, EPI_ISL_1025729, EPI_ISL_1025782, EPI_ISL_1025784, EPI_ISL_1025804, EPI_ISL_1025829, EPI_ISL_1025831, EPI_ISL_1025837, EPI_ISL_1025894, EPI_ISL_1025955, EPI_ISL_1065103, EPI_ISL_1065156, EPI_ISL_1065171, EPI_ISL_1065213, EPI_ISL_1065313, EPI_ISL_1065327, EPI_ISL_1065343, EPI_ISL_1065370, EPI_ISL_1065376, EPI_ISL_1065382, EPI_ISL_1065395, EPI_ISL_1065428, EPI_ISL_1065452, EPI_ISL_1065458, EPI_ISL_1065464, EPI_ISL_1065466, EPI_ISL_1065518, EPI_ISL_1065528, EPI_ISL_1065562, EPI_ISL_1065582, EPI_ISL_1065585, EPI_ISL_1065613, EPI_ISL_1065702, EPI_ISL_1065780, EPI_ISL_1066017, EPI_ISL_1066052, EPI_ISL_1066074, EPI_ISL_1066090, EPI_ISL_1066107, EPI_ISL_1066159, EPI_ISL_1066210, EPI_ISL_1066239, EPI_ISL_1066242, EPI_ISL_1066244, EPI_ISL_1066250, EPI_ISL_1066319, EPI_ISL_1066387, EPI_ISL_1066448, EPI_ISL_1066467, EPI_ISL_1066511, EPI_ISL_1066555, EPI_ISL_1066569, EPI_ISL_1066609, EPI_ISL_1066629, EPI_ISL_1066634, EPI_ISL_1066650, EPI_ISL_1066682, EPI_ISL_1066901, EPI_ISL_1066943, EPI_ISL_1066978, EPI_ISL_1066980, EPI_ISL_1067007, EPI_ISL_1067136, EPI_ISL_1067192, EPI_ISL_1067210, EPI_ISL_1067235, EPI_ISL_1067248, EPI_ISL_1067281, EPI_ISL_1067380, EPI_ISL_1067382, EPI_ISL_1067437, EPI_ISL_1067444, EPI_ISL_1067445, EPI_ISL_1067569, EPI_ISL_1124169, EPI_ISL_1124312, EPI_ISL_1124473, EPI_ISL_1125432, EPI_ISL_1125528, EPI_ISL_1125668, EPI_ISL_1125722, EPI_ISL_1125744, EPI_ISL_1125920, EPI_ISL_1126077, EPI_ISL_1126699, EPI_ISL_1126753, EPI_ISL_1127072, EPI_ISL_1858002, EPI_ISL_1858195, EPI_ISL_1858285, EPI_ISL_1858750, EPI_ISL_1858932, EPI_ISL_1859015, EPI_ISL_1859161, EPI_ISL_1859274, EPI_ISL_1859411, EPI_ISL_1859491, EPI_ISL_1859695, EPI_ISL_1860073, EPI_ISL_1860541, EPI_ISL_1860569, EPI_ISL_1860683, EPI_ISL_1860753, EPI_ISL_1860824, EPI_ISL_1860870, EPI_ISL_1860897, EPI_ISL_1860952, EPI_ISL_1860991, EPI_ISL_1861075, EPI_ISL_1861080, EPI_ISL_1861405, EPI_ISL_1861674, EPI_ISL_1862437, EPI_ISL_1862481, EPI_ISL_1862621, EPI_ISL_1862653, EPI_ISL_1862753, EPI_ISL_1863011, EPI_ISL_1863381, EPI_ISL_1863422, EPI_ISL_1863424, EPI_ISL_1863462, EPI_ISL_1863735, EPI_ISL_1864225, EPI_ISL_1864227, EPI_ISL_1864868, EPI_ISL_1864901, EPI_ISL_1864972, EPI_ISL_1865025, EPI_ISL_1865063, EPI_ISL_1865150, EPI_ISL_1865211, EPI_ISL_1865322, EPI_ISL_1865399, EPI_ISL_1865505, EPI_ISL_1865578, EPI_ISL_1865655, EPI_ISL_1865721, EPI_ISL_1866013, EPI_ISL_1866101, EPI_ISL_1866200, EPI_ISL_1866388, EPI_ISL_1866425, EPI_ISL_1866435, EPI_ISL_1866442, EPI_ISL_1866499, EPI_ISL_1866503, EPI_ISL_1866589, EPI_ISL_1866627, EPI_ISL_1866719, EPI_ISL_1866926, EPI_ISL_1867202, EPI_ISL_1867403, EPI_ISL_1867697, EPI_ISL_1867803, EPI_ISL_1867868, EPI_ISL_1867925, EPI_ISL_1868106, EPI_ISL_1868292, EPI_ISL_1868298, EPI_ISL_1868493, EPI_ISL_1868746, EPI_ISL_1868885, EPI_ISL_1868913, EPI_ISL_1868984, EPI_ISL_1869118, EPI_ISL_1869138, EPI_ISL_1869230, EPI_ISL_1869275, EPI_ISL_1869367, EPI_ISL_1869374, EPI_ISL_1869382, EPI_ISL_1869445, EPI_ISL_1869654, EPI_ISL_1869834, EPI_ISL_1870035, EPI_ISL_1870050, EPI_ISL_1870157, EPI_ISL_1870176, EPI_ISL_1870192, EPI_ISL_1870216, EPI_ISL_1870330, EPI_ISL_1870367, EPI_ISL_1870476, EPI_ISL_1870614, EPI_ISL_1870651, EPI_ISL_1870658, EPI_ISL_1870719, EPI_ISL_1870723, EPI_ISL_1871030, EPI_ISL_1871383, EPI_ISL_1871562, EPI_ISL_1871622, EPI_ISL_1871817, EPI_ISL_1871834, EPI_ISL_1871907, EPI_ISL_1872164, EPI_ISL_1872207, EPI_ISL_1872239, EPI_ISL_1872251, EPI_ISL_1872343, EPI_ISL_1872609, EPI_ISL_1872634, EPI_ISL_1872663, EPI_ISL_1872699, EPI_ISL_1872760, EPI_ISL_1872850, EPI_ISL_1872926, EPI_ISL_1873197, EPI_ISL_1873376, EPI_ISL_1873584, EPI_ISL_1873793, EPI_ISL_1873923, EPI_ISL_1873948, EPI_ISL_1874057, EPI_ISL_1874114, EPI_ISL_1874300, EPI_ISL_1874311, EPI_ISL_1874447, EPI_ISL_1874451, EPI_ISL_1874523, EPI_ISL_1874601, EPI_ISL_1874659, EPI_ISL_1874724, EPI_ISL_1874743, EPI_ISL_1874803, EPI_ISL_1874834, EPI_ISL_1874946, EPI_ISL_1875053, EPI_ISL_1875261, EPI_ISL_1875394, EPI_ISL_1875501, EPI_ISL_1875737, EPI_ISL_1875794, EPI_ISL_1876276, EPI_ISL_1876412, EPI_ISL_1876452, EPI_ISL_1876487, EPI_ISL_1876561, EPI_ISL_1876622, EPI_ISL_1876634, EPI_ISL_1876844, EPI_ISL_1876993, EPI_ISL_1877063, EPI_ISL_1877125, EPI_ISL_1877168, EPI_ISL_1877218, EPI_ISL_1877272, EPI_ISL_1877622, EPI_ISL_1877855, EPI_ISL_1878021, EPI_ISL_1878029, EPI_ISL_1878059, EPI_ISL_1878173, EPI_ISL_1878328, EPI_ISL_1878510, EPI_ISL_1878593, EPI_ISL_1878619, EPI_ISL_1878655, EPI_ISL_1878696, EPI_ISL_1878752, EPI_ISL_1878757, EPI_ISL_1878828, EPI_ISL_1878913, EPI_ISL_1878981, EPI_ISL_1879054, EPI_ISL_1879175, EPI_ISL_1879215, EPI_ISL_1879331, EPI_ISL_1879430, EPI_ISL_1879710, EPI_ISL_1879791, EPI_ISL_1879865, EPI_ISL_1879977, EPI_ISL_1880029, EPI_ISL_1880031, EPI_ISL_1880212, EPI_ISL_1880224, EPI_ISL_1880260, EPI_ISL_1880294, EPI_ISL_1880489, EPI_ISL_1880528, EPI_ISL_1880576, EPI_ISL_1880579, EPI_ISL_1880692, EPI_ISL_1880773, EPI_ISL_1880942, EPI_ISL_1880990, EPI_ISL_1881227, EPI_ISL_1881860, EPI_ISL_1881902, EPI_ISL_1881972, EPI_ISL_1882347, EPI_ISL_1882499, EPI_ISL_1882612, EPI_ISL_1882803, EPI_ISL_1882850, EPI_ISL_1883044, EPI_ISL_1883073, EPI_ISL_1883106, EPI_ISL_1883109, EPI_ISL_1883138, EPI_ISL_1883152, EPI_ISL_1883154, EPI_ISL_1883208, EPI_ISL_1883232, EPI_ISL_1883356, EPI_ISL_1883458, EPI_ISL_1883520, EPI_ISL_1883697, EPI_ISL_1883723, EPI_ISL_1883781, EPI_ISL_1883855, EPI_ISL_1884274, EPI_ISL_1884394, EPI_ISL_1884588, EPI_ISL_1885015, EPI_ISL_1885110, EPI_ISL_1885156, EPI_ISL_1885182, EPI_ISL_1885247, EPI_ISL_1885370, EPI_ISL_1885474, EPI_ISL_1885638, EPI_ISL_1885669, EPI_ISL_1885845, EPI_ISL_1885878, EPI_ISL_1886139, EPI_ISL_1886376, EPI_ISL_1886392, EPI_ISL_1886399, EPI_ISL_1886534, EPI_ISL_1886545, EPI_ISL_1886587, EPI_ISL_1886829, EPI_ISL_1886842, EPI_ISL_1886944, EPI_ISL_1887022, EPI_ISL_1887347, EPI_ISL_1887447, EPI_ISL_1887448, EPI_ISL_1887590, EPI_ISL_1887603, EPI_ISL_1887665, EPI_ISL_1887891, EPI_ISL_1887931, EPI_ISL_1887961, EPI_ISL_1888147, EPI_ISL_1888180, EPI_ISL_1888266, EPI_ISL_1888409, EPI_ISL_1888441, EPI_ISL_1888535, EPI_ISL_1888593, EPI_ISL_1888749, EPI_ISL_1888765, EPI_ISL_1888771, EPI_ISL_1888829, EPI_ISL_1888850, EPI_ISL_1888941, EPI_ISL_1888980, EPI_ISL_1889065, EPI_ISL_1889209, EPI_ISL_1889223, EPI_ISL_1889224, EPI_ISL_1889464, EPI_ISL_1889629, EPI_ISL_1889639, EPI_ISL_1889869, EPI_ISL_1890013, EPI_ISL_1890173, EPI_ISL_1890401, EPI_ISL_1890670, EPI_ISL_1890719, EPI_ISL_1890790, EPI_ISL_1890850, EPI_ISL_1890862, EPI_ISL_1890913, EPI_ISL_1891100, EPI_ISL_1891274, EPI_ISL_1891613, EPI_ISL_1891729, EPI_ISL_1891730, EPI_ISL_1891856, EPI_ISL_1891909, EPI_ISL_1892313, EPI_ISL_1892449, EPI_ISL_1892606, EPI_ISL_1892669, EPI_ISL_1893252, EPI_ISL_1893296, EPI_ISL_1893640, EPI_ISL_1893654, EPI_ISL_1893682, EPI_ISL_1893845, EPI_ISL_1893891, EPI_ISL_1893926, EPI_ISL_1894048, EPI_ISL_1894188, EPI_ISL_1894289, EPI_ISL_1894332, EPI_ISL_1894414, EPI_ISL_1894471, EPI_ISL_1894549, EPI_ISL_1894589, EPI_ISL_1894594, EPI_ISL_1894846, EPI_ISL_1894908, EPI_ISL_1894933, EPI_ISL_1895219, EPI_ISL_1895224, EPI_ISL_1895248, EPI_ISL_1895344, EPI_ISL_1895463, EPI_ISL_1895845, EPI_ISL_1895919, EPI_ISL_1896053, EPI_ISL_1896307, EPI_ISL_1896319, EPI_ISL_2023525			
see above	Department of Virus and Microbiological Special Diagnostics, Statens Serum Institut, Copenhagen, Denmark	Aalborg University	Danish Covid-19 Genome Consortium
EPI_ISL_668602, EPI_ISL_668603, EPI_ISL_668646, EPI_ISL_668647, EPI_ISL_668648, EPI_ISL_668651, EPI_ISL_668652, EPI_ISL_668653, EPI_ISL_668654, EPI_ISL_668657, EPI_ISL_668660, EPI_ISL_668662, EPI_ISL_668668, EPI_ISL_668671, EPI_ISL_668678, EPI_ISL_668681, EPI_ISL_668684, EPI_ISL_668686, EPI_ISL_668687, EPI_ISL_668689, EPI_ISL_668693, EPI_ISL_668696, EPI_ISL_668698, EPI_ISL_668704, EPI_ISL_668706, EPI_ISL_668707, EPI_ISL_668709, EPI_ISL_668710, EPI_ISL_669089, EPI_ISL_669297, EPI_ISL_669536, EPI_ISL_669537, EPI_ISL_669538, EPI_ISL_669541, EPI_ISL_669542, EPI_ISL_669543, EPI_ISL_669614, EPI_ISL_669617, EPI_ISL_669621, EPI_ISL_669628, EPI_ISL_669630, EPI_ISL_669631, EPI_ISL_669632, EPI_ISL_669643, EPI_ISL_669646, EPI_ISL_669649, EPI_ISL_669657, EPI_ISL_669658, EPI_ISL_669659, EPI_ISL_669663, EPI_ISL_669664, EPI_ISL_669665, EPI_ISL_669671, EPI_ISL_669672, EPI_ISL_669674, EPI_ISL_669680, EPI_ISL_669681, EPI_ISL_669682, EPI_ISL_669686, EPI_ISL_669688, EPI_ISL_669691, EPI_ISL_669696, EPI_ISL_669697, EPI_ISL_669698, EPI_ISL_669701, EPI_ISL_669702, EPI_ISL_669705, EPI_ISL_669707, EPI_ISL_669713, EPI_ISL_669715, EPI_ISL_669722, EPI_ISL_669723, EPI_ISL_669724, EPI_ISL_669725, EPI_ISL_669729, EPI_ISL_670524, EPI_ISL_670525, EPI_ISL_670527, EPI_ISL_670559, EPI_ISL_670561, EPI_ISL_670562, EPI_ISL_670563, EPI_ISL_670564, EPI_ISL_670567, EPI_ISL_670568, EPI_ISL_670569, EPI_ISL_670570, EPI_ISL_670571, EPI_ISL_670572, EPI_ISL_670577, EPI_ISL_670582, EPI_ISL_670584, EPI_ISL_670585, EPI_ISL_670587, EPI_ISL_670588, EPI_ISL_670590, EPI_ISL_670597, EPI_ISL_670598, EPI_ISL_670599, EPI_ISL_670604, EPI_ISL_670606, EPI_ISL_670611, EPI_ISL_670612, EPI_ISL_670618, EPI_ISL_670620, EPI_ISL_670621, EPI_ISL_670625, EPI_ISL_670626, EPI_ISL_670627, EPI_ISL_670633, EPI_ISL_670636, EPI_ISL_670637, EPI_ISL_670645, EPI_ISL_670648, EPI_ISL_670658, EPI_ISL_671091, EPI_ISL_682378, EPI_ISL_682382, EPI_ISL_682398, EPI_ISL_682416, EPI_ISL_682435, EPI_ISL_682440, EPI_ISL_682449, EPI_ISL_682489, EPI_ISL_682517, EPI_ISL_682524, EPI_ISL_682538, EPI_ISL_682557, EPI_ISL_682571, EPI_ISL_682625, EPI_ISL_682635, EPI_ISL_682689, EPI_ISL_682712, EPI_ISL_682718, EPI_ISL_682763, EPI_ISL_682768, EPI_ISL_682772, EPI_ISL_682788, EPI_ISL_682798, EPI_ISL_682805, EPI_ISL_682866, EPI_ISL_682904, EPI_ISL_682921, EPI_ISL_682942, EPI_ISL_682953, EPI_ISL_682965, EPI_ISL_682973, EPI_ISL_682995, EPI_ISL_711305, EPI_ISL_711306, EPI_ISL_711307, EPI_ISL_711308, EPI_ISL_711309, EPI_ISL_711312, EPI_ISL_711313, EPI_ISL_711314, EPI_ISL_711317, EPI_ISL_711319, EPI_ISL_711323, EPI_ISL_711324, EPI_ISL_711325, EPI_ISL_711327, EPI_ISL_711328, EPI_ISL_711329, EPI_ISL_711330, EPI_ISL_711331, EPI_ISL_711332, EPI_ISL_711333, EPI_ISL_711334, EPI_ISL_711335, EPI_ISL_711336, EPI_ISL_711337, EPI_ISL_711338, EPI_ISL_711342, EPI_ISL_711344, EPI_ISL_711346, EPI_ISL_711348, EPI_ISL_711349, EPI_ISL_711351, EPI_ISL_711352, EPI_ISL_711355, EPI_ISL_711359, EPI_ISL_711360, EPI_ISL_711362, EPI_ISL_711365, EPI_ISL_711366, EPI_ISL_711367, EPI_ISL_711370, EPI_ISL_711373, EPI_ISL_711374, EPI_ISL_711375, EPI_ISL_711377, EPI_ISL_711378, EPI_ISL_711379, EPI_ISL_711382, EPI_ISL_711383, EPI_ISL_712340, EPI_ISL_712341, EPI_ISL_712342, EPI_ISL_712345, EPI_ISL_712346, EPI_ISL_712347, EPI_ISL_712348, EPI_ISL_712349, EPI_ISL_712350, EPI_ISL_712351, EPI_ISL_712353, EPI_ISL_712354, EPI_ISL_712356, EPI_ISL_712358, EPI_ISL_712359, EPI_ISL_712360, EPI_ISL_712361, EPI_ISL_712362, EPI_ISL_712364, EPI_ISL_712367, EPI_ISL_712368, EPI_ISL_712369, EPI_ISL_712370, EPI_ISL_712371, EPI_ISL_712372, EPI_ISL_712375, EPI_ISL_712376, EPI_ISL_712378, EPI_ISL_712380, EPI_ISL_712382, EPI_ISL_712385, EPI_ISL_712387, EPI_ISL_712388, EPI_ISL_712390, EPI_ISL_712395, EPI_ISL_712398, EPI_ISL_712399, EPI_ISL_712400, EPI_ISL_712401, EPI_ISL_712403, EPI_ISL_712405, EPI_ISL_712406, EPI_ISL_712408, EPI_ISL_712409, EPI_ISL_712411, EPI_ISL_712412, EPI_ISL_712414, EPI_ISL_712415, EPI_ISL_713332, EPI_ISL_713333, EPI_ISL_713335, EPI_ISL_713337, EPI_ISL_713338, EPI_ISL_713339, EPI_ISL_713340, EPI_ISL_713341, EPI_ISL_713347, EPI_ISL_713348, EPI_ISL_713350, EPI_ISL_713353, EPI_ISL_713354, EPI_ISL_713357, EPI_ISL_713360, EPI_ISL_713362, EPI_ISL_713365, EPI_ISL_713366, EPI_ISL_713367, EPI_ISL_713369, EPI_ISL_713372, EPI_ISL_713375, EPI_ISL_713378, EPI_ISL_713380, EPI_ISL_713383, EPI_ISL_713384, EPI_ISL_713386, EPI_ISL_713387, EPI_ISL_713389, EPI_ISL_713390, EPI_ISL_713392, EPI_ISL_713396, EPI_ISL_713397, EPI_ISL_713398, EPI_ISL_713399, EPI_ISL_713400, EPI_ISL_713401, EPI_ISL_713403, EPI_ISL_713408, EPI_ISL_713409, EPI_ISL_713414, EPI_ISL_713417, EPI_ISL_713419, EPI_ISL_713420, EPI_ISL_713421, EPI_ISL_713422, EPI_ISL_713749, EPI_ISL_713934, EPI_ISL_714300, EPI_ISL_714301, EPI_ISL_714303, EPI_ISL_714304, EPI_ISL_714306, EPI_ISL_714307, EPI_ISL_714308, EPI_ISL_714310, EPI_ISL_714313, EPI_ISL_714314, EPI_ISL_714315, EPI_ISL_714317, EPI_ISL_714322, EPI_ISL_714326, EPI_ISL_714327, EPI_ISL_714330, EPI_ISL_714331, EPI_ISL_714333, EPI_ISL_714334, EPI_ISL_714335, EPI_ISL_714339, EPI_ISL_714340, EPI_ISL_714342, EPI_ISL_714345, EPI_ISL_714347, EPI_ISL_714348, EPI_ISL_714349, EPI_ISL_714352, EPI_ISL_714353, EPI_ISL_714354, EPI_ISL_714355, EPI_ISL_714357, EPI_ISL_714358, EPI_ISL_714359, EPI_ISL_714360, EPI_ISL_714363, EPI_ISL_714554, EPI_ISL_747681, EPI_ISL_747683, EPI_ISL_747684, EPI_ISL_747686, EPI_ISL_747687, EPI_ISL_747688, EPI_ISL_747690, EPI_ISL_747692, EPI_ISL_747693, EPI_ISL_747697, EPI_ISL_747698, EPI_ISL_747700, EPI_ISL_747701, EPI_ISL_747702, EPI_ISL_747704, EPI_ISL_747705, EPI_ISL_747706, EPI_ISL_747707, EPI_ISL_747710, EPI_ISL_747711, EPI_ISL_747712, EPI_ISL_747713, EPI_ISL_747716, EPI_ISL_747717, EPI_ISL_747718, EPI_ISL_747720, EPI_ISL_748055, EPI_ISL_748384, EPI_ISL_748385, EPI_ISL_748386, EPI_ISL_748389, EPI_ISL_748390, EPI_ISL_748394, EPI_ISL_748395, EPI_ISL_748397, EPI_ISL_748398, EPI_ISL_748399, EPI_ISL_748400, EPI_ISL_748401, EPI_ISL_748404, EPI_ISL_748405, EPI_ISL_748407, EPI_ISL_748408, EPI_ISL_748411, EPI_ISL_748412, EPI_ISL_748416, EPI_ISL_748419, EPI_ISL_748420, EPI_ISL_748421, EPI_ISL_748426, EPI_ISL_748427, EPI_ISL_748428, EPI_ISL_748429, EPI_ISL_748430, EPI_ISL_748431, EPI_ISL_748432, EPI_ISL_748435, EPI_ISL_748436, EPI_ISL_748438, EPI_ISL_748441, EPI_ISL_748442, EPI_ISL_748444, EPI_ISL_748446, EPI_ISL_748449, EPI_ISL_748450, EPI_ISL_748452, EPI_ISL_748454, EPI_ISL_748455, EPI_ISL_748456, EPI_ISL_748457, EPI_ISL_748458, EPI_ISL_748460, EPI_ISL_748461, EPI_ISL_748462, EPI_ISL_748464, EPI_ISL_748466, EPI_ISL_748675, EPI_ISL_749414, EPI_ISL_749415,			

EPI_ISL_749418, EPI_ISL_749419, EPI_ISL_749420, EPI_ISL_749422, EPI_ISL_749423, EPI_ISL_749424, EPI_ISL_749426, EPI_ISL_749428, EPI_ISL_749431, EPI_ISL_749432, EPI_ISL_749435, EPI_ISL_749437, EPI_ISL_749438, EPI_ISL_749440, EPI_ISL_749441, EPI_ISL_749442, EPI_ISL_749445, EPI_ISL_749446, EPI_ISL_749447, EPI_ISL_749448, EPI_ISL_749454, EPI_ISL_749456, EPI_ISL_749458, EPI_ISL_749459, EPI_ISL_749460, EPI_ISL_749463, EPI_ISL_749465, EPI_ISL_749468, EPI_ISL_749469, EPI_ISL_749472, EPI_ISL_749473, EPI_ISL_749475, EPI_ISL_749476, EPI_ISL_749478, EPI_ISL_749479, EPI_ISL_749483, EPI_ISL_750408, EPI_ISL_750411, EPI_ISL_750413, EPI_ISL_750414, EPI_ISL_750416, EPI_ISL_750417, EPI_ISL_750419, EPI_ISL_750421, EPI_ISL_750426, EPI_ISL_750427, EPI_ISL_750428, EPI_ISL_750429, EPI_ISL_750433, EPI_ISL_750434, EPI_ISL_750437, EPI_ISL_750438, EPI_ISL_750444, EPI_ISL_750445, EPI_ISL_750448, EPI_ISL_750450, EPI_ISL_750451, EPI_ISL_750453, EPI_ISL_750454, EPI_ISL_750456, EPI_ISL_750458, EPI_ISL_750459, EPI_ISL_750460, EPI_ISL_750462, EPI_ISL_750466, EPI_ISL_750467, EPI_ISL_750469, EPI_ISL_750470, EPI_ISL_750471, EPI_ISL_750472, EPI_ISL_750473, EPI_ISL_750481, EPI_ISL_750483, EPI_ISL_750484, EPI_ISL_750499, EPI_ISL_750500, EPI_ISL_750501, EPI_ISL_757471, EPI_ISL_757472, EPI_ISL_757475, EPI_ISL_757476, EPI_ISL_757477, EPI_ISL_757479, EPI_ISL_757480, EPI_ISL_757481, EPI_ISL_757482, EPI_ISL_757484, EPI_ISL_757486, EPI_ISL_757487, EPI_ISL_757488, EPI_ISL_757975, EPI_ISL_757977, EPI_ISL_757982, EPI_ISL_757983, EPI_ISL_757987, EPI_ISL_757989, EPI_ISL_757992, EPI_ISL_757994, EPI_ISL_757997, EPI_ISL_758001, EPI_ISL_758002, EPI_ISL_758004, EPI_ISL_758009, EPI_ISL_758010, EPI_ISL_758019, EPI_ISL_758023, EPI_ISL_758024, EPI_ISL_758025, EPI_ISL_758026, EPI_ISL_758027, EPI_ISL_758029, EPI_ISL_758030, EPI_ISL_758032, EPI_ISL_758037, EPI_ISL_758040, EPI_ISL_758041, EPI_ISL_758043, EPI_ISL_758045, EPI_ISL_758046, EPI_ISL_758047, EPI_ISL_758048, EPI_ISL_758050, EPI_ISL_758051, EPI_ISL_758055, EPI_ISL_758057, EPI_ISL_758058, EPI_ISL_758059, EPI_ISL_758060, EPI_ISL_758062, EPI_ISL_758063, EPI_ISL_758977, EPI_ISL_758978, EPI_ISL_758980, EPI_ISL_758981, EPI_ISL_758983, EPI_ISL_758985, EPI_ISL_758986, EPI_ISL_758988, EPI_ISL_758990, EPI_ISL_758992, EPI_ISL_758995, EPI_ISL_758998, EPI_ISL_759001, EPI_ISL_759004, EPI_ISL_759006, EPI_ISL_759009, EPI_ISL_759010, EPI_ISL_759013, EPI_ISL_759017, EPI_ISL_759019, EPI_ISL_759022, EPI_ISL_759026, EPI_ISL_759030, EPI_ISL_759034, EPI_ISL_759035, EPI_ISL_759038, EPI_ISL_759039, EPI_ISL_759040, EPI_ISL_759064, EPI_ISL_792799, EPI_ISL_792801, EPI_ISL_792805, EPI_ISL_792806, EPI_ISL_792808, EPI_ISL_792811, EPI_ISL_792812, EPI_ISL_792816, EPI_ISL_792819, EPI_ISL_792821, EPI_ISL_792822, EPI_ISL_792823, EPI_ISL_792824, EPI_ISL_792826, EPI_ISL_792827, EPI_ISL_793322, EPI_ISL_793325, EPI_ISL_793326, EPI_ISL_793327, EPI_ISL_793328, EPI_ISL_793336, EPI_ISL_793337, EPI_ISL_793340, EPI_ISL_793353, EPI_ISL_793360, EPI_ISL_793361, EPI_ISL_793362, EPI_ISL_793367, EPI_ISL_793369, EPI_ISL_794389, EPI_ISL_794391, EPI_ISL_794392, EPI_ISL_794395, EPI_ISL_794397, EPI_ISL_794398, EPI_ISL_794399, EPI_ISL_794402, EPI_ISL_794403, EPI_ISL_794404, EPI_ISL_794407, EPI_ISL_794410, EPI_ISL_794413, EPI_ISL_794414, EPI_ISL_794415, EPI_ISL_794416, EPI_ISL_794417, EPI_ISL_794418, EPI_ISL_794419, EPI_ISL_794885, EPI_ISL_794886, EPI_ISL_794888, EPI_ISL_794889, EPI_ISL_794891, EPI_ISL_794892, EPI_ISL_794894, EPI_ISL_794897, EPI_ISL_794899, EPI_ISL_795283, EPI_ISL_795284, EPI_ISL_795285, EPI_ISL_795287, EPI_ISL_795288, EPI_ISL_795290, EPI_ISL_795295, EPI_ISL_795296, EPI_ISL_795298, EPI_ISL_795301, EPI_ISL_795303, EPI_ISL_795304, EPI_ISL_795305, EPI_ISL_795312, EPI_ISL_795316, EPI_ISL_795318, EPI_ISL_795322, EPI_ISL_795327, EPI_ISL_795329, EPI_ISL_795336, EPI_ISL_795337, EPI_ISL_795338, EPI_ISL_795339, EPI_ISL_795341, EPI_ISL_795342, EPI_ISL_795346, EPI_ISL_795348, EPI_ISL_795351, EPI_ISL_795355, EPI_ISL_795358, EPI_ISL_795360, EPI_ISL_795365, EPI_ISL_795366, EPI_ISL_795381, EPI_ISL_815564, EPI_ISL_815573, EPI_ISL_815576, EPI_ISL_815577, EPI_ISL_815580, EPI_ISL_815582, EPI_ISL_815583, EPI_ISL_815587, EPI_ISL_815590, EPI_ISL_815592, EPI_ISL_815594, EPI_ISL_815597, EPI_ISL_815598, EPI_ISL_815599, EPI_ISL_815600, EPI_ISL_815601, EPI_ISL_815603, EPI_ISL_815625, EPI_ISL_817415, EPI_ISL_817416, EPI_ISL_817418, EPI_ISL_817423, EPI_ISL_817424, EPI_ISL_817431, EPI_ISL_817435, EPI_ISL_817437, EPI_ISL_817439, EPI_ISL_817441, EPI_ISL_817443, EPI_ISL_817447, EPI_ISL_817449, EPI_ISL_817450, EPI_ISL_817451, EPI_ISL_817452, EPI_ISL_817455, EPI_ISL_817458, EPI_ISL_817459, EPI_ISL_817462, EPI_ISL_817464, EPI_ISL_817465, EPI_ISL_817467, EPI_ISL_817469, EPI_ISL_817471, EPI_ISL_817474, EPI_ISL_817480, EPI_ISL_817481, EPI_ISL_818372, EPI_ISL_818374, EPI_ISL_818378, EPI_ISL_818385, EPI_ISL_818387, EPI_ISL_818390, EPI_ISL_818394, EPI_ISL_818395, EPI_ISL_818398, EPI_ISL_818400, EPI_ISL_818408, EPI_ISL_818409, EPI_ISL_818411, EPI_ISL_818417, EPI_ISL_818418, EPI_ISL_818422, EPI_ISL_818425, EPI_ISL_818431, EPI_ISL_818434, EPI_ISL_818437, EPI_ISL_818443, EPI_ISL_843236, EPI_ISL_843292, EPI_ISL_843354, EPI_ISL_843406, EPI_ISL_843536, EPI_ISL_843718, EPI_ISL_843785, EPI_ISL_843892, EPI_ISL_843910, EPI_ISL_844046, EPI_ISL_844097, EPI_ISL_844123, EPI_ISL_844132, EPI_ISL_844135, EPI_ISL_844150, EPI_ISL_844156, EPI_ISL_844161, EPI_ISL_844189, EPI_ISL_844270, EPI_ISL_844271, EPI_ISL_844272, EPI_ISL_844276, EPI_ISL_844277, EPI_ISL_844285, EPI_ISL_844289, EPI_ISL_844290, EPI_ISL_844292, EPI_ISL_844293, EPI_ISL_844294, EPI_ISL_844861, EPI_ISL_844863, EPI_ISL_844865, EPI_ISL_844868, EPI_ISL_844869, EPI_ISL_844871, EPI_ISL_844874, EPI_ISL_844875, EPI_ISL_844876, EPI_ISL_844877, EPI_ISL_844879, EPI_ISL_844880, EPI_ISL_844881, EPI_ISL_844883, EPI_ISL_844884, EPI_ISL_844886, EPI_ISL_844887, EPI_ISL_844888, EPI_ISL_844893, EPI_ISL_844896, EPI_ISL_844898, EPI_ISL_844899, EPI_ISL_844901, EPI_ISL_844902, EPI_ISL_844906, EPI_ISL_844909, EPI_ISL_844910, EPI_ISL_844913, EPI_ISL_844915, EPI_ISL_844916, EPI_ISL_844918, EPI_ISL_847301, EPI_ISL_847303, EPI_ISL_847305, EPI_ISL_847306, EPI_ISL_847307, EPI_ISL_847311, EPI_ISL_847315, EPI_ISL_847320, EPI_ISL_847321, EPI_ISL_847322, EPI_ISL_847324, EPI_ISL_847325			
see above	Department of Virus and Microbiological Special Diagnostics, Statens Serum Institut, Copenhagen, Denmark	Albertsen Lab, Department of Chemistry and Bioscience, Aalborg University, Denmark	Danish Covid-19 Genome Consortium
EPI_ISL_615176, EPI_ISL_615182, EPI_ISL_615183, EPI_ISL_615211, EPI_ISL_615212, EPI_ISL_615223, EPI_ISL_615241, EPI_ISL_615283, EPI_ISL_615285, EPI_ISL_615288, EPI_ISL_615290, EPI_ISL_615291, EPI_ISL_615292, EPI_ISL_615299, EPI_ISL_615308, EPI_ISL_615309, EPI_ISL_615310, EPI_ISL_615348, EPI_ISL_615374, EPI_ISL_615381, EPI_ISL_615382, EPI_ISL_615386, EPI_ISL_615389, EPI_ISL_615391, EPI_ISL_615394, EPI_ISL_615395, EPI_ISL_615396, EPI_ISL_615415, EPI_ISL_615424, EPI_ISL_615522, EPI_ISL_615582, EPI_ISL_615629, EPI_ISL_615632, EPI_ISL_615633, EPI_ISL_615634, EPI_ISL_615674, EPI_ISL_615680, EPI_ISL_615687, EPI_ISL_615689, EPI_ISL_615690, EPI_ISL_615691, EPI_ISL_615692, EPI_ISL_615693, EPI_ISL_615719, EPI_ISL_615721, EPI_ISL_615731, EPI_ISL_615761, EPI_ISL_615763, EPI_ISL_615767, EPI_ISL_615788, EPI_ISL_615790, EPI_ISL_615793, EPI_ISL_615794, EPI_ISL_615828, EPI_ISL_615857, EPI_ISL_615873, EPI_ISL_615885, EPI_ISL_615968, EPI_ISL_615978, EPI_ISL_615989, EPI_ISL_616017, EPI_ISL_616087, EPI_ISL_616113, EPI_ISL_616119, EPI_ISL_616148, EPI_ISL_616156, EPI_ISL_616205, EPI_ISL_616267, EPI_ISL_616315, EPI_ISL_616320, EPI_ISL_616324, EPI_ISL_616326, EPI_ISL_616328, EPI_ISL_616345, EPI_ISL_616368, EPI_ISL_616607, EPI_ISL_616713, EPI_ISL_616747, EPI_ISL_616797, EPI_ISL_616821, EPI_ISL_616840, EPI_ISL_616883, EPI_ISL_616889, EPI_ISL_616951, EPI_ISL_616980, EPI_ISL_616984, EPI_ISL_616987, EPI_ISL_616991, EPI_ISL_616992, EPI_ISL_616994, EPI_ISL_616996, EPI_ISL_617001, EPI_ISL_617005, EPI_ISL_617007, EPI_ISL_617010, EPI_ISL_617013, EPI_ISL_617015, EPI_ISL_617018, EPI_ISL_617020, EPI_ISL_617022, EPI_ISL_617023, EPI_ISL_617024, EPI_ISL_617025, EPI_ISL_617026, EPI_ISL_617028, EPI_ISL_617029, EPI_ISL_617032, EPI_ISL_617036, EPI_ISL_617037, EPI_ISL_617137, EPI_ISL_617800, EPI_ISL_617801, EPI_ISL_617914, EPI_ISL_617921, EPI_ISL_617935, EPI_ISL_618411, EPI_ISL_618412, EPI_ISL_618415, EPI_ISL_618450, EPI_ISL_618458, EPI_ISL_618480, EPI_ISL_618481, EPI_ISL_618492, EPI_ISL_618502, EPI_ISL_618505, EPI_ISL_618510, EPI_ISL_618548, EPI_ISL_618556, EPI_ISL_618579, EPI_ISL_618594, EPI_ISL_618600, EPI_ISL_618608, EPI_ISL_618668, EPI_ISL_618686, EPI_ISL_618692, EPI_ISL_618744, EPI_ISL_618749, EPI_ISL_618775, EPI_ISL_618781, EPI_ISL_618849, EPI_ISL_618879, EPI_ISL_618894, EPI_ISL_618983, EPI_ISL_618984, EPI_ISL_618985, EPI_ISL_618991, EPI_ISL_618992, EPI_ISL_618993, EPI_ISL_618994, EPI_ISL_618995, EPI_ISL_618996, EPI_ISL_618998, EPI_ISL_618999, EPI_ISL_619002, EPI_ISL_619010, EPI_ISL_619012, EPI_ISL_619013, EPI_ISL_619014, EPI_ISL_619015, EPI_ISL_619025, EPI_ISL_619032, EPI_ISL_619034, EPI_ISL_619035, EPI_ISL_619039, EPI_ISL_619043, EPI_ISL_619044, EPI_ISL_619045, EPI_ISL_619048, EPI_ISL_619049, EPI_ISL_619411, EPI_ISL_619420, EPI_ISL_619474, EPI_ISL_619484, EPI_ISL_619485, EPI_ISL_619489, EPI_ISL_619523, EPI_ISL_619524, EPI_ISL_619526, EPI_ISL_619528, EPI_ISL_619529, EPI_ISL_619530, EPI_ISL_619538, EPI_ISL_619591, EPI_ISL_619604, EPI_ISL_619610, EPI_ISL_619639, EPI_ISL_619640, EPI_ISL_619649, EPI_ISL_619653, EPI_ISL_619666, EPI_ISL_619678, EPI_ISL_619679, EPI_ISL_619682, EPI_ISL_619684, EPI_ISL_619685, EPI_ISL_619686, EPI_ISL_619704, EPI_ISL_619707, EPI_ISL_619710, EPI_ISL_619711, EPI_ISL_619769, EPI_ISL_619779, EPI_ISL_619825, EPI_ISL_619833, EPI_ISL_619837, EPI_ISL_619897, EPI_ISL_619930, EPI_ISL_620038, EPI_ISL_620047, EPI_ISL_620069, EPI_ISL_620075, EPI_ISL_620080, EPI_ISL_620094, EPI_ISL_620105, EPI_ISL_620110, EPI_ISL_620123, EPI_ISL_620163, EPI_ISL_620184, EPI_ISL_620215, EPI_ISL_620240, EPI_ISL_620241, EPI_ISL_620272, EPI_ISL_620289, EPI_ISL_620299, EPI_ISL_620350, EPI_ISL_620357, EPI_ISL_620358, EPI_ISL_620359, EPI_ISL_620363, EPI_ISL_620762, EPI_ISL_620763, EPI_ISL_620780, EPI_ISL_620786, EPI_ISL_620812, EPI_ISL_620818, EPI_ISL_620864, EPI_ISL_620877, EPI_ISL_620882, EPI_ISL_620884, EPI_ISL_620902, EPI_ISL_620954, EPI_ISL_620965, EPI_ISL_620974, EPI_ISL_621033, EPI_ISL_621036, EPI_ISL_621037, EPI_ISL_621076, EPI_ISL_621087, EPI_ISL_621090, EPI_ISL_621106, EPI_ISL_621119, EPI_ISL_621132, EPI_ISL_621155, EPI_ISL_621161, EPI_ISL_621162, EPI_ISL_621163, EPI_ISL_621167, EPI_ISL_621170, EPI_ISL_621171, EPI_ISL_621185, EPI_ISL_621221, EPI_ISL_621303, EPI_ISL_621310, EPI_ISL_621336, EPI_ISL_621341, EPI_ISL_621342, EPI_ISL_621353, EPI_ISL_621390, EPI_ISL_621453, EPI_ISL_621481, EPI_ISL_621483, EPI_ISL_621496, EPI_ISL_621501, EPI_ISL_621512, EPI_ISL_621570, EPI_ISL_621581, EPI_ISL_621638, EPI_ISL_621645, EPI_ISL_621679, EPI_ISL_621680, EPI_ISL_621681, EPI_ISL_621684, EPI_ISL_621692, EPI_ISL_621693, EPI_ISL_621695, EPI_ISL_621697, EPI_ISL_621705, EPI_ISL_621714, EPI_ISL_621715, EPI_ISL_621776, EPI_ISL_621813, EPI_ISL_621833, EPI_ISL_621834, EPI_ISL_621835, EPI_ISL_621836, EPI_ISL_621838, EPI_ISL_621839, EPI_ISL_621840, EPI_ISL_621901, EPI_ISL_621906, EPI_ISL_621907, EPI_ISL_621935, EPI_ISL_621953, EPI_ISL_621977, EPI_ISL_621979, EPI_ISL_622034, EPI_ISL_622042, EPI_ISL_622051, EPI_ISL_622070, EPI_ISL_622089, EPI_ISL_622111, EPI_ISL_622134, EPI_ISL_622139, EPI_ISL_622153, EPI_ISL_622158, EPI_ISL_622159, EPI_ISL_622314, EPI_ISL_622331, EPI_ISL_622704, EPI_ISL_622717, EPI_ISL_622718, EPI_ISL_625733, EPI_ISL_625734, EPI_ISL_625735, EPI_ISL_625736, EPI_ISL_625759, EPI_ISL_625764, EPI_ISL_625765, EPI_ISL_625768, EPI_ISL_625778, EPI_ISL_625780, EPI_ISL_625784, EPI_ISL_625788, EPI_ISL_625789, EPI_ISL_625790, EPI_ISL_625794, EPI_ISL_625795, EPI_ISL_625800, EPI_ISL_625802, EPI_ISL_625803, EPI_ISL_625805, EPI_ISL_625806, EPI_ISL_625809, EPI_ISL_625812, EPI_ISL_625813, EPI_ISL_625814, EPI_ISL_625818, EPI_ISL_625819, EPI_ISL_625823, EPI_ISL_625824, EPI_ISL_625826, EPI_ISL_625827, EPI_ISL_625832, EPI_ISL_625839, EPI_ISL_626161			
see above	Department of Virus and Microbiological Special Diagnostics, Statens Serum Institut, Denmark	Albertsen lab, Department of Chemistry and Bioscience, Aalborg University, Denmark	Danish Covid-19 Genome Consortia
EPI_ISL_775314, EPI_ISL_2333079	Dept. of Medical Microbiology, Stavanger University Hospital, Helse Stavanger HF	Norwegian Institute of Public Health, Department of Virology	'Kathrine Stene-Johansen; Atiya R Ali; Debech Nadia; Engebretsen Serina Beate; Garcia Llorente Ignacio; Hilde Elshaug; Hilde Vollan; Jon Bråte; Kamilla Heddeland Instefjord; Karoline Bragstad; Kathrine Stene-Johansen; Line Victoria Moen; Marie Paulsen Madsen; Olav Hungnes; Pedersen Benedikte Nevjen; Rasmus Riis Kopperud
EPI_ISL_1149094, EPI_ISL_1150663	Diagnosticum - Labor Neukirchen	Robert Koch Institute	
EPI_ISL_2308743	Dipartimento Militare di Medicina Legale di Messina	Virology Laboratory, Scientific Department, Army Medical Center	Anella Monte; Anna Anselmo; Annalisa Fedele; Antonella Fortunato; Basilio Leonardo; Filippo Molinari; Florigio Lista; Francesco Giordani; Giancarlo Petralito; Giandomenico Cerreto; Giulia Campoli; Giuseppe Mario Mento; Giuseppina Russo; Lucia Nicosia; Marzia Cavalli; Mirella D'Anieri.; Riccardo De Sanctis; Rossella Brandi; Santo Sorrenti; Silvia Fillo; Vanessa Vera Fain
EPI_ISL_904308, EPI_ISL_904456, EPI_ISL_2145327, EPI_ISL_2145374, EPI_ISL_2145490	Dutch COVID-19 response team	Erasmus Medical Center	Anne van der Linden; Annemiek van der Eijk; Bas Oude Munnink; Corine GeurtsvanKessel; David Nieuwenhuijse; Emmanuelle Munger; Irina Chestakova; Marion Koopmans; Marjan Boter; Reina Sikkema; Richard Molenkamp; on behalf of the Dutch national COVID-19 respo; on behalf of the Dutch national COVID-19 response team.
EPI_ISL_723197, EPI_ISL_790596, EPI_ISL_824004, EPI_ISL_824005, EPI_ISL_824006, EPI_ISL_824007, EPI_ISL_824008, EPI_ISL_824009, EPI_ISL_824010, EPI_ISL_824011, EPI_ISL_824014, EPI_ISL_824015, EPI_ISL_824016, EPI_ISL_824017, EPI_ISL_824018, EPI_ISL_824019, EPI_ISL_824021, EPI_ISL_905086, EPI_ISL_905093, EPI_ISL_905142, EPI_ISL_905144, EPI_ISL_905145, EPI_ISL_905226, EPI_ISL_905683, EPI_ISL_1013783, EPI_ISL_1014339, EPI_ISL_1035447, EPI_ISL_1035719, EPI_ISL_1165652, EPI_ISL_1232284, EPI_ISL_1232311, EPI_ISL_2094355			
see above	Dutch COVID-19 response team	National Institute for Public Health and the Environment (RIVM)	Adam Meijer; AnneMarie van den Brandt; Annelies Kroneman; Bas van der Veer; Chantal Reusken; Dennis Schmitz; Dirk Eggink; Euníce Then; Florian Zwagemaker; Harry Vennema; James Groot; Jeroen Cremer; Karim Hajji; Kim Freriks; Linda van de Nes; Lisa Wijsman; Lynn Aarts; Melissa van Tuil; Ryanne Jaarsma; Sanne Bos; Sharon van den Brink; Sjoerd Kuiling; on behalf of the national COVID-19 response team
EPI_ISL_1813275	EXCITE Lab	Andersen lab at Scripps Research	Alexandre Bolze; Alice Summerfield; Celena Andrade; Charlotte Rivera-Garcia; David Becker; Efren Sandoval; Elizabeth Cirulli; Francisco Tanudjaja; Geraint Levan; James Lu + SEARCH; Jason Nguyen; Jimmy Ramirez; Kelly Schiabor Barrett; Magnus Isaksson; Marc Laurent; Nicole L Washington; Ryan Cho; Sherry Wang; Simon White; Tyler Cassens; William Lee
EPI_ISL_1846133	Eurofins LifeCodexx GmbH	Robert Koch Institute	
EPI_ISL_2316409	Eurofins MVZ Labor Gelsenkirchen	Robert Koch Institute	
EPI_ISL_602282, EPI_ISL_602284, EPI_ISL_602289, EPI_ISL_602291, EPI_ISL_602298, EPI_ISL_602299, EPI_ISL_602301, EPI_ISL_602302, EPI_ISL_2466382			
see above	Evangelisches Klinikum Bethel, Institut für Laboratoriumsmedizin, Mikrobiologie und Hygiene	Bielefeld University	Alexander Sczyrba; Christiane Scherer; David Brandt; Jörn Kalinowski; Levin-Joe Klages; Marina Simunovic; Markus Haak; Svenja Vinke; Tobias Busche
EPI_ISL_2505417	Fimlab Laboratoriot Oy Tampere	Expert Microbiology, National Institute for Health and Welfare	Carita Savolainen-Kopra; Erika Lindh; Haider al-Hello; Jani Halkilahti; Kirsi Liitsola; Niina Ikonen; Olli Vapalahti; Pekka Ellonen; Phuoc Truong; Päivi Laurila; Ravi Kant; Sari Hannula; Soile Blomqvist; Teemu Smura
EPI_ISL_1118428, EPI_ISL_1118429, EPI_ISL_1118430, EPI_ISL_1118431, EPI_ISL_1118432, EPI_ISL_1192238, EPI_ISL_1192245, EPI_ISL_1192248			
see above	Foerde Hospital, Department of Microbiology	Norwegian Institute of Public Health, Department of Virology	Atiya R Ali; Debech Nadia; Engebretsen Serina Beate; Garcia Llorente Ignacio; Hilde Elshaug; Hilde Vollan; Kamilla Heddeland Instefjord; Karoline Bragstad; Kathrine Stene-Johansen; Marie Paulsen Madsen; Olav Hungnes; Pedersen Benedikte Nevjen; Rasmus Riis Kopperud
EPI_ISL_549063, EPI_ISL_549065, EPI_ISL_549066, EPI_ISL_549073, EPI_ISL_775299, EPI_ISL_775304, EPI_ISL_1118434, EPI_ISL_2333071, EPI_ISL_2333528, EPI_ISL_2333530, EPI_ISL_2333539, EPI_ISL_2426031, EPI_ISL_2426032, EPI_ISL_2426033, EPI_ISL_2491441, EPI_ISL_2491445			
see above	Furst Medical Laboratory	Norwegian Institute of Public Health, Department of Virology	'Kathrine Stene-Johansen; Atiya R Ali; Debech Nadia; Engebretsen Serina Beate; Garcia Llorente Ignacio; Hilde Elshaug; Hilde Synnøve Vollan; Hilde Vollan; Jon Bråte; Kamilla Heddeland Instefjord; Karoline Bragstad; Kathrine Stene-Johansen; Line Victoria Moen; Marie Paulsen Madsen; Olav Hungnes; Pedersen Benedikte Nevjen; Rasmus Riis Kopperud
EPI_ISL_406798	General Hospital of Central Theater Command of People's Liberation Army of China	BGI & Institute of Microbiology, Chinese Academy of Sciences & Shandong First Medical University & Shandong Academy of Medical Sciences & General Hospital of Central Theater Command of People's Liberation Army of China	Weifeng Shi and Zhenhong Hu; Weijun Chen; Yuhai Bi
EPI_ISL_452218, EPI_ISL_452219, EPI_ISL_452220, EPI_ISL_452221, EPI_ISL_452222, EPI_ISL_452223	Goethe University Hospital Frankfurt	Institute for Medical Virology, Goethe University Hospital Frankfurt	Annemarie Berger; Björn Rotter; Denisa Bojkova; Jindrich Cinatl; Klaus Hoffmeier; Sandra Westhaus; Sandra Ciesek; Sebastian Hoehl; Tuna Toptan; and Marek Widera
EPI_ISL_2232206, EPI_ISL_2301845, EPI_ISL_2302011, EPI_ISL_2302075, EPI_ISL_2302090, EPI_ISL_2369018, EPI_ISL_2369039, EPI_ISL_2369075			
see above	Greek Genome Center, Biomedical Research Foundation of the Academy of Athens (BRFAA)	Greek Genome Center, Biomedical Research Foundation of the Academy of Athens (BRFAA)	Dimitrios Thanos; Emmanouil Athanasiadis; Giannis Vatsellas; Katerina Zoi; Theodoros Loupis
EPI_ISL_1085524	Groupe LCD	Department of Virology, Henri Mondor University Hospital, Assistance Publique Hôpitaux de Paris, Université Paris-Est Créteil, INSERM U955	Alexandre Soulier; Christophe Rodriguez; Elisabeth Trawinski; Guillaume Gricourt; Jean-Michel Pawlotsky; Melissa N'Debi; Slim Fourati; Vanessa Demontant
EPI_ISL_2348501, EPI_ISL_2348510, EPI_ISL_2348648, EPI_ISL_2348654, EPI_ISL_2348665, EPI_ISL_2348684, EPI_ISL_2348724, EPI_ISL_2362044			
see above	Gyncentrum	Gyncentrum	Adam Pudelko; Adam Pudełko; Agnieszka Polak; Aleksandra Skubis-Sikora; Celina Kruszniewska-Rajs; Emilia Morawiec; Jolanta Bartosiewicz-Wasik; Jolanta Bartosiewicz- Wąsik; Magdalena Samul; Maria Maklasinska-Majdanik; Maria Maklasińska-Majdanik; Pawel Czerwinski; Paweł Czerwiński; Robert Wojtyczka; Tomasz Wasik; Tomasz Wąsik

EPI_ISL_883158, EPI_ISL_883159, EPI_ISL_883160, EPI_ISL_883161, EPI_ISL_883162, EPI_ISL_883163, EPI_ISL_883164, EPI_ISL_883165, EPI_ISL_883166, EPI_ISL_883167, EPI_ISL_883168, EPI_ISL_883169, EPI_ISL_883170, EPI_ISL_883171			
see above	Hannover Medical School, Institute of Virology	Hannover Medical School, Institute of Virology	Jasper Götting; Lars Steinbrück
EPI_ISL_1088902	Helix / Illumina	Respiratory Viruses Branch, Division of Viral Diseases, Centers for Disease Control and Prevention	Alexandre Bolze; Ary Ascencio; Ben L. Rambo-Martin; Brad Sickler; Charlotte Rivera-Garcia; Christine Tran; Clinton R. Paden; Dakota Howard; David Becker; Dhwani Batra; Duncan MacCannell; Efren Sandoval; Eileen de Feo; Elizabeth Cirulli; Eric Allen; Geraint Levan; James Lu; Jan Antico; Jason Nguyen; Jimmy Ramirez; Jingtao Liu; Kelly Schiabor Barrett; Kim Gietzen; Magnus Isaksson; Marc Laurent; Matthew Tolentino; Nicole L. Washington; Peter W. Cook; Phil Febbo; Ryan Cho; Shannon Wickline; Sherry Wang; Simon White; Summer Galloway; Suxiang Tong; Tyler Cassens; William Lee
EPI_ISL_718237	Hospital	National Reference Center for Viruses of Respiratory Infections, Institut Pasteur, Paris	Angela Brisebarre; Camille Capel; Etienne Simon-Lorière; Gisèle Lagathu; Marion Barbet; Maud Vanpeene; Méline Bizard; Sylvie Behillil; Sylvie van der Werf; Vincent Enouf
EPI_ISL_862581	Hospital Clínic	Instituto de Salud Carlos III	A. Monzón; F. Casas; I. Jiménez; I. Marcos; Iglesias-Caballero; M. Pozo; M. Cuesta; M. González-Esguevillas; M. Zaballos; M.A.; M.Camarero; P. Jiménez; S. Juliá; S. Molinero Calamita; S. Varona
EPI_ISL_1225137	Hospital Clínico Universitario Lozano Blesa de Zaragoza (España)	SeqCOVID-SPAIN consortium/IBV(CSIC)	Jessica Bueno and SeqCOVID-SPAIN consortium; Rafael Benito; Sonia Algarate
EPI_ISL_1423921	Hospital Clínico Universitario Virgen de la Arrixaca (Murcia)	SeqCOVID-SPAIN consortium/IBV(CSIC)	Antonio Moreno Docón; Genoveva Yagüe and SeqCOVID-SPAIN consortium; Manuel Segovia Hernández
EPI_ISL_2401191, EPI_ISL_2401192, EPI_ISL_2401204, EPI_ISL_2401211, EPI_ISL_2401212, EPI_ISL_2401224, EPI_ISL_2401227, EPI_ISL_2401231, EPI_ISL_2401232			
see above	Hospital General Universitario Gregorio Marañón	Hospital General Universitario Gregorio Marañón	Cristina Rodriguez-Grande; Darío García de Viedma; Julia Suárez; Laura Pérez-Lago; Marta Herranz Martin; Patricia Muñoz; Pedro Sola Campoy; Pilar Catalán; Sergio Buenestado Serrano; Victor Manuel de la Cueva
EPI_ISL_779996, EPI_ISL_814058, EPI_ISL_861843	Hospital General Universitario Gregorio Marañón	SeqCOVID-SPAIN consortium/IBV(CSIC)	Darío García de Viedma; Jon Sicilia; Julia Suárez; Laura Pérez-Lago; Marta Herranz; Patricia Muñoz and SeqCOVID-SPAIN consortium; Pedro J Sola-Campoy; Pilar Catalán; Sergio Buenestado-Serrano; Victor Manuel de la Cueva
EPI_ISL_1311888, EPI_ISL_2103493	Hospital General Universitario de Alicante - Instituto de Investigación Sanitaria y Biomédica de Alicante	SeqCOVID-SPAIN consortium/IBV(CSIC)	Carmen Molina Pardines and SeqCOVID-SPAIN consortium; Maripaz Ventero Martín
EPI_ISL_2147373, EPI_ISL_2147392	Hospital General Universitario de Ciudad Real	SeqCOVID-SPAIN consortium/IBV(CSIC)	Cristina Colmenarejo; Jorge Alfredo Pérez García and SeqCOVID-SPAIN consortium; José Martínez-Alarcón; Lidia García-Agudo; Marta Torres-Narbona; Soledad Illescas Fernández-Bermejo
EPI_ISL_1916188	Hospital General Universitario de Elche - Fundación para el Fomento de la Investigación Sanitaria y Biomédica de la Comunitat Valenciana, Servicio de Microbiología	SeqCOVID-SPAIN consortium/IBV(CSIC)	Antonio Galiana Cabrera; Judith Sánchez-Almendro; Montserrat Ruiz García; Nieves Gonzalo Jiménez and SeqCOVID-SPAIN consortium
EPI_ISL_1938921, EPI_ISL_1938922, EPI_ISL_1938938, EPI_ISL_1938941, EPI_ISL_1938947, EPI_ISL_1938948	Hospital Universitari Arnau de Vilanova de Lleida	SeqCOVID-SPAIN consortium/IBV(CSIC)	Alba Bellés Bellés; Albert Bernet Sánchez; Eric López González; Iván Prats Sánchez; Mercè García González and SeqCOVID-SPAIN consortium
EPI_ISL_1360265, EPI_ISL_1963989, EPI_ISL_1963993, EPI_ISL_2017682	Hospital Universitari Bellvitge	Microbiology Department	Aida Gonzalez-Diaz; Carmen Ardanuy; Jordi Camara; Jordi Niubó; Laura Calatayud; M Angeles Domínguez; Miguel Fernandez-Huerta; Sara Marti
EPI_ISL_824413, EPI_ISL_825351, EPI_ISL_1080843, EPI_ISL_1391181, EPI_ISL_2284948, EPI_ISL_2391938, EPI_ISL_2458135, EPI_ISL_2458159, EPI_ISL_2493138			
see above	Hospital Universitari Vall d'Hebron - Vall d'Hebron Institut de Recerca	Hospital Universitari Vall d'Hebron - Vall d'Hebron Institut de Recerca	Alejandra González-Sánchez; Andrés Antón; Ariadna Rando; Carla Castillo; Cristina Andrés; Damir Garcia-Cehic; Josep F Abril; Josep Quer; Juliana Esperalba; Maria Carmen Martin; Maria Gema Codina; Maria Piñana; Tomàs Pumarola
EPI_ISL_2016366, EPI_ISL_2016367	Hospital Universitari de La Plana	SeqCOVID-SPAIN consortium/IBV(CSIC)	Alberto Yagüe Muñoz; Helena Buj Jordá; Juan B. Bellido Blasco; Maria Gil Fortuño and SeqCOVID-SPAIN consortium; Noelia Henández Pérez; Óscar Pérez Olaso
EPI_ISL_2100078, EPI_ISL_2100094, EPI_ISL_2100099, EPI_ISL_2100101, EPI_ISL_2100102	Hospital Universitari i Politècnic La Fe de València	SeqCOVID-SPAIN consortium/IBV(CSIC)	Ana Gil Brusola; Eva González Barberá; José Luis López Hontangas and SeqCOVID-SPAIN consortium; María Dolores Gómez Ruiz; Salvador Giner Almaraz
EPI_ISL_2099969, EPI_ISL_2099991, EPI_ISL_2100032, EPI_ISL_2100037, EPI_ISL_2100059, EPI_ISL_2271234, EPI_ISL_2271237, EPI_ISL_2271385, EPI_ISL_2271387, EPI_ISL_2464635, EPI_ISL_2464639, EPI_ISL_2464643, EPI_ISL_2464644, EPI_ISL_2464645			
see above	Hospital Universitario Clínico San Cecilio	SeqCOVID-SPAIN consortium/IBV(CSIC)	Adolfo de Salazar; Federico García; Laura Viñuela; Natalia Chueca and SeqCOVID-SPAIN consortium
EPI_ISL_1938849	Hospital Universitario Marqués de Valdecilla - IDIVAL (Santander, Cantabria)	SeqCOVID-SPAIN consortium/IBV(CSIC)	Daniel Pablo Marcos; Jesús Rodríguez Rodríguez; Jose Manuel Méndez Legaza; María Eliecer Cano García; María Siller Ruiz and SeqCOVID-SPAIN consortium; Mónica Gozalo Margüello
EPI_ISL_1938709, EPI_ISL_1938745, EPI_ISL_2271326, EPI_ISL_2271333	Hospital Universitario Virgen de las Nieves de Granada-SAS	SeqCOVID-SPAIN consortium/IBV(CSIC)	Irene Pedrosa Corral; José M. Navarro-Marí and SeqCOVID-SPAIN consortium; Sara Sanbonmatsu Gámez
EPI_ISL_1588976, EPI_ISL_1938747, EPI_ISL_1938748, EPI_ISL_1938749, EPI_ISL_1938765, EPI_ISL_1938778, EPI_ISL_1938780, EPI_ISL_1938787			
see above	Hospital Universitario de Guadalajara	SeqCOVID-SPAIN consortium/IBV(CSIC)	Alejandro González Praetorius y Mariela Martínez Ramírez and SeqCOVID-SPAIN consortium
EPI_ISL_2081680, EPI_ISL_2135347, EPI_ISL_2135350, EPI_ISL_2135354, EPI_ISL_2135355, EPI_ISL_2135356, EPI_ISL_2135358, EPI_ISL_2135361, EPI_ISL_2135365, EPI_ISL_2135368, EPI_ISL_2135369			
see above	Hospital Universitario de La Ribera (Alzira, València)	SeqCOVID-SPAIN consortium/IBV(CSIC)	Julia González Cantó and SeqCOVID-SPAIN consortium; Olalla Martínez Macias
EPI_ISL_1671557, EPI_ISL_2099811, EPI_ISL_2099814, EPI_ISL_2099815, EPI_ISL_2099816, EPI_ISL_2099822, EPI_ISL_2099823, EPI_ISL_2099824, EPI_ISL_2099827, EPI_ISL_2099828, EPI_ISL_2099829, EPI_ISL_2099831, EPI_ISL_2099833, EPI_ISL_2099837, EPI_ISL_2099838, EPI_ISL_2099841, EPI_ISL_2099845			
see above	Hospital de la Santa Creu i Sant Pau. Servicio de Microbiología	SeqCOVID-SPAIN consortium/IBV(CSIC)	Elisenda Miró and SeqCOVID-SPAIN consortium; Ferran Navarro; Núria Rabella
EPI_ISL_2333495, EPI_ISL_2333544	Hospital of Southern Norway - Kristiansand, Department of Medical Microbiology	Norwegian Institute of Public Health, Department of Virology	'Kathrine Stene-Johansen; Atiya R Ali; Debech Nadia; Engebretsen Serina Beate; Garcia Llorente Ignacio; Hilde Elshaug; Hilde Vollan; Jon Bråte; Kamilla Heddeland Instefjord; Karoline Bragstad; Line Victoria Moen; Marie Paulsen Madsen; Olav Hungnes; Pedersen Benedikte Nevjen; Rasmus Riis Kopperud
EPI_ISL_912731, EPI_ISL_912809, EPI_ISL_912952	Hôpital Henri Mondor	Department of Virology, Henri Mondor University Hospital, Assistance Publique Hôpitaux de Paris, Université Paris-Est Créteil, INSERM U955	Alexandre Soulier; Christophe Rodriguez; Elisabeth Trawinski; Guillaume Gricourt; Jean-Michel Pawlotsky; Melissa N'Debi; Slim Fourati; Vanessa Demontant
EPI_ISL_2464870, EPI_ISL_2465004	Hôpital Saint Antoine	Department of Virology, Henri Mondor University Hospital, Assistance Publique Hôpitaux de Paris, Université Paris-Est Créteil, INSERM U955	Alexandre Soulier; Christophe Rodriguez; Elisabeth Trawinski; Guillaume Gricourt; Jean-Michel Pawlotsky; Melissa N'Debi; Slim Fourati; Vanessa Demontant
EPI_ISL_2485204, EPI_ISL_2485214, EPI_ISL_2485225	ILV Kärnten	Bergthaler laboratory, CeMM Research Center for Molecular Medicine of the Austrian Academy of Sciences	Andreas Bergthaler; Anna Schedl; Bekir Erguner; Benedikt Agerer; Christoph Bock; Fabian Amman; Jan Laine; Lukas Endler; Maelle Le Moing; Martin Senekowitsch; Michael Schuster; Petr Triska; Thomas Penz
EPI_ISL_1844606	IMD - Labor Oderland	Robert Koch Institute	
EPI_ISL_1048316	IMD - MVZ Labor Greifswald	Robert Koch Institute, Influenza and respiratory viruses FG17 & Bioinformatics MF1, Berlin, Germany	A. Kruggel; Aleksandar Radonic; K. Meinck; Marianne Wedde; Oliver Drechsel; Ralf Duerrwald; Rene Kmiecinski; Stefan Kroeger; Stephan Fuchs; Thorsten Wolff
EPI_ISL_1169886	INMI Lazzaro Spallanzani IRCCS	INMI Lazzaro Spallanzani IRCCS	A Di Caro; B Bartolini; CEM Gruber; E Giombini; F Messina; M Rueca; MR Capobianchi; O Butera
EPI_ISL_1670904, EPI_ISL_1670906, EPI_ISL_1670907, EPI_ISL_1670909	IRCCS San Gallicano Dermatological Institute	IRCCS Regina Elena National Cancer Institute	Aldo Morrone; Fabrizio Ensoli; Francesca De Nicola; Fulvia Pimpinelli; Gennaro Ciliberto; Giovanni Blandino; Grazia Prignano; Matteo Pallocca; Maurizio Fanciulli; Sabrina Strano; Sara Donzelli
EPI_ISL_1390685	IZSM	TIGEM	Andrea Ballabio; Anna Manfredi; Antonio Grimaldi; Antonio Limone Luigi Atripaldi Pellegrino Cerino; Biancamaria Pierri Claudia Tiberio Valentina Bouche; Chiara Colantuono; Davide Cacchiarelli; Denise Di Concilio; Francesco Panariello; Lucio Di Filippo; Marcello Salvi; Maria Concetta Cuomo; Patrizia Annunziata
EPI_ISL_1938976, EPI_ISL_1938979, EPI_ISL_1938985, EPI_ISL_2464664, EPI_ISL_2464668, EPI_ISL_2465728, EPI_ISL_2465740			
see above	IdISSC/Hospital Clínico San Carlos de Madrid	SeqCOVID-SPAIN consortium/IBV(CSIC)	Alberto Delgado-Iribarren; Esther Culebras López; Jorge Matias-Guiu; Luis Ortega Medina; Silvia Sánchez Ramón; Ulises Gómez-Pinedo and SeqCOVID-SPAIN consortium; Vicente Estrada Pérez
EPI_ISL_708039	Innlandet Hospital Trust, Division Lillehammer, Department for Medical Microbiology	Norwegian Institute of Public Health, Department of Virology	Hilde Elshaug; Hilde Vollan; Kamilla Heddeland Instefjord; Karoline Bragstad; Kathrine Stene-Johansen; Marie Paulsen Madsen; Olav Hungnes; Rasmus Riis Kopperud

EPI_ISL_2466045	Inst. for Med. Virology, University Hospital Frankfurt, Goethe University Frankfurt	Inst. for Med. Virology, University Hospital Frankfurt, Goethe University Frankfurt	Goetsch, U.; Gottschalk; H.F.; Pallas, C.; R. and Ciesek, S.; Rabenau; Toptan, T.; Widera, M.; Wilhelm, A.
EPI_ISL_1138634, EPI_ISL_1138665	Institut für Medizinische Virologie, Universitätsklinikum Frankfurt	Institut für Medizinische Virologie, Universitätsklinikum Frankfurt	Barbara Muehlemann et al
EPI_ISL_1008013	Institut für Pathologie, Salzkammergut Klinikum Vöcklabruck	Bergthaler laboratory, CeMM Research Center for Molecular Medicine of the Austrian Academy of Sciences	Andreas Bergthaler; Anna Schedl; Bekir Erguner; Benedikt Agerer; Christoph Bock; Jan Laine; Lukas Endler; Maelle Le Moing; Martin Senekowitsch; Michael Schuster; Thomas Penz
EPI_ISL_437919, EPI_ISL_437931, EPI_ISL_437932, EPI_ISL_934547, EPI_ISL_1671133, EPI_ISL_1671134	Institut für Virologie am Department für Hygiene, Mikrobiologie und Public Health	Bergthaler laboratory, CeMM Research Center for Molecular Medicine of the Austrian Academy of Sciences	Alexander Lercher; Alexandra Popa; Andreas Bergthaler; Anna Schedl; Bekir Erguner; Benedikt Agerer; Christoph Bock; Dorothee von Laer; Elisabeth Puchhammer-Stoeckl; Fabian Amman; Guenter Weiss; Henrique Colaco; Jakob-Wendelin Genger; Jan Laine; Judith Aberle; Lukas Endler; Maelle Le Moing; Manfred Nairz; Mark Smyth; Martin Senekowitsch; Michael Schuster; Petr Triska; Stephan Aberle; Thomas Penz; Wegene Borena
EPI_ISL_485810, EPI_ISL_485811, EPI_ISL_485813, EPI_ISL_508687, EPI_ISL_508688, EPI_ISL_508689, EPI_ISL_508690, EPI_ISL_508691, EPI_ISL_508694, EPI_ISL_508695, EPI_ISL_508696, EPI_ISL_508699, EPI_ISL_508701, EPI_ISL_508702			
see above	Institut für Virologie und Epidemiologie der Viruskrankheiten, Universitätsklinikum Tübingen	NGS Competence Center Tübingen, Institut für Medizinische Mikrobiologie und Hygiene, Universitätsklinikum Tübingen	Angel Angelov; Angelov et al.
EPI_ISL_572330, EPI_ISL_572331	Institute for Virology, University Hospital Duesseldorf, Medical Faculty, Heinrich-Heine-University Duesseldorf	Institute for Virology, University Hospital Duesseldorf, Medical Faculty, Heinrich-Heine-University Duesseldorf	; Alexander Killer; Andreas Walker; Annemarie Mohring; Anselm Kunstein; Ansgar Schulz; Björn Jensen; Caroline Klindt; Edwin Bölke; Gerald Antoch; Heiner Schaal; Jennifer Neubert; Johannes Bode; Johannes C. Fischer; Jörg Timm; Lisa Müller; Maximilian Damagnez; Nadine Lübke; Ortwin Adams; Philipp Albrecht; Philipp Ostermann; Saskia Elben; Tina Senff; Tom Lüdde; Torsten Feldt; Verena Keitel
EPI_ISL_602469, EPI_ISL_602470, EPI_ISL_602471, EPI_ISL_602479, EPI_ISL_602482, EPI_ISL_602485, EPI_ISL_602496, EPI_ISL_602497, EPI_ISL_602502, EPI_ISL_602503, EPI_ISL_626215, EPI_ISL_626220, EPI_ISL_626221, EPI_ISL_626222, EPI_ISL_626229			
see above	Institute for Virology, University Hospital Essen	Center of Medical Microbiology, Virology, and Hospital Hygiene, University of Duesseldorf	Alexander Dilthey; Andreas Walker; Daniel Strelow; Jessica Nicolai; Jörg Timm; Klaus Pfeffer; Lisanna Hülse; Malte Kohns Vasconcelos; Maximilian Damagnez; Nadine Lübke; Olympia E. Anastasiou; Tobias Wienemann; Torsten Houwaart; Ulf Dittmer
EPI_ISL_886157, EPI_ISL_887107, EPI_ISL_887108, EPI_ISL_887109, EPI_ISL_887110, EPI_ISL_887111, EPI_ISL_887112, EPI_ISL_887113, EPI_ISL_887114			
see above	Institute of Medical Microbiology and Virology, University Hospital Carl Gustav Carus, TU Dresden	DRESDEN-concept Genome Center, CMCB, TU Dresden	Beil J.; Brown T.; Büttner L.; Gscheidel N.; Hochauf-Stange K.; Klemroth S.; Lindemann D.; Mehnert G.; Petzold A.; Reinhardt S.; Rost F.; Sameith K.; Winkler S.
EPI_ISL_1964507	Institute of Microbiology and Immunology, Faculty of Medicine, University of Ljubljana	Institute of Microbiology and Immunology, Faculty of Medicine, University of Ljubljana	Alen Suljič; Andraž Celar; Dominika Šturm; Doroteja Vlaj; Mario Poljak; Matic Brvar; Miša Korva; Patricija Pozvek; Samo Zakotnik; Tatjana Avšič – Županc; Tomaž Mark Zorec; Špela Pleh
EPI_ISL_402123	Institute of Pathogen Biology, Chinese Academy of Medical Sciences & Peking Union Medical College	Institute of Pathogen Biology, Chinese Academy of Medical Sciences & Peking Union Medical College	Chao Wu; Jianwei Wang; Lili Ren; Qi Jin; Yiwei Liu; Zhiqiang Wu; Zichun Xiang
EPI_ISL_852636, EPI_ISL_852638, EPI_ISL_852640, EPI_ISL_852644, EPI_ISL_852646, EPI_ISL_852650, EPI_ISL_852651, EPI_ISL_852652, EPI_ISL_852653, EPI_ISL_852654, EPI_ISL_852655, EPI_ISL_852656, EPI_ISL_852657, EPI_ISL_852658, EPI_ISL_852661, EPI_ISL_852663, EPI_ISL_852673, EPI_ISL_852687, EPI_ISL_852688, EPI_ISL_852690, EPI_ISL_852703, EPI_ISL_852705, EPI_ISL_852706, EPI_ISL_852721, EPI_ISL_852727, EPI_ISL_852731, EPI_ISL_852750, EPI_ISL_852754, EPI_ISL_852760, EPI_ISL_852763, EPI_ISL_852768, EPI_ISL_852771, EPI_ISL_852783, EPI_ISL_852793, EPI_ISL_852795, EPI_ISL_852799, EPI_ISL_852805, EPI_ISL_852806, EPI_ISL_852807, EPI_ISL_852809, EPI_ISL_893757, EPI_ISL_893769, EPI_ISL_918347, EPI_ISL_918355, EPI_ISL_1195352, EPI_ISL_1195355, EPI_ISL_1195357, EPI_ISL_1195362, EPI_ISL_1195365, EPI_ISL_1195403, EPI_ISL_1195405, EPI_ISL_1195406, EPI_ISL_1357673, EPI_ISL_1588642, EPI_ISL_1588655, EPI_ISL_1588662, EPI_ISL_1588670, EPI_ISL_2535426			
see above	Institute of Virology, Medical Center, University of Freiburg, Freiburg, Germany	Institute of Virology, Clinial Virus Genomics, Medical Center, University of Freiburg, Freiburg, Germany	Hajo Grundmann; Jonas Fuchs; Lena Jaki; Lisa Kern; Marcus Panning; Sandra Reuter
EPI_ISL_983503, EPI_ISL_983514, EPI_ISL_983523, EPI_ISL_983525, EPI_ISL_983537, EPI_ISL_983554, EPI_ISL_983566, EPI_ISL_983571, EPI_ISL_983582, EPI_ISL_983587			
see above	Institute of Virology, University Hospital, University of Bonn and German Center for Infection Research (DZIF), Bonn-Cologne, Bonn, Germany	Institute of Virology, University Hospital, University of Bonn and German Center for Infection Research (DZIF), Bonn-Cologne, Bonn, Germany	Marek Korencak et al
EPI_ISL_796028, EPI_ISL_796034, EPI_ISL_796043, EPI_ISL_796044	Institute of Virology, University of Cologne	Institute of Virology, University of Cologne	Alex Thielen; Alexander Dilthey; Andreas Walker; Dominik Aschenmeier; Elena Knops; Eva Heger; Gibran Rubio; Jörg Timm; Martin Däumer; Maximilian Damagnez; Rolf Kaiser; Saleta Sierra; Zevanya Tessalonica
EPI_ISL_732019, EPI_ISL_861579	Instituto Nacional de Saude (INSA)	Instituto Nacional de Saude (INSA)	Borges et al
EPI_ISL_1854421, EPI_ISL_1854424	Instituto Nacional de Saude (INSA) and Centro de Investigaçao em Biodiversidade e Recursos Geneticos (CIBIO), Universidade do Porto	Instituto Nacional de Saude (INSA) and Centro de Investigaçao em Biodiversidade e Recursos Geneticos (CIBIO), Universidade do Porto	Borges et al
EPI_ISL_2004304, EPI_ISL_2004305	Instituto Nacional de Saude (INSA) and i3S - Instituto de Investigação e Inovação em Saúde	Instituto Nacional de Saude (INSA)	Borges et al
EPI_ISL_1322217	IrsiCaixa	IrsiCaixa	Bonaventura Clotet; Bonaventura Clotet Gloria Trujillo; Carolina Gonzalez Fernandez; Eulalia Grau; Francesc Catala-Moll; Jaume Trape Pujol; Marc Noguera-Julian; Maria Casadellà; Mariona Parera; Pilar Armengol; Rafel Perez Vidal; Roger Paredes
EPI_ISL_1254970, EPI_ISL_2465043, EPI_ISL_2465052, EPI_ISL_2465056, EPI_ISL_2465066, EPI_ISL_2465100	Istituto Zooprofilattico Sperimentale del Mezzogiorno	Telethon Institute of Genetics and Medicine (TIGEM)	Antonio Grimaldi Patrizia Annunziata Francesco Panariello Biancamaria Pierri Claudia Tiberio Valentina Bouche Chiara Colantuono Maria Concetta Cuomo Denise Di Concilio Lucio Di Filippo Anna Manfredi Marcello Salvi Antonio Limone Luigi Atripaldi Pellegrino Cerino Andrea Ballabio Davide Cacchiarelli; Antonio Grimaldi Patrizia Annunziata Francesco Panariello Biancamaria Pierri Claudia Tiberio Teresa Giuliano Valentina Bouche Chiara Colantuono Maria Concetta Cuomo Denise Di Concilio Lucio Di Filippo Anna Manfredi Marcello Salvi Antonio Limone Luigi Atripaldi Pellegrino Cerino Andrea Ballabio Davide Cacchiarelli
EPI_ISL_1627357	Istituto Zooprofilattico Sperimentale della Puglia e della Basilicata	Istituto Zooprofilattico Sperimentale della Puglia e della Basilicata	Bianco A.; Capozzi L.; Del Sambro L.; Galante D.; Pace L.; Parisi A.; Simone D.
EPI_ISL_949019, EPI_ISL_949022	Jessa	Jessa	Jessa_cmdLab
EPI_ISL_912426, EPI_ISL_1049113, EPI_ISL_2404608	KU Leuven, Rega Institute, Clinical and Epidemiological Virology	KU Leuven, Rega Institute, Clinical and Epidemiological Virology	Bert Vanmechelen; Joan Marti-Carerras; Piet Maes; Tony Wawina-Bokalanga
EPI_ISL_1008002	Klinikum Wels-Grieskirchen	Bergthaler laboratory, CeMM Research Center for Molecular Medicine of the Austrian Academy of Sciences	Andreas Bergthaler; Anna Schedl; Bekir Erguner; Benedikt Agerer; Christoph Bock; Jan Laine; Lukas Endler; Maelle Le Moing; Martin Senekowitsch; Michael Schuster; Thomas Penz
EPI_ISL_1168141	Klinisk mikrobiologi	The Public Health Agency of Sweden	Anna Risberg; Anna-Malin Linde; Carlo Berg; Karin Tegmark-Wisell; Maria Lind Karlberg; Mattias Haukland; Mia Brytting; Noura Walai; Oskar Karlsson Lindsjo; Petra Edquist; Petra Holmstrom; Reza Advani; Sofia Stamouli
EPI_ISL_1150225, EPI_ISL_1847472	LabKom - Labor Augsburg MVZ GmbH	Robert Koch Institute	
EPI_ISL_1211099	LabKom - Labor Mainz MVZ GmbH	Robert Koch Institute	
EPI_ISL_860874, EPI_ISL_860885	Labo Analyses Med	National Reference Center for Viruses of Respiratory Infections, Institut Pasteur, Paris	Angela Brisebarre; Camille Capel; Etienne Simon-Lorière; Lefaure Brieuc; Marion Barbet; Maud Vanpeene; Méline Bizard; Sylvie Behillil; Sylvie van der Werf; Vincent Enouf
EPI_ISL_1843681	Labor 28 MVZ GmbH	Robert Koch Institute	
EPI_ISL_1982602, EPI_ISL_1988174, EPI_ISL_1988175, EPI_ISL_1988203, EPI_ISL_1988225, EPI_ISL_1989460, EPI_ISL_1989665			
see above	Labor Berlin Charité Vivantes GmbH / Institut für Virologie	Charité Universitätsmedizin Berlin, Institut für Virologie/Labor Berlin	Barbara Mühlemann; Christian Drosten; Christine Stephan; Peter Menzel; Rolf Schwarzer; Terry Jones; Victor M Corman
EPI_ISL_1144324	Labor Blackholm MVZ	Robert Koch Institute	
EPI_ISL_1861845, EPI_ISL_1861941, EPI_ISL_1861996, EPI_ISL_1862183, EPI_ISL_2384489	Labor Dr. Fenner und Kollegen	Heinrich Pette Institute, Leibniz Institute for Experimental Virology	Adam Grundhoff; Alexis Robitaille; Johannes Knobloch; Martin Aepfelbacher; Nicole Fischer; Thomas Günther

EPI_ISL_1844609	Labor Dr. Heidrich & Kollegen MVZ GmbH Hamburg	Robert Koch Institute	
EPI_ISL_1847412	Labor Dr. Spranger	Robert Koch Institute	
EPI_ISL_831384	Labor Krone	Robert Koch Institute, Influenza and respiratory viruses FG17 & Bioinformatics MF1, Berlin, Germany	Aleksandar Radonic; Dr. Münstermann. Prof. Tiemann; Marianne Wedde; Oliver Drechsel; Ralf Duerrwald; Rene Kmiecinski; Stefan Kroeger; Stephan Fuchs; Thorsten Wolff
EPI_ISL_1143558	Labor ZOTZ|KLIMAS; MVZ Düsseldorf-Centrum	Robert Koch Institute	
EPI_ISL_1357503	Labor für medizinische Mikrobiologie Bakteriologie Mykologie Virologie & Infektionsserologie	Robert Koch Institute	
EPI_ISL_2388431	Laboratoire de santé publique du Québec	Laboratoire de santé publique du Québec	Guillaume Bourque; Ioannis Ragoussis; Jesse Shapiro; Mark Lathrop and Michel Roger on behalf of the CoVSeQ research group; Sandrine Moreira
EPI_ISL_2401006	Laboratoire national de sante, Microbiology, Virology	Laboratoire national de sante, Microbiology, Microbial Genomics Platform	Anke Wienecke-Baldacchino; Catherine Ragimbeau; Fatu Djabi; Jessica Tapp; Lise Pignon; Raoul Salmon; Tamir Abdelrahman; Trung Nguyen Nguyen
EPI_ISL_739887, EPI_ISL_739997, EPI_ISL_744301	Laboratoire national de santé, Microbiology, Virology	Laboratoire national de santé, Microbiology, Microbial Genomics Platform	Anke Wienecke-Baldacchino; Catherine Ragimbeau; Fatu Djabi; Jessica Tapp; Lise Pignon; Raoul Salmon; Tamir Abdelrahman
EPI_ISL_2434104, EPI_ISL_2434105, EPI_ISL_2434107	Laboratorio Biologia molecolare dell'Istituto di Medicina Aerospaziale di Roma	Virology Laboratory, Scientific Department, Army Medical Center	Anella Monte; Anna Anselmo; Antonella Fortunato; Carmelo Campanella; Carmen Nigro; Filippo Molinari; Florigio Lista; Francesco Giordani; Giancarlo Petralito; Giandomenico Cerreto; Giulia Campoli; Lucia Nicosia; Maria Gravina; Marzia Cavalli; Mattia Rencricca; Mirko Tavernese; Raffaele Cresta; Riccardo De Sanctis; Rossella Brandi; Sara Felici.; Silvia Fillo; Tania Pistoni; Vanessa Vera Fain
EPI_ISL_1916134	Laboratorio de Microbiología. Hospital General Universitario de Elda, Alicante	SeqCOVID-SPAIN consortium/IBV(CSIC)	Cristina Torregrosa Hetland; Eva Pastor Boix; Mª Isabel Gascón Ros; Paloma Cascales Ramos; Pedro Garcinuño Enríquez; Salvador Raga Borja and SeqCOVID-SPAIN consortium
EPI_ISL_2308741	Laboratorio di Diagnostica Molecolare	Virology Laboratory, Scientific Department, Army Medical Center	Anella Monte; Anna Anselmo; Antonella Fortunato; Federica Nuccio; Filippo Molinari; Florigio Lista; Francesco Colaci; Francesco Giordani; Giancarlo Petralito; Giandomenico Cerreto; Manuel Francesca; Mario Tucciarone.; Riccardo De Sanctis; Silvia Fillo; Vanessa Vera Fain
EPI_ISL_882961	Laboratorium Diagnostyi Mikrobiologicznej z Pracownią Prątka Gruźlicy SPSzW im. Jana Bożego w Lublinie	National Institute of Public Health - National Institute of Hygiene	Gawor Jan; Wołkowicz Tomasz; Zacharczuk Katarzyna
EPI_ISL_2246946	Laboratorium Mikrobiologiczne Dolnośląskie Centrum Transplantacji Komórkowych z Krajowym Bankiem Dawców Szpiku	1. Academic Center for Pathomorphological and Genetic-Molecular Diagnostics ltd, Bialystok, Poland 2. National Institute of Public Health - National Institute of Hygiene, Warsaw, Poland	Anetta Sulewska; Jacek Nikliński; Janusz Dzięcioł; Joanna Kiśluk; Katarzyna Zacharczuk; Konrad Raczkowski; Magdalena Nowakowska; Małgorzata Sadkowska-Todys; Piotr Karabowicz; Piotr Majewski; Przemysław Biecek. Joanna Reszeć; Radosław Charkiewicz; Tomasz Wołkowicz
EPI_ISL_1500439, EPI_ISL_1818939	Laboratory of Clinical Microbiology, Virology and Bioemergencies, ASST Fatebenefratelli Sacco - Sacco Hospital	Laboratory of Clinical Microbiology, Virology and Bioemergencies, ASST Fatebenefratelli Sacco - Sacco Hospital	Alberto Rizzo; Alessandro Mancon; Fiorenza Bracchitta; Luca Rizzuto; Maria Rita Gismondo; Valeria Micheli
EPI_ISL_2301674, EPI_ISL_2301675	Laboratory of Clinical Virology	Greek Genome Center, Biomedical Research Foundation of the Academy of Athens (BRFAA)	Dimitrios Thanos; Emmanouil Athanasiadis; George Sourvinos; Giannis Vatsellas; Katerina Zoi; Theodoros Loupis
EPI_ISL_1034858, EPI_ISL_1279857, EPI_ISL_1279874	Landesamt für Verbraucherschutz Sachsen Anhalt, Magdeburg	Institute of Medical Microbiology and Hospital Hygiene	Aljoscha Tersteegen; Prof. Dr. Achim Kaasch
EPI_ISL_407079	Lapland Central Hospital	Department of Virology, University of Helsinki and Helsinki University Hospital, Helsinki, Finland	Hannimari Kallio-Kokko; Olli Vapalahti; Suvi Kuivanen; Teemu Smura
EPI_ISL_645618, EPI_ISL_645985, EPI_ISL_646639, EPI_ISL_646776, EPI_ISL_655682, EPI_ISL_655755, EPI_ISL_655965, EPI_ISL_662062, EPI_ISL_662106, EPI_ISL_675159, EPI_ISL_675574, EPI_ISL_703171, EPI_ISL_703199, EPI_ISL_721323, EPI_ISL_721454, EPI_ISL_762847, EPI_ISL_767307, EPI_ISL_796810, EPI_ISL_821052, EPI_ISL_821229, EPI_ISL_834821, EPI_ISL_834998, EPI_ISL_858414, EPI_ISL_858677, EPI_ISL_878925, EPI_ISL_885424, EPI_ISL_907766, EPI_ISL_915600, EPI_ISL_915620, EPI_ISL_988710, EPI_ISL_991326, EPI_ISL_992474, EPI_ISL_1084679			
see above	Lighthouse Lab in Alderley Park	Wellcome Sanger Institute for the COVID-19 Genomics UK (COG-UK) Consortium	Cordelia Langford; David K. Jackson; Dominic Kwiatkowski; Ewan Harrison; Ian Johnston; Jacquelyn Wynn; Jeffrey Barrett; John Sillitoe on behalf of the Wellcome Sanger Institute COVID-19 Surveillance Team; John Sillitoe on behalf of the Wellcome Sanger Institute COVID-19 Surveillance Team (http://www.sanger.ac.uk/covid-team); Mairead Hyland; Roberto Amato; Sonia Goncalves; The Lighthouse Lab in Alderley Park and Alex Alderton
EPI_ISL_566399, EPI_ISL_580436, EPI_ISL_580600, EPI_ISL_586666, EPI_ISL_586741, EPI_ISL_587746, EPI_ISL_587966, EPI_ISL_598072, EPI_ISL_598247, EPI_ISL_606083, EPI_ISL_607693, EPI_ISL_607770, EPI_ISL_607802, EPI_ISL_608231, EPI_ISL_609004, EPI_ISL_610997, EPI_ISL_611160, EPI_ISL_624468, EPI_ISL_624489, EPI_ISL_624554, EPI_ISL_624667, EPI_ISL_624769, EPI_ISL_625107, EPI_ISL_625254, EPI_ISL_630450, EPI_ISL_630519, EPI_ISL_630640, EPI_ISL_630756			
see above	Lighthouse Lab in Alderley Park	Wellcome Sanger Institute for the COVID-19 Genomics UK (COG-UK) consortium	Cordelia Langford; David K. Jackson; Dominic Kwiatkowski; Ewan Harrison; Ian Johnston; Jacquelyn Wynn; John Sillitoe on behalf of the Wellcome Sanger Institute COVID-19 Surveillance Team; John Sillitoe on behalf of the Wellcome Sanger Institute COVID-19 Surveillance Team (http://www.sanger.ac.uk/covid-team); Mairead Hyland; Roberto Amato; Sonia Goncalves; The Lighthouse Lab in Alderley Park and Alex Alderton
EPI_ISL_643499, EPI_ISL_643664, EPI_ISL_643668, EPI_ISL_643730, EPI_ISL_643767, EPI_ISL_643782, EPI_ISL_645509, EPI_ISL_645897, EPI_ISL_647716, EPI_ISL_656718, EPI_ISL_656817, EPI_ISL_659153, EPI_ISL_659389, EPI_ISL_672919, EPI_ISL_673023, EPI_ISL_705144, EPI_ISL_709877, EPI_ISL_719019, EPI_ISL_719143, EPI_ISL_756680, EPI_ISL_769041, EPI_ISL_777869, EPI_ISL_782920, EPI_ISL_800390, EPI_ISL_800410, EPI_ISL_994517			
see above	Lighthouse Lab in Cambridge	Wellcome Sanger Institute for the COVID-19 Genomics UK (COG-UK) Consortium	Cordelia Langford; David K. Jackson; Dominic Kwiatkowski; Ewan Harrison; Ian Johnston; John Sillitoe on behalf of the Wellcome Sanger Institute COVID-19 Surveillance Team; Rob Howes; Roberto Amato; Sonia Goncalves; The Lighthouse Lab in Cambridge and Alex Alderton
EPI_ISL_581396, EPI_ISL_596952, EPI_ISL_597060, EPI_ISL_597237, EPI_ISL_597295, EPI_ISL_597437, EPI_ISL_601571, EPI_ISL_608412, EPI_ISL_608446, EPI_ISL_608501, EPI_ISL_608536, EPI_ISL_608608, EPI_ISL_608947, EPI_ISL_608969, EPI_ISL_609023, EPI_ISL_609114, EPI_ISL_609636, EPI_ISL_609659, EPI_ISL_609753, EPI_ISL_610641, EPI_ISL_610705, EPI_ISL_610778, EPI_ISL_610792, EPI_ISL_623386, EPI_ISL_623537, EPI_ISL_623643, EPI_ISL_631142, EPI_ISL_631177, EPI_ISL_633147, EPI_ISL_633214, EPI_ISL_633357, EPI_ISL_634802			
see above	Lighthouse Lab in Cambridge	Wellcome Sanger Institute for the COVID-19 Genomics UK (COG-UK) consortium	Cordelia Langford; David K. Jackson; Dominic Kwiatkowski; Ewan Harrison; Ian Johnston; John Sillitoe on behalf of the Wellcome Sanger Institute COVID-19 Surveillance Team; John Sillitoe on behalf of the Wellcome Sanger Institute COVID-19 Surveillance Team (http://www.sanger.ac.uk/covid-team); Rob Howes; Roberto Amato; Sonia Goncalves; The Lighthouse Lab in Cambridge and Alex Alderton
EPI_ISL_641792, EPI_ISL_642284, EPI_ISL_642708, EPI_ISL_643121, EPI_ISL_643140, EPI_ISL_643251, EPI_ISL_643299, EPI_ISL_646496, EPI_ISL_647895, EPI_ISL_647944, EPI_ISL_655864, EPI_ISL_657129, EPI_ISL_661428, EPI_ISL_661613, EPI_ISL_661766, EPI_ISL_661910, EPI_ISL_662449, EPI_ISL_662957, EPI_ISL_719929, EPI_ISL_721000, EPI_ISL_721030, EPI_ISL_721078, EPI_ISL_736695, EPI_ISL_782640, EPI_ISL_820507			
see above	Lighthouse Lab in Glasgow	Wellcome Sanger Institute for the COVID-19 Genomics UK (COG-UK) Consortium	Anna Dominiczak and Alex Alderton; Carol Clugston; Cordelia Langford; David Gray; David K. Jackson; Dominic Kwiatkowski; Ewan Harrison; Harper VanSteenhouse; Ian Johnston; John Sillitoe on behalf of the Wellcome Sanger Institute COVID-19 Surveillance Team; Roberto Amato; Sonia Goncalves; Yumi Kasai
EPI_ISL_530825, EPI_ISL_530866, EPI_ISL_536932, EPI_ISL_540002, EPI_ISL_540089, EPI_ISL_540331, EPI_ISL_540347, EPI_ISL_549610, EPI_ISL_549649, EPI_ISL_549753, EPI_ISL_567047, EPI_ISL_567095, EPI_ISL_567148, EPI_ISL_567300, EPI_ISL_568229, EPI_ISL_568269, EPI_ISL_568340, EPI_ISL_568343, EPI_ISL_568446, EPI_ISL_568464, EPI_ISL_575381, EPI_ISL_579881, EPI_ISL_581068, EPI_ISL_587431, EPI_ISL_587578, EPI_ISL_587695, EPI_ISL_588145, EPI_ISL_588204, EPI_ISL_588841, EPI_ISL_588854, EPI_ISL_588969, EPI_ISL_590577, EPI_ISL_590606, EPI_ISL_590624, EPI_ISL_598917, EPI_ISL_598946, EPI_ISL_599163, EPI_ISL_599173, EPI_ISL_599227, EPI_ISL_599251, EPI_ISL_599298, EPI_ISL_599445, EPI_ISL_599450, EPI_ISL_599489, EPI_ISL_599492, EPI_ISL_599537, EPI_ISL_599691, EPI_ISL_599807, EPI_ISL_600171, EPI_ISL_600224, EPI_ISL_600333, EPI_ISL_600485, EPI_ISL_600509, EPI_ISL_600551, EPI_ISL_600615, EPI_ISL_600629, EPI_ISL_600633, EPI_ISL_600653, EPI_ISL_600678, EPI_ISL_600680, EPI_ISL_600689, EPI_ISL_600752, EPI_ISL_600756, EPI_ISL_600939, EPI_ISL_601024, EPI_ISL_601050, EPI_ISL_601056, EPI_ISL_601074, EPI_ISL_601180, EPI_ISL_601229, EPI_ISL_601233, EPI_ISL_601332, EPI_ISL_625379, EPI_ISL_629760, EPI_ISL_629968, EPI_ISL_633508, EPI_ISL_633665, EPI_ISL_634714			
see above	Lighthouse Lab in Glasgow	Wellcome Sanger Institute for the COVID-19 Genomics UK (COG-UK) consortium	Anna Dominiczak and Alex Alderton; Carol Clugston; Cordelia Langford; David Gray; David K. Jackson; Dominic Kwiatkowski; Ewan Harrison; Harper VanSteenhouse; Ian Johnston; John Sillitoe; John Sillitoe on behalf of the Wellcome Sanger Institute COVID-19 Surveillance Team; John Sillitoe on behalf of the Wellcome Sanger Institute COVID-19 Surveillance Team (http://www.sanger.ac.uk/covid-team); Roberto Amato; Sonia Goncalves; Yumi Kasai
EPI_ISL_540824, EPI_ISL_585378	Lighthouse Lab in Glasgow / MRC-University of Glasgow Centre for Virus Research	COVID-19 Genomics UK (COG-UK) Consortium	Alasdair MacLean; Ana da Silva Filipe; Anna Dominiczak; Antonia Ho; Carol Clugston; Daniel Mair; David Gray; David L Robertson; Elihu Aranday-Cortes; Emma Thomson; Harper VanSteenhouse; James Shepherd; Jenna Nichols; Joseph Hughes; Kathy Li; Kathy Smollett; Kyriaki Nomikou; Lily Tong; Marc Niebel; Matthew Holden; Natasha Jesudason; Natasha Johnson; Patawee Asamaphan; Rajiv Shah; Richard Orton; Rory Gunson; Sarah McDonald; Sharif Shaaban; Sreenu Vattipally; Stephen Carmichael; Yumi Kasai
EPI_ISL_642781, EPI_ISL_642782, EPI_ISL_642858, EPI_ISL_642902, EPI_ISL_643022, EPI_ISL_643034, EPI_ISL_649205, EPI_ISL_649479, EPI_ISL_657627, EPI_ISL_658862, EPI_ISL_659063, EPI_ISL_659096, EPI_ISL_675550, EPI_ISL_709674, EPI_ISL_760943, EPI_ISL_762452, EPI_ISL_783115, EPI_ISL_783328, EPI_ISL_798961, EPI_ISL_864278, EPI_ISL_945239			
see above	Lighthouse Lab in Milton Keynes	Wellcome Sanger Institute for the COVID-19 Genomics UK (COG-UK) Consortium	Cordelia Langford; David K. Jackson; Dominic Kwiatkowski; Ewan Harrison; Ian Johnston; John Sillitoe on behalf of the Wellcome Sanger Institute COVID-19 Surveillance Team; John Sillitoe on behalf of the Wellcome Sanger Institute COVID-19 Surveillance Team (http://www.sanger.ac.uk/covid-team); Roberto Amato; Sonia Goncalves; The Lighthouse Lab in Milton Keynes and Alex Alderton
EPI_ISL_550549, EPI_ISL_550856, EPI_ISL_550916, EPI_ISL_550958, EPI_ISL_551665, EPI_ISL_551678, EPI_ISL_551729, EPI_ISL_551734, EPI_ISL_551995, EPI_ISL_552135, EPI_ISL_552158, EPI_ISL_552229, EPI_ISL_552440, EPI_ISL_552518, EPI_ISL_552529, EPI_ISL_552715, EPI_ISL_553047, EPI_ISL_553111, EPI_ISL_566512, EPI_ISL_566578, EPI_ISL_566594, EPI_ISL_566649, EPI_ISL_566688, EPI_ISL_566911, EPI_ISL_575781, EPI_ISL_575984, EPI_ISL_580639, EPI_ISL_581145, EPI_ISL_581283, EPI_ISL_589266, EPI_ISL_598275, EPI_ISL_598403, EPI_ISL_598415, EPI_ISL_598439, EPI_ISL_598690, EPI_ISL_598757, EPI_ISL_598818, EPI_ISL_599840, EPI_ISL_599887, EPI_ISL_600020, EPI_ISL_600038, EPI_ISL_600089, EPI_ISL_600102, EPI_ISL_600113, EPI_ISL_601340, EPI_ISL_601500, EPI_ISL_601524, EPI_ISL_601562, EPI_ISL_601576, EPI_ISL_601653, EPI_ISL_601759, EPI_ISL_606305, EPI_ISL_606555, EPI_ISL_606596, EPI_ISL_606683, EPI_ISL_606780, EPI_ISL_606808, EPI_ISL_606837, EPI_ISL_608705, EPI_ISL_608780, EPI_ISL_623396, EPI_ISL_623465, EPI_ISL_623535, EPI_ISL_623820, EPI_ISL_629124, EPI_ISL_629132, EPI_ISL_629346, EPI_ISL_629364, EPI_ISL_629365, EPI_ISL_629374, EPI_ISL_629612, EPI_ISL_629647, EPI_ISL_629675, EPI_ISL_630047, EPI_ISL_630060, EPI_ISL_630171, EPI_ISL_630262, EPI_ISL_634449			
see above	Lighthouse Lab in Milton Keynes	Wellcome Sanger Institute for the COVID-19 Genomics UK (COG-UK) consortium	Cordelia Langford; David K. Jackson; Dominic Kwiatkowski; Ewan Harrison; Ian Johnston; John Sillitoe on behalf of the Wellcome Sanger Institute COVID-19 Surveillance Team; John Sillitoe on behalf of the Wellcome Sanger Institute COVID-19 Surveillance Team (http://www.sanger.ac.uk/covid-team); Roberto Amato; Sonia Goncalves; The Lighthouse Lab in Milton Keynes and Alex Alderton
EPI_ISL_1142171	Limbach - MVZ Labor Dr. Volkmann & Kollegen	Robert Koch Institute	
EPI_ISL_838511	Liverpool Clinical Laboratories	COVID-19 Genomics UK (COG-UK)	A Alrezaihi; Alessandro Gerada; Alistair Darby; Angela Cowell; Anita Lucaci; Anu Chawla; Cassie Olateju; Catherine Hartley; Charlotte Nelson;

		Consortium	Ecaterina Vamos; Elaine O'Toole; Eleanor G Bentley; Ghada T Shawli; Isabel García-Dorival; James Johnson; James P Stewart; Jenifer Manson; Joanne Watts; Jones Benjamin; Jordan J Clark; Julian Hiscox; L Luu; Lucille Rainbow; M Almsaud; Margaret Hughes; Mark Whitehead; Matthew Gemmell; Miren Iturriza-Gomara; Muhannad Alruwaili; N.P Randle; Neil Swainston; PKF Gilmore; Parul Sharma; Rebekah Penrice-Randal; Richard Eccles; Richard Gregory; Sam Haldenby; Steve Paterson; Stuart D Armstrong; Trevor Ian Robinson; Ximeng Han
EPI_ISL_1846223	MDI Limbach Berlin GmbH; MVZ Labor Berlin	Robert Koch Institute	
EPI_ISL_569098, EPI_ISL_569127, EPI_ISL_569181, EPI_ISL_569234, EPI_ISL_644422, EPI_ISL_644452, EPI_ISL_644470, EPI_ISL_2036729			
see above	MEPHI, Aix Marseille University	MEPHI, Aix Marseille University	Anthony LEVASSEUR
EPI_ISL_631295, EPI_ISL_631303	MVZ DIAMEDIS Diagnostische Medizin Sennestadt GmbH	Bielefeld University	Alexander Sczyrba; Christiane Scherer; David Brandt; Jörn Kalinowski; Levin-Joe Klages; Marina Simunovic; Markus Haak; Svenja Vinke; Tobias Busche
EPI_ISL_1141660	MVZ Labor Dr. Limbach & Kollegen GbR	Robert Koch Institute	
EPI_ISL_860336, EPI_ISL_860355, EPI_ISL_860482, EPI_ISL_860512, EPI_ISL_2869769, EPI_ISL_2869783, EPI_ISL_2869787, EPI_ISL_2869806, EPI_ISL_2869824, EPI_ISL_2869865, EPI_ISL_2869873, EPI_ISL_2869909, EPI_ISL_2869911, EPI_ISL_2869953, EPI_ISL_2870014, EPI_ISL_2870082, EPI_ISL_2870085, EPI_ISL_2870121, EPI_ISL_2870134, EPI_ISL_2870169, EPI_ISL_2870177, EPI_ISL_2870184, EPI_ISL_2870192, EPI_ISL_2870344, EPI_ISL_2870441, EPI_ISL_2870489, EPI_ISL_2870929, EPI_ISL_2870946, EPI_ISL_2871001, EPI_ISL_2871011, EPI_ISL_2871074, EPI_ISL_2871289, EPI_ISL_2871399, EPI_ISL_2871435, EPI_ISL_2871615, EPI_ISL_2871659, EPI_ISL_2872003, EPI_ISL_2872008, EPI_ISL_2872014, EPI_ISL_2872015, EPI_ISL_2872055, EPI_ISL_2872095, EPI_ISL_2872236, EPI_ISL_2872237, EPI_ISL_2872248, EPI_ISL_2872259, EPI_ISL_2872260, EPI_ISL_2872265, EPI_ISL_2872269, EPI_ISL_2872279, EPI_ISL_2872298, EPI_ISL_2872303, EPI_ISL_2872309, EPI_ISL_2872321, EPI_ISL_2872326, EPI_ISL_2872335, EPI_ISL_2872355, EPI_ISL_2872356, EPI_ISL_2872375, EPI_ISL_2872378, EPI_ISL_2872395, EPI_ISL_2872398, EPI_ISL_2872400, EPI_ISL_2872407, EPI_ISL_2872408			
see above	MVZ Labor Krone GbR	Center of Medical Microbiology, Virology, and Hospital Hygiene, University of Duesseldorf	Alexander Dilthey; Andreas Walker; André Heimbach; Bärbel Lippke; Carsten Tiemann; Dennis Deschka; Janine Silvery; Julia Fazaal; Jörg Timm; Kerstin Ludwig; Klaus Pfeffer; Malte Kohns Vasconcelos; Per Hoffmann; Tobias Wienemann; Torsten Houwaart
EPI_ISL_1217731	MVZ Labor Krone GbR	Robert Koch Institute	
EPI_ISL_640220, EPI_ISL_640225, EPI_ISL_640227, EPI_ISL_640231, EPI_ISL_640235, EPI_ISL_640236, EPI_ISL_640245, EPI_ISL_640247, EPI_ISL_640251, EPI_ISL_640260, EPI_ISL_640261, EPI_ISL_640262, EPI_ISL_640265, EPI_ISL_640267, EPI_ISL_640271			
see above	MVZ Laborärzte Singen	MVZ Laborärzte Singen	Folker Wenzel; Frithjof Blessing; Jonas Schmidt; Sandro Berghaus
EPI_ISL_420898, EPI_ISL_420902, EPI_ISL_420904, EPI_ISL_420909	Max von Pettenkofer Institute, Virology, National Reference Center for Retroviruses, LMU Munich	Laboratory for Functional Genome Analysis, Dept. Genomics, Gene Center of the LMU Munich	Alexander Graf; Ashok Varadharajan; Helmut Blum; Max Muenchhoff; Oliver Keppler; Stefan Krebs
EPI_ISL_1751109, EPI_ISL_1751112, EPI_ISL_1751364, EPI_ISL_1751386, EPI_ISL_1751473, EPI_ISL_1751483, EPI_ISL_1751560, EPI_ISL_1751604, EPI_ISL_1751612, EPI_ISL_1751614, EPI_ISL_1751688, EPI_ISL_1751718, EPI_ISL_1751727, EPI_ISL_1751775, EPI_ISL_1751994, EPI_ISL_1752020, EPI_ISL_1752023, EPI_ISL_1752134, EPI_ISL_1752258, EPI_ISL_2094537, EPI_ISL_2094687, EPI_ISL_2094751, EPI_ISL_2094788, EPI_ISL_2095042, EPI_ISL_2095043, EPI_ISL_2095191, EPI_ISL_2095192, EPI_ISL_2095194, EPI_ISL_2095195, EPI_ISL_2095196, EPI_ISL_2095197, EPI_ISL_2095199, EPI_ISL_2095200, EPI_ISL_2095201, EPI_ISL_2095202, EPI_ISL_2095203, EPI_ISL_2095204, EPI_ISL_2095205, EPI_ISL_2095206, EPI_ISL_2095207, EPI_ISL_2095209, EPI_ISL_2095212, EPI_ISL_2095213, EPI_ISL_2095214, EPI_ISL_2095215, EPI_ISL_2095216, EPI_ISL_2095217, EPI_ISL_2095219, EPI_ISL_2095220, EPI_ISL_2095221, EPI_ISL_2095222, EPI_ISL_2095224, EPI_ISL_2095225, EPI_ISL_2095226, EPI_ISL_2095227, EPI_ISL_2095228, EPI_ISL_2095233, EPI_ISL_2095237, EPI_ISL_2095239, EPI_ISL_2095240, EPI_ISL_2095241, EPI_ISL_2095243, EPI_ISL_2095244, EPI_ISL_2095245, EPI_ISL_2095246, EPI_ISL_2095247, EPI_ISL_2095248, EPI_ISL_2095250, EPI_ISL_2095251, EPI_ISL_2095252, EPI_ISL_2095255, EPI_ISL_2095302, EPI_ISL_2450309			
see above	Max von Pettenkofer Institute, Virology, National Reference Center for Retroviruses, LMU Munich	Laboratory for Functional Genome Analysis; Dept. Genomics; Gene Center of the LMU Munich	Alexander Graf; Helmut Blum; Max Muenchhoff; Oliver Keppler; Stefan Krebs
EPI_ISL_437204, EPI_ISL_437233, EPI_ISL_437238, EPI_ISL_437240, EPI_ISL_437243, EPI_ISL_437276, EPI_ISL_437279, EPI_ISL_437359, EPI_ISL_451937, EPI_ISL_451938, EPI_ISL_451941, EPI_ISL_451944, EPI_ISL_466888, EPI_ISL_466889, EPI_ISL_466892, EPI_ISL_466901, EPI_ISL_466905, EPI_ISL_466906, EPI_ISL_466908, EPI_ISL_466909, EPI_ISL_466910, EPI_ISL_466912, EPI_ISL_466917, EPI_ISL_466918, EPI_ISL_466920, EPI_ISL_466922, EPI_ISL_466923, EPI_ISL_466924, EPI_ISL_548957, EPI_ISL_852564, EPI_ISL_852569, EPI_ISL_852574, EPI_ISL_852575, EPI_ISL_852576, EPI_ISL_852580, EPI_ISL_852581, EPI_ISL_852582, EPI_ISL_852584, EPI_ISL_852586, EPI_ISL_852588, EPI_ISL_852589, EPI_ISL_852590, EPI_ISL_852591, EPI_ISL_852592, EPI_ISL_852600			
see above	Max von Pettenkofer Institute, Virology, National Reference Center for Retroviruses, LMU München	Laboratory for Functional Genome Analysis, Dept. Genomics, Gene Center of the LMU Munich	Alexander Graf; Helmut Blum; Max Muenchhoff; Oliver Keppler; Stefan Krebs
EPI_ISL_1347202, EPI_ISL_1347400, EPI_ISL_1347433, EPI_ISL_1990651	Medical Laboratories Duesseldorf	Center of Medical Microbiology, Virology, and Hospital Hygiene, University of Duesseldorf	Alexander Dilthey; Andreas Walker; Angelika Helmer; Christian Lange; Daniel Strelow; Jessica Nicolai; Jörg Timm; Klaus Pfeffer; Lisanna Hülse; Malte Kohns Vasconcelos; Maximilian Damagnez; Nadine Lübke; Tobias Wienemann; Torsten Houwaart
EPI_ISL_549081	Medical Microbiology Unit, Department for Laboratory Medicine, Drammen Hospital, Vestre Viken Health Trust,	Norwegian Institute of Public Health, Department of Virology	Hilde Elshaug; Hilde Synnøve Vollan; Kamilla Heddeland Instefjord; Karoline Bragstad; Kathrine Stene-Johansen; Olav Hungnes; Rasmus Riis Kopperud
EPI_ISL_581453, EPI_ISL_581454, EPI_ISL_581457, EPI_ISL_581459, EPI_ISL_581465, EPI_ISL_581483	Medizinische Klinik Innere Medizin I, Universitätsklinikum Tübingen	NGS Competence Center Tübingen, Institut für Medizinische Mikrobiologie und Hygiene, Universitätsklinikum Tübingen	Angel Angelov
EPI_ISL_1280885	Medizinisches Zentrallabor Altenburg GmbH & Co.KG	Robert Koch Institute	
EPI_ISL_1892868	Microbiologia CATLAB	Can Ruti SARS-CoV-2 Sequencing Hub (HUGTiP/IrsiCaixa/IGTP)	Alba Sánchez; Anna Not; Antoni E Bordoy; Bonaventura Clotet; Cristina Casañ; Cristina Esteban; Francesc Catala-Moll; Gemma Clara; Ignacio Blanco; Marc Noguera-Julian; Maria Casadellà; Mariona Parera; Mercedes Guerrero; Montserrat Giménez; Pere-Joan Cardona; Pilar Armengol; Roger Paredes; Verónica Saludes; and Elisa Martró on behalf of the Can Ruti SARS-CoV-2 Sequencing Hub.
EPI_ISL_2140529, EPI_ISL_2281285, EPI_ISL_2281297, EPI_ISL_2281313	Microbiology Department, Laboratori Clínic Metropolitana Nord. Hospital Universitari Germans Trias i Pujol	Can Ruti SARS-CoV-2 Sequencing Hub (HUGTiP/IrsiCaixa/IGTP)	Alba Sánchez; Anna Not; Antoni E Bordoy; Bonaventura Clotet; Cristina Casañ; Cristina Esteban; Francesc Catala-Moll; Gemma Clara; Ignacio Blanco; Marc Noguera-Julian; Maria Casadellà; Mariona Parera; Mercedes Guerrero; Montserrat Giménez; Pere-Joan Cardona; Pilar Armengol; Roger Paredes; Verónica Saludes; and Elisa Martró on behalf of the Can Ruti SARS-CoV-2 Sequencing Hub.
EPI_ISL_1208415, EPI_ISL_1208651, EPI_ISL_1263405, EPI_ISL_2035872	Microbiology Department, Laboratori Clínic Metropolitana Nord. Hospital Universitari Germans Trias i Pujol.	Can Ruti SARS-CoV-2 Sequencing Hub (HUGTiP/IrsiCaixa/IGTP)	Alba Sánchez; Anna Not; Antoni E Bordoy; Bonaventura Clotet; Carol Galvez Maria Casadellà; Cristina Casañ; Cristina Esteban; Francesc Catala-Moll; Gemma Clara; Ignacio Blanco; Irina Pey; Jordi Barretina; Jose Francisco Sánchez; Julia G Prado; Laura Sumoy; Marc Noguera-Julian; Maria Casadellà; Mariona Parera; Mercedes Guerrero; Montserrat Giménez; Pere-Joan Cardona; Pilar Armengol; Roger Paredes; Verónica Saludes; and Elisa Martró on behalf of the Can Ruti SARS-CoV-2 Sequencing Hub.
EPI_ISL_1502200	Microbiology Department, University Hospital Donostia	Microbiology Department, University Hospital Donostia	Cilla G.; Gomez M; Marimon JM; Montes M; Piñeiro L; Sorarrain A
EPI_ISL_2365883	Microbiology Department. Complexo Hospitalario Universitario de Vigo	Microbiology Department. Complexo Hospitalario Universitario de Vigo	Alfaya N; Alonso I; Alvarez M; Cabrera JJ; Carballo R; Cores O; Cortizo S; Martinez L; Mediero G; Perez S; Potel C; Regueiro B; Rey S; Vasallo FJ; del-Campo V
EPI_ISL_1857293	Microbiology and Virology Laboratory, "Policlinico Riuniti, Azienda Ospedaliero Universitaria, Foggia"	Microbiology and Virology Laboratory, "Policlinico Riuniti, Azienda Ospedaliero Universitaria, Foggia"	Annamaria D'Aprile; Daniela Pisanelli; Donatella Cedola; Fabio Arena; Loris Micelli; Maria Pia Patrizio; Maria Rosaria Lipsi; Maurizio Margaglione and Rosella De Nittis.; Settimia Altamura; Tiziana Rollo; Valeria Delli Carri
EPI_ISL_1170007	Ministry of Health Turkey	Ministry of Health Turkey	Fatma Bayrakdar; Gulay Korukluoglu; Suleyman Yalcin; Yasemin Cosgun
EPI_ISL_1858183, EPI_ISL_1860284, EPI_ISL_1862694, EPI_ISL_1863277, EPI_ISL_1868497, EPI_ISL_1870110, EPI_ISL_1871547, EPI_ISL_1872060, EPI_ISL_1873737, EPI_ISL_1875732, EPI_ISL_1876034, EPI_ISL_1876076, EPI_ISL_1878732, EPI_ISL_1879658, EPI_ISL_1880072, EPI_ISL_1880777, EPI_ISL_1880888, EPI_ISL_1881529, EPI_ISL_1881789, EPI_ISL_1884419, EPI_ISL_1887140, EPI_ISL_1888338, EPI_ISL_1889887, EPI_ISL_1890438, EPI_ISL_1890465, EPI_ISL_1890491, EPI_ISL_1891180, EPI_ISL_1891989, EPI_ISL_1893228, EPI_ISL_1893868, EPI_ISL_1894110, EPI_ISL_1895961, EPI_ISL_1896619			
see above	Molekylær Medicinsk Afdeling, Aarhus University Hospital, Aarhus, Denmark	Aalborg University	Danish Covid-19 Genome Consortium
EPI_ISL_2429333	Molekylær Medicinsk Afdeling, Aarhus University Hospital, Aarhus, Denmark	Statens Serum Institut Bioinformatics and Microbial Genomics	Danish Covid-19 Genome Consortium
EPI_ISL_490205, EPI_ISL_490208	München Klinik Schwabing	MGZ Medical Genetics Center	Dieter A. Wolf; Elke Holinski-Feder
EPI_ISL_1623650, EPI_ISL_1623652	NOVABIO DORDOGNE	CNR Virus des Infections Respiratoires - France SUD	Antonin Bal; Bruno Lina; Gregory Destras; Gwendolyne Burfin; Hadrien Regue; Laurence Josset; Martine Valette; Quentin Semanas
EPI_ISL_416036	National Influenza Center - Instituto Adolfo Lutz	Instituto Adolfo Lutz, Interdiciplinary Procedures Center, Strategic Laboratory	Adriana Bugno; Adriano Abbud; Carlos Henrique Camargo; Claudia Regina Gonçalves; Claudio Tavares Sacchi; Daniela Bernardes Borges da Silva; Erica Valessa Ramos Gomes; Fabiana Cristina Pereira dos Santos; Maria do Carmo Sampaio Tavares Timenetsky; Simone Guadagnucci Morillo; Terezinha Maria de Paiva
EPI_ISL_402125	National Institute for Communicable Disease Control and Prevention (ICDC) Chinese Center for Disease Control and Prevention (China CDC)	National Institute for Communicable Disease Control and Prevention (ICDC) Chinese Center for Disease Control and Prevention (China CDC)	Chen; Dai; F.-H.; Hu, Y.; J.-H.; J.-J.; J.-L. and Zhu; Liu, Y.; Pei; Q.-M.; She; Song; T.-Y.; Tao; Tian; Wang; Wang, W.; Wu, F.; Xu, L.; Y.-L.; Y.-M.; Y.-Y.; Y.-Z.; Yu, B.; Z.-G.; Z.-W.; Zhang; Zhao, S.; Zheng
EPI_ISL_408484	National Institute for Viral Disease Control and Prevention, China CDC	National Institute for Viral Disease Control & Prevention, CCDC	Baoying Huang; Fei Ye; George F. Gao; Guizhen Wu; Huiping Yang; Jianan Xu; Li Zhao; Peihua Niu; Roujian Lu; Wenbo Xu; Wenjie Tan; Wenling Wang; Xiang Zhao
EPI_ISL_2259635	Niedersächsisches Landesgesundheitsamt (NLGA)	Robert Koch Institute	
EPI_ISL_775281, EPI_ISL_775282, EPI_ISL_775283, EPI_ISL_775289, EPI_ISL_775295, EPI_ISL_775296, EPI_ISL_775297, EPI_ISL_775301, EPI_ISL_775302, EPI_ISL_775375, EPI_ISL_2333081, EPI_ISL_2333496, EPI_ISL_2333540			
see above	Nordland Hospital - Bodo, Laboratory Department, Molecular Biology Unit	Norwegian Institute of Public Health, Department of Virology	'Kathrine Stene-Johansen; Atiya R Ali; Debech Nadia; Engebretsen Serina Beate; Garcia Llorente Ignacio; Hilde Elshaug; Hilde Vollan; Jon Bråte; Kamilla Heddeland Instefjord; Karoline Bragstad; Kathrine Stene-Johansen; Line Victoria Moen; Marie Paulsen Madsen; Olav Hungnes; Pedersen Benedikte Nevjen; Rasmus Riis Kopperud
EPI_ISL_573394, EPI_ISL_577035, EPI_ISL_584694, EPI_ISL_584702, EPI_ISL_594989, EPI_ISL_594998, EPI_ISL_595043, EPI_ISL_612527, EPI_ISL_724940, EPI_ISL_725009, EPI_ISL_741447			
see above	Northumbria University / South Tees Hospitals NHS Foundation Trust / North Cumbria Integrated Care NHS Foundation Trust / North Tees and Hartlepool NHS Foundation Trust / Newcastle Hospitals NHS Foundation Trust	COVID-19 Genomics UK (COG-UK) Consortium	Andrew Nelson; Brendan Payne; Clive Graham; Darren L Smith; Debra Padgett; Edward Barton; Emma Swindells; Garren Scott; Gary Black; Gary Eltringham; Giles S Holt; Greg R Young; Jane Greenaway; Jennifer Collins; John Allan; Joshua Loh; Lynn Dover; Matthew Bashton;

			Mohammad A Tariq; Paul Baker; Sarah Essex; Steve Liggett; Wen C Yew; Yusri Taha
EPI_ISL_840382, EPI_ISL_840595, EPI_ISL_841343, EPI_ISL_867338, EPI_ISL_867844, EPI_ISL_951561, EPI_ISL_952084, EPI_ISL_952086, EPI_ISL_952173, EPI_ISL_952302, EPI_ISL_999622, EPI_ISL_999891, EPI_ISL_1052526, EPI_ISL_1052528, EPI_ISL_1052751, EPI_ISL_1053036, EPI_ISL_1053149, EPI_ISL_1053180, EPI_ISL_1053294, EPI_ISL_1106372, EPI_ISL_1106376, EPI_ISL_1107443, EPI_ISL_1179574			
see above	Originating lab: Wales Specialist Virology Centre Sequencing lab: Pathogen Genomics Unit	Public Health Wales Microbiology Cardiff Wales Specialist Virology Centre	Alec Birchley; Alexander Adams; Amy Gaskin; Angela Marchbank; Bree Gatica-Wilcox; Catherine Moore; Jason Coombes; Joanne Watkins; Joel Southgate; Johnathan Evans; Laura Gifford; Lauren Gilbert; Lee Graham; Malorie Perry; Matthew Bull; Nicole Pacchiarini; Sally Corden; Sara Kumziene-Summerhayes; Sara Rey; Sarah Taylor; Simon Cottrell; Sophie Jones; Tom Connor
EPI_ISL_2425845, EPI_ISL_2425847	Oslo University Hospital, Department of Medical Microbiology	Norwegian Institute of Public Health, Department of Virology	Atiya R Ali; Debech Nadia; Engebretsen Serina Beate; Garcia Llorente Ignacio; Hilde Elshaug; Hilde Vollan; Jon Bråte; Kamilla Heddeland Instefjord; Karoline Bragstad; Kathrine Stene-Johansen; Line Victoria Moen; Marie Paulsen Madsen; Olav Hungnes; Pedersen Benedikte Nevjen; Rasmus Riis Kopperud
EPI_ISL_1260869	Ospedale Mater Salutis	Istituto Zooprofilattico Sperimentale delle Venezie	Adelaide Milani; Alessia Schivo; Alice Fusaro; Ambra Pastori; Annalisa Salviato; Antonia Ricci; Calogero Terregino; Edoardo Giussani; Erika Giorgia Quaranta; Isabella Monne; Luca Tassoni
EPI_ISL_833325	Ospedale SS Annunziata Chieti	Istituto Zooprofilattico Sperimentale dell'Abruzzo e Molise "G. Caporale"	Ancora M; Calistri P; Cammà C; Curini V; Di Domenico M; Di Pasquale A; Lorusso A; Mangone I; Marcacci M; Puglia I; Rinaldi A; Savini G
EPI_ISL_549043, EPI_ISL_549044, EPI_ISL_549045, EPI_ISL_549046, EPI_ISL_549047, EPI_ISL_549126, EPI_ISL_549127, EPI_ISL_549130, EPI_ISL_549131, EPI_ISL_549134, EPI_ISL_549136, EPI_ISL_549137, EPI_ISL_549139, EPI_ISL_549140, EPI_ISL_549141, EPI_ISL_549142, EPI_ISL_549145, EPI_ISL_549146, EPI_ISL_549147, EPI_ISL_549148, EPI_ISL_549149, EPI_ISL_549150, EPI_ISL_549151, EPI_ISL_549152, EPI_ISL_549153, EPI_ISL_549155, EPI_ISL_549156, EPI_ISL_549157, EPI_ISL_549158, EPI_ISL_549159, EPI_ISL_549163, EPI_ISL_590922, EPI_ISL_2333080			
see above	Ostfold Hospital Trust - Kalnes, Centre for Laboratory Medicine, Section for gene technology and infection serology	Norwegian Institute of Public Health, Department of Virology	'Kathrine Stene-Johansen; Atiya R Ali; Debech Nadia; Engebretsen Serina Beate; Garcia Llorente Ignacio; Hilde Elshaug; Hilde Synnøve Vollan; Hilde Vollan; Jon Bråte; Kamilla Heddeland Instefjord; Karoline Bragstad; Kathrine Stene-Johansen; Line Victoria Moen; Marie Paulsen Madsen; Olav Hungnes; Pedersen Benedikte Nevjen; Rasmus Riis Kopperud
EPI_ISL_549534, EPI_ISL_559841, EPI_ISL_627257, EPI_ISL_741030, EPI_ISL_1309734	Oxford Viromics, NDM, University of Oxford; Oxford University Hospitals; Basingstoke and North Hampshire Hospital	COVID-19 Genomics UK (COG-UK) Consortium	Alex Mobbs; Amy Trebes; Anita Justice; Catrin Moore; Christophe Fraser; David Bonsall; David Buck; Emma Wise; George Macintyre; Jessica Lynch; John Todd; Mariateresa de Cesare; Matilde Mori; Monique Andersson; Nathan Moore; Nick Cortes; Robert Shaw; Stephen Kidd; Tanya Golubchik; Timothy Peto
EPI_ISL_2367313	PSVJ	Laboratory of genomics and metagenomics	Claire Bertelli; Damien Jacot; Gilbert Greub; Sébastien Aeby; Trestan Pillonel
EPI_ISL_2035944, EPI_ISL_2035948	Pasteur Institute - Laboratory of Clinical Virology	Pasteur Institute - Laboratory of Clinical Virology	Anissa Chouikha; Henda Triki; Kais Ghedira; Mariem Gdoura; Sondos Haddad; Wasfi Fares
EPI_ISL_853923	Pharmgenetix GmbH	Bergthaler laboratory, CeMM Research Center for Molecular Medicine of the Austrian Academy of Sciences	Alexander Lercher; Alexandra Popa; Andreas Bergthaler; Anna Schedl; Benedikt Agerer; Christoph Bock; Jakob-Wendelin Genger; Jan Laine; Lukas Endler; Martin Senekowitsch; Michael Schuster; Thomas Penz
EPI_ISL_2155260	Philippine Red Cross - Port Area	Philippine Genome Center	Alethea R. de Guzman; Anna Ong-Lim; Arianne A. Zamora; Asia Louisa U. Chong; Benedict A. Maralit; Candice Francheska B. Tambaoan; Carlo M. Lapid; Celia Carlos; Devon Ray Pacial; Edsel Maurice Salvaña; El King D. Morado; Eva Maria Cutiongco-de la Paz; Francis A. Tablizo; Irish Coleen A. Asin; Jaime C. Montoya; Jan Michael C. Yap; Jo-Hannah S. Llames; John Q. Wong; Joshua Gregor A. Dizon; Juan Antonio R. Magalang; Karol Sophia Agape R. Padilla; Kenneth M. Kim; Kris P. Punayan; Marc Edsel C. Ayes; Marc Jerrone R. Castro; Maria Rosario Singh-Vergeire and Cynthia P. Saloma; Maria Sofia L. Yangzon; Marissa Alejandria; Razel Nikka M. Hao; Rianna Patricia S. Cruz; Sheila Mae M. Araiza
EPI_ISL_582134	Protzer Lab	Protzer Lab	Alexander Karollus; Andrea Theumer; Dieter Hoffmann; Eva Schulte; Julien Gagneur; Max von Kleist; Oliver Drechsel; Ulrike Protzer
EPI_ISL_1092037, EPI_ISL_1092052, EPI_ISL_1092055, EPI_ISL_1092064, EPI_ISL_1092074, EPI_ISL_1092078, EPI_ISL_1092081, EPI_ISL_1092100, EPI_ISL_1092246, EPI_ISL_1092273, EPI_ISL_1092275, EPI_ISL_1092306, EPI_ISL_1092316, EPI_ISL_1092318			
see above	Protzer Lab	Protzer Lab, Laboratory for Functional Genome Analysis, Dept. Genomics, Gene Center of the LMU Munich	Alexander Graf; Dieter Hoffmann; Elisabeth Esser; Eva C.Schulte; Helmut Blum; Stefan Krebs; Till Bunse; Ulrike Protzer
EPI_ISL_887168, EPI_ISL_887170, EPI_ISL_887175, EPI_ISL_887176, EPI_ISL_887185, EPI_ISL_887187, EPI_ISL_887189, EPI_ISL_887200, EPI_ISL_887202, EPI_ISL_887205, EPI_ISL_887209, EPI_ISL_887217, EPI_ISL_887224, EPI_ISL_887226, EPI_ISL_887231, EPI_ISL_887235, EPI_ISL_887327, EPI_ISL_887347, EPI_ISL_887353, EPI_ISL_887370, EPI_ISL_887372, EPI_ISL_887390, EPI_ISL_887391, EPI_ISL_887398, EPI_ISL_887402, EPI_ISL_887405, EPI_ISL_887408, EPI_ISL_887409			
see above	Protzer Lab	Protzer Lab, Gagneur Lab, Robert Koch Institut	Aleksandar Radonic; Alexander Karollus; Andrea Theumer; Dieter Hoffmann; Eva Schulte; Julien Gagneur; Max von Kleist; Oliver Drechsel; Stephan Fuchs; Ulrike Protzer
EPI_ISL_1373179, EPI_ISL_1373185, EPI_ISL_1373186, EPI_ISL_1373256, EPI_ISL_1373271, EPI_ISL_1373282, EPI_ISL_1373284, EPI_ISL_1373303, EPI_ISL_1373306, EPI_ISL_1373312, EPI_ISL_1373313			
see above	Protzer Lab	Protzer Lab, Institut für Medizinische Mikrobiologie und Hygiene, Gagneur Lab	Angel Angelov; Christina Engesser; Dieter Hoffmann; Elisabeth Esser; Eva C.Schulte; Julien Gagneur; Michael Sonnabend; Nicholas H.Smith; Till Bunse; Ulrike Protzer
EPI_ISL_2379828, EPI_ISL_2380126	Public Health Authority of the Slovak Republic	Public Health Authority of the Slovak Republic	Anna Gičová; Barbora Kotvasová; Elena Tichá; Lucia Ševčíková; Miroslav Böhmer; Pavol Mišenko; Terézia Vrabľová; Tomáš Szemes
EPI_ISL_596164, EPI_ISL_664747	Quadram Institute Bioscience	COVID-19 Genomics UK (COG-UK) Consortium	Alexander J Trotter; Alison E. Mather; Alp Aydin; Ana P. Tedim; Anastasia Kolyva; Andrew Bell; Andrew J. Page; Claire Stuart; Dave J. Baker; Gemma L. Kay; John Wain; Justin O'Grady; Leonardo de Oliveira Martins; Lizzie Meadows; Maria Diaz; Mark Webber; Muhammed Yasir; Nabil-Fareed Alikhan; Ngozi Elumogo; Nicholas M. Thomson; Rachael Stanley; Rachel Gilroy; Reenesh Prakash; Samir Dervisevic; Samuel Bloomfield; Steven Rudder; Thanh Le-Viet
EPI_ISL_594968, EPI_ISL_613321	Queens Medical Centre, Clinical Microbiology Department / DeepSeq Nottingham	COVID-19 Genomics UK (COG-UK) Consortium	Christopher Moore; Fei Sang; Gemma Clark; Hannah Howson-Wells; Johnny Debebe; Jonathan Ball; Joseph Chappell; Manjinder Khakh; Matthew Carlisle; Matthew Loose; Michelle M Lister; Nadine Holmes; Patrick McClure; Theocharis Tsoleridis; Vicki M Fleming; Victoria Wright; Wendy Smith
EPI_ISL_1201590	Regionalne Centrum Krwiodawstwa i Krwiolecznictwa w Białymstoku Pracownia Diagnostyki Molekularnej wirusa SARS-CoV-2	1. National Institute of Public Health - National Institute of Hygiene; 2. Eurofins Genomics Europe Sequencing GmbH	ECDC COVID-19 WGS support team; Eurofins Genomics Europe Sequencing Team; Gierczyński Rafał; Sadkowska-Todys Małgorzata; Wołkowicz Tomasz; Zacharczuk Katarzyna
EPI_ISL_637171, EPI_ISL_686548	Respiratory Virus Unit, Microbiology Services Colindale, Public Health England	COVID-19 Genomics UK (COG-UK) Consortium	PHE Covid Sequencing Team
EPI_ISL_2108816	Robert-Bosch-Krankenhaus	Robert Koch Institute	
EPI_ISL_2521388	Rothen Medizinische Laboratorien AG	Clinical Bacteriology	Adrian Egli; Alfredo Mari; Fanny Wegner; Hans Hirsch; Helena MB Seth-Smith; Ingrid Steffen; Julia Bielicki; Karoline Leuzinger; Manuel Battegay; Tim Roloff
EPI_ISL_1389035	Rothen Medizinische Laboratorien AG	University Hospital Basel, Clinical Bacteriology	Adrian Egli; Alfredo Mari; Hans Hirsch; Helena MB Seth-Smith; Ingrid Steffen; Julia Bielicki; Karoline Leuzinger; Madlen Stange; Manuel Battegay; Tim Roloff
EPI_ISL_2278513, EPI_ISL_2311648	SELAS BIOLAB AVENIR	CHU Purpan - Laboratoire de Virologie - Institut Fédératif de Biologie	Bulach T.; Donnadieu C.; Izopet J.; Latour J.; Milhes M.; Nicot F.; Ranger N.; Salin G.; Tremeaux P.
EPI_ISL_806762	SIESP CHIETI - DRIVE IN ORTONA	Istituto Zooprofilattico Sperimentale dell'Abruzzo e Molise "G. Caporale"	Ancora M; Calistri P; Cammà C; Curini V; Di Domenico M; Di Pasquale A; Lorusso A; Mangone I; Marcacci M; Puglia I; Rinaldi A; Savini G
EPI_ISL_833312	SIESP CHIETI- DRIVE IN CHIETI	Istituto Zooprofilattico Sperimentale dell'Abruzzo e Molise "G. Caporale"	Ancora M; Calistri P; Cammà C; Curini V; Di Domenico M; Di Pasquale A; Lorusso A; Mangone I; Marcacci M; Puglia I; Rinaldi A; Savini G
EPI_ISL_1142071, EPI_ISL_1145693	SYNLAB Jena Oncoscreen	Robert Koch Institute	
EPI_ISL_1152705, EPI_ISL_1355998, EPI_ISL_1434845, EPI_ISL_1568053, EPI_ISL_1847784, EPI_ISL_2112654	SYNLAB MVZ Leinfelden-Echterdingen	Robert Koch Institute	
EPI_ISL_1081786	San Diego County Public Health Laboratory	Andersen lab at Scripps Research	Brett Austin; Jovan Shephard; SEARCH Alliance San Diego with Tracy Basler
EPI_ISL_1393515, EPI_ISL_2132822, EPI_ISL_2132836, EPI_ISL_2132891, EPI_ISL_2510344, EPI_ISL_2510445, EPI_ISL_2510450, EPI_ISL_2510477, EPI_ISL_2510478, EPI_ISL_2510491, EPI_ISL_2510524			
see above	Servicio de Microbiología Clínica (Complejo Hospitalario de Navarra, Pamplona)	Centro de Secuenciación NASERTIC	Ana Miqueleiz; Ana Navascués; Carmen Ezpeleta Baquedano
EPI_ISL_1588941, EPI_ISL_2322395, EPI_ISL_2404889	Servicio de Microbiología Clínica (Complejo Hospitalario de Navarra, Pamplona), Instituto de Investigación Sanitaria de Navarra (IdiSNA)	SeqCOVID-SPAIN consortium/IBV(CSIC)	Ana Miqueleiz and SeqCOVID-SPAIN consortium; Ana Navascués; Carmen Ezpeleta Baquedano
EPI_ISL_1589642, EPI_ISL_2271068, EPI_ISL_2271085, EPI_ISL_2271104, EPI_ISL_2271297	Servicio de Microbiología, Hospital Miguel Servet, Zaragoza	SeqCOVID-SPAIN consortium/IBV(CSIC)	Alexander Tristancho Baró; Ana Milagro; Antonio Rezusta López; Nieves Martínez Cameo and SeqCOVID-SPAIN consortium; Yolanda Gracia Grataloup

EPI_ISL_1013054, EPI_ISL_1013067	Servicio de Microbiología, Laboratori Clínic Metropolitana Nord. Hospital Universitari Germans Trias i Pujol. Institut d'Investigació en Ciències de la Salut Germans Trias i Pujol (IGTP)	IrsiCaixa - Can Ruti CovidSeq	Adrián Antuori; Ana Pérez; Anna Not; Antoni E. Bordoy; Bonaventura Clotet Elisa Martró; Cristina Casañ; Cristina Esteban; Francesc Catala-Moll; Ignacio Blanco; Marc Noguera-Julian; Maria Casadellà; Mariona Parera; Montserrat Giménez; Pilar Armengol; Roger Paredes; Verónica Saludes
EPI_ISL_654526, EPI_ISL_660267, EPI_ISL_660281, EPI_ISL_660301, EPI_ISL_855455, EPI_ISL_855470, EPI_ISL_855478, EPI_ISL_855488, EPI_ISL_855490, EPI_ISL_871933			
see above	Servicio de Microbiología, Laboratori Clínic Metropolitana Nord. Hospital Universitari Germans Trias i Pujol. Institut d'Investigació en Ciències de la Salut Germans Trias i Pujol (IGTP)	SeqCOVID-SPAIN consortium/IBV(CSIC)	Adrián Antuori; Anabel Fernández; Anna Not; Antoni E. Bordoy; Cristina Casañ and SeqCOVID-SPAIN consortium; Elisa Martró; Nona Romaní; Nona Romaní and SeqCOVID-SPAIN consortium; Verónica Saludes
EPI_ISL_1716684, EPI_ISL_1970033, EPI_ISL_1970035, EPI_ISL_1970083, EPI_ISL_1970085, EPI_ISL_1970086, EPI_ISL_1970087, EPI_ISL_2081674, EPI_ISL_2081675			
see above	Servicio de Microbiología. Consorcio Hospital General Universitario de Valencia	SeqCOVID-SPAIN consortium/IBV(CSIC)	Begoña Fuster Escrivá; Carme Salvador García; Concepción Gimeno Cardona and SeqCOVID-SPAIN consortium; María Dolores Ocete; Rafael Medina González
EPI_ISL_1967729, EPI_ISL_1967741, EPI_ISL_1967742, EPI_ISL_1967744, EPI_ISL_1967747	Servicio de Microbiología. Hospital Arnau de Vilanova	SeqCOVID-SPAIN consortium/IBV(CSIC)	Amparo Farga and SeqCOVID-SPAIN consortium; Rocío Falcón; Victoria Domínguez
EPI_ISL_1109658, EPI_ISL_2100113, EPI_ISL_2100132	Servicio de Microbiología. Hospital Clínico Universitario de Valencia	SeqCOVID-SPAIN consortium/IBV(CSIC)	David Navarro Ortega; Eliseo Albert Vicent; Ignacio Torres and SeqCOVID-SPAIN consortium
EPI_ISL_1916225, EPI_ISL_1963495, EPI_ISL_1963496, EPI_ISL_1963853, EPI_ISL_1963881, EPI_ISL_1963894, EPI_ISL_1967760, EPI_ISL_2003991, EPI_ISL_2003992, EPI_ISL_2003995, EPI_ISL_2003998, EPI_ISL_2016370			
see above	Servicio de Microbiología. Hospital General Universitario de Castellón	SeqCOVID-SPAIN consortium/IBV(CSIC)	María Dolores Tirado Balaguer and SeqCOVID-SPAIN consortium; Rosario Moreno Muñoz
EPI_ISL_1963503, EPI_ISL_1963504, EPI_ISL_1963509, EPI_ISL_1963510, EPI_ISL_1963511, EPI_ISL_1963517, EPI_ISL_1963523, EPI_ISL_1963531, EPI_ISL_1963532, EPI_ISL_1963533, EPI_ISL_1963534, EPI_ISL_1963536, EPI_ISL_1963545, EPI_ISL_1963554, EPI_ISL_1963555, EPI_ISL_1963556, EPI_ISL_1967873, EPI_ISL_2100065, EPI_ISL_2135372, EPI_ISL_2135375, EPI_ISL_2135378, EPI_ISL_2135390, EPI_ISL_2135391, EPI_ISL_2135417, EPI_ISL_2439994			
see above	Servicio de Microbiología. Hospital Universitario Doctor Peset	SeqCOVID-SPAIN consortium/IBV(CSIC)	José Miguel Nogueira Coito and SeqCOVID-SPAIN consortium; Juan Alberola Enguídanos; Juan José Camarena Miñana; Rosa González Pellicer
EPI_ISL_2465790, EPI_ISL_2465811	Servicio de Microbiología. Hospital Universitario Donostia. OSI Donostialdea. Área de Enfermedades Infecciosas, Grupo de Infección Respiratoria y Resistencia Antimicrobiana. Instituto de Investigación Sanitaria Biodonostia	SeqCOVID-SPAIN consortium/IBV(CSIC)	Ane Sorrarain; Gustavo Cilla Eguiluz; Jose Maria Marimón and SeqCOVID-SPAIN consortium; Luis Piñeiro Vázquez; Milagrosa Montes Ros
EPI_ISL_1143250	Sonic - Labor Dr. von Froreich GmbH	Robert Koch Institute	
EPI_ISL_1142742	Sonic - MVZ Medizinisches Labor Bremen GmbH	Robert Koch Institute	
EPI_ISL_2156666	Southern Isabela Medical Center Molecular Diagnostic & Research Laboratory (SIMC - MDRL)	Philippine Genome Center	Alethea R. de Guzman; Anna Ong-Lim; Arianne A. Zamora; Asia Louisa U. Chong; Benedict A. Maralit; Candice Francheska B. Tambaoan; Carlo M. Lapid; Celia Carlos; Devon Ray Pacial; Edsel Maurice Salvaña; El King D. Morado; Elcid Aaron R. Pangilinan; Eva Maria Cutiongco-de la Paz; Francis A. Tablizo; Irish Coleen A. Asin; Jaime C. Montoya; Jan Michael C. Yap; Jo-Hannah S. Llames; John Q. Wong; Joshua Gregor A. Dizon; Juan Antonio R. Magalang; Karol Sophia Agape R. Padilla; Kenneth M. Kim; Kris P. Punayan; Marc Edsel C. Ayes; Marc Jerrone R. Castro; Maria Rosario Singh-Vergeire and Cynthia P. Saloma; Maria Sofia L. Yangzon; Marissa Alejandria; Razel Nikka M. Hao; Rianna Patricia S. Cruz; Sheila Mae M. Araiza
EPI_ISL_1147444	Städtisches Klinikum Dresden	Robert Koch Institute	
EPI_ISL_1198341, EPI_ISL_1200033, EPI_ISL_1290543, EPI_ISL_1418731, EPI_ISL_1418733, EPI_ISL_1418748, EPI_ISL_1617456, EPI_ISL_1617540, EPI_ISL_1617547, EPI_ISL_1617906, EPI_ISL_1618382, EPI_ISL_1807545, EPI_ISL_1807595, EPI_ISL_1808178, EPI_ISL_1808575, EPI_ISL_1810683, EPI_ISL_1810695, EPI_ISL_1831791, EPI_ISL_1831826, EPI_ISL_1832060, EPI_ISL_1832062, EPI_ISL_1832150, EPI_ISL_1832390, EPI_ISL_1897831, EPI_ISL_1898194, EPI_ISL_1898223, EPI_ISL_1898276, EPI_ISL_1898339, EPI_ISL_1898593, EPI_ISL_1898716, EPI_ISL_1899503, EPI_ISL_1902492, EPI_ISL_2099074, EPI_ISL_2196373, EPI_ISL_2196395, EPI_ISL_2196396, EPI_ISL_2196420, EPI_ISL_2196425, EPI_ISL_2196436, EPI_ISL_2196446, EPI_ISL_2196455, EPI_ISL_2196462, EPI_ISL_2196466, EPI_ISL_2196484, EPI_ISL_2196485, EPI_ISL_2196487, EPI_ISL_2196488, EPI_ISL_2196489, EPI_ISL_2196490, EPI_ISL_2196491, EPI_ISL_2196493, EPI_ISL_2196494, EPI_ISL_2196499, EPI_ISL_2196512, EPI_ISL_2196515, EPI_ISL_2196518, EPI_ISL_2196603, EPI_ISL_2196644, EPI_ISL_2196656, EPI_ISL_2196678, EPI_ISL_2196680, EPI_ISL_2196683, EPI_ISL_2196723, EPI_ISL_2196733, EPI_ISL_2196744, EPI_ISL_2196747, EPI_ISL_2196749, EPI_ISL_2196755, EPI_ISL_2196761, EPI_ISL_2196764, EPI_ISL_2196769, EPI_ISL_2196782, EPI_ISL_2196791, EPI_ISL_2196792, EPI_ISL_2206208, EPI_ISL_2209928, EPI_ISL_2210680, EPI_ISL_2408740, EPI_ISL_2408755, EPI_ISL_2408811, EPI_ISL_2408907, EPI_ISL_2409069, EPI_ISL_2409135, EPI_ISL_2409367, EPI_ISL_2409412, EPI_ISL_2409491, EPI_ISL_2409600, EPI_ISL_2409735, EPI_ISL_2409754, EPI_ISL_2410837			
see above	Swedish national genomic surveillance program of SARS-CoV-2	The Public Health Agency of Sweden	Alma Brolund; Maria Lind Karlberg; Maximilian Riess; Swedish national genomic surveillance program of SARS-CoV-2
EPI_ISL_2391361	Synlab	Institute of Biomedicine and Translational Medicine, University of Tartu	Aare Abroi; Kristi Huik; Radko Avi; Taavi Päll; Tuuli Reisberg; Ulvi Gerst-Talas
EPI_ISL_1916161, EPI_ISL_1916170, EPI_ISL_1916180	Synlab - Hospital de Manises	SeqCOVID-SPAIN consortium/IBV(CSIC)	Amparo Broseta Tamarit; Carlos Gulin Blanco and SeqCOVID-SPAIN consortium
EPI_ISL_1259052, EPI_ISL_1319325, EPI_ISL_1319384, EPI_ISL_1470092, EPI_ISL_2097010, EPI_ISL_2097016	Synlab Eesti OÜ	1. Laboratory of Communicable Diseases (Estonia); 2. Eurofins Genomics Europe Sequencing GmbH	Liidia Dotsenko et al.
EPI_ISL_2505220	TYKS, Kliininen mikrobiologia	Expert Microbiology, National Institute for Health and Welfare	Carita Savolainen-Kopra; Erika Lindh; Haider al-Hello; Jani Halkilahti; Kirsi Liitsola; Niina Ikonen; Olli Vapalahti; Pekka Ellonen; Phuoc Truong; Päivi Laurila; Ravi Kant; Sari Hannula; Soile Blomqvist; Teemu Smura
EPI_ISL_830237	The National University Hospital of Iceland	deCODE genetics	Agnar Helgason; Alma Moller; Arna B Agustsdottir; Arnaldur Gylfason; Asgeir Sigurdsson; Aslaug Jonasdottir; Berglind Eiriksdottir; Bjarni Thorbjornsson; Brynjar O Jensson; Daniel F Gudbjartsson; Droplaug N Magnusdottir; Elisabet E Gardarsdottir; Emil A Thorarensen; Gardar Sveinbjornsson; Gisli Masson; Gudmundur Georgsson; Gudmundur L Norddahl; Gudrun Sigmundsdottir; Hakon Jonsson; Hannes Eggertsson; Hilma Holm; Ingileif Jonsdottir; Jona Saemundsdottir; Kamilla S Josefsdottir; Kari Stefansson; Karl G Kristinsson; Kjartan R Gudmundsson; Kristin E Sveinsdottir; Louise le Roux; Maney Sveinsdottir; Olafia S Gretarsdottir; Olafur T Magnusson; Pall Melsted; Patrick Sulem; Run Fridriksdottir; Solvi Rognvaldsson; Thora R Gunnarsdottir; Thordur Kristjansson; Thorolfur Gudnason; Unnur Thorsteinsdottir
EPI_ISL_883176, EPI_ISL_883178, EPI_ISL_883187	UK Tübingen, Medical Microbiology	Hannover Medical School, Institute of Virology	Jasper Götting; Lars Steinbrück
EPI_ISL_977182	ULSS 6 Euganea	Istituto Zooprofilattico Sperimentale delle Venezie	Adelaide Milani; Alessia Schivo; Alice Fusaro; Ambra Pastori; Annalisa Salviato; Antonia Ricci; Bianca Zecchin; Calogero Terregino; Erika Giorgia Quaranta; Isabella Monne
EPI_ISL_1260896	ULSS 7 Pedemontana - Distretto 1	Istituto Zooprofilattico Sperimentale delle Venezie	Adelaide Milani; Alessia Schivo; Alice Fusaro; Ambra Pastori; Annalisa Salviato; Antonia Ricci; Calogero Terregino; Edoardo Giussani; Erika Giorgia Quaranta; Isabella Monne; Luca Tassoni
EPI_ISL_2392072	ULSS 8 Berica	Istituto Zooprofilattico Sperimentale delle Venezie	Adelaide Milani; Alessia Schivo; Alice Fusaro; Ambra Pastori; Annalisa Salviato; Antonia Ricci; Calogero Terregino; Edoardo Giussani; Elisa Palumbo; Erika Giorgia Quaranta; Isabella Monne; Luca Tassoni
EPI_ISL_1402565, EPI_ISL_1402573, EPI_ISL_2230585, EPI_ISL_2230586	UMC Groningen, Clinical Virology, Department of Medical Microbiology and Infection Prevention	UMC Groningen, Clinical Virology, Department of Medical Microbiology and Infection Prevention	Alexander Friedrich; Coretta Van Leer-Buter; Erley Lizarazo-Forero; Hubert Niesters; Lilli Gard; Marjolein Knoester; Monika Fliss; Monika Flissikowska; Sigrid Rosema; Xuewei Zhou
EPI_ISL_1715102	UOC laboratorio di analisi Istituto Giannina Gaslini	TIGEM	Antonio Grimaldi Patrizia Annunziata Francesco Panariello Teresa Giuliano Valentina Bouche Chiara Colantuono Lucio Di Filippo Anna Manfredi Marcello Salvi Andrea Ballabio Davide Cacchiarelli
EPI_ISL_735221, EPI_ISL_735222, EPI_ISL_735224, EPI_ISL_735225, EPI_ISL_735226, EPI_ISL_735227, EPI_ISL_735229, EPI_ISL_735230, EPI_ISL_735231, EPI_ISL_735232, EPI_ISL_735233, EPI_ISL_735234, EPI_ISL_735235			
see above	UZ Leuven, National Reference Laboratory for Coronaviruses, Laboratory Medicine, Leuven, Belgium	KU Leuven, Rega Institute, Clinical and Epidemiological Virology	Bert Vanmechelen; Joan Marti-Carerras; Piet Maes; Tony Wawina-Bokalanga
EPI_ISL_1846428	Uniklinikum Carl Gustav Carus an der TU Dresden; Institut fÃ¼r Virologie	Robert Koch Institute	
EPI_ISL_1142093, EPI_ISL_1147561, EPI_ISL_1151780, EPI_ISL_1157246, EPI_ISL_1157275	Uniklinikum Carl Gustav Carus an der TU Dresden; Institut für Virologie	Robert Koch Institute	
EPI_ISL_708063	Unilabs Laboratory Medicine	Norwegian Institute of Public Health, Department of Virology	Hilde Elshaug; Hilde Vollan; Kamilla Heddeland Instefjord; Karoline Bragstad; Kathrine Stene-Johansen; Marie Paulsen Madsen; Olav Hungnes; Rasmus Riis Kopperud
EPI_ISL_763019	Unit 17: Influenza & Other Respiratory Viruses, German National Influenza Center	Project group Epidemiology of Highly Pathogenic Microorganisms, Robert Koch-Institute	Andreas Sachse; Ariane Düx; Djin-Ye Oh; Fabian Leendertz; Grit Schubert; Marianne Wedde; Ralf Dürrwald; Sébastien Calvignac-Spencer; Thorsten Wolff
EPI_ISL_1939063, EPI_ISL_2465747	Universidad de León	SeqCOVID-SPAIN consortium/IBV(CSIC)	Ana Carvajal; Antonio J. Molina and SeqCOVID-SPAIN consortium; Héctor Argüello; Juan M. Fregeneda; Tania Fernández-Villa; Vicente Martín
EPI_ISL_2123892, EPI_ISL_2123896, EPI_ISL_2123904, EPI_ISL_2123917,	Universitatsklinikum Koln; Institut fur Virologie	Robert Koch Institute	

EPI_ISL_2123921, EPI_ISL_2123956			
EPI_ISL_741275, EPI_ISL_839306, EPI_ISL_839308, EPI_ISL_866194, EPI_ISL_866195, EPI_ISL_866256	University College London, Great Ormond Street Hospital for Children NHS Foundation Trust, Imperial College Healthcare NHS Trust	COVID-19 Genomics UK (COG-UK) Consortium	Alison Holmes; Charlotte Williams; Helena Tutill; Jacqueline Findlay; James Price; Judith Breuer; Julianne Brown; Kathryn Harris; Leysa Forrest; Mark Kristiansen; Paola Niola; Paola Resende Silva; Patricia Dyal; Paul Randell; Rachel Williams; Samuel Weeks; Sergi Castellano; Sunando Roy; Tony Brooks; Yasmin Panchbhaya
EPI_ISL_830732, EPI_ISL_830733, EPI_ISL_830734, EPI_ISL_830735, EPI_ISL_930878, EPI_ISL_930912, EPI_ISL_930937, EPI_ISL_930964, EPI_ISL_930979, EPI_ISL_931108, EPI_ISL_931162, EPI_ISL_931180, EPI_ISL_931186, EPI_ISL_931205, EPI_ISL_931234, EPI_ISL_931301, EPI_ISL_931399, EPI_ISL_1388326, EPI_ISL_1388699, EPI_ISL_1388751, EPI_ISL_1388755, EPI_ISL_1388941, EPI_ISL_1389028			
see above	University Hospital Basel, Clinical Virology	University Hospital Basel, Clinical Bacteriology	Adrian Egli; Alfredo Mari; Hans Hirsch; Helena MB Seth-Smith; Julia Bielicki; Karoline Leuzinger; Madlen Stange; Manuel Battegay; Tim Roloff
EPI_ISL_2333492, EPI_ISL_2491561	University Hospital of Northern Norway, Department for Microbiology and Infectious Disease Control	Norwegian Institute of Public Health, Department of Virology	'Kathrine Stene-Johansen; Atiya R Ali; Debech Nadia; Engebretsen Serina Beate; Garcia Llorente Ignacio; Hilde Elshaug; Hilde Vollan; Jon Bråte; Kamilla Heddeland Instefjord; Karoline Bragstad; Kathrine Stene-Johansen; Line Victoria Moen; Marie Paulsen Madsen; Olav Hungnes; Pedersen Benedikte Nevjen; Rasmus Riis Kopperud
EPI_ISL_953647	University Hospitals of Geneva, Laboratory of Virology	HUG, Laboratory of Virology and the Health2030 Genome Center	Ana Rita Goncalves; Deborah Penet; Emmanouil Dermitzakis; Henri Pegeot; Ioannis Xenarios; Keith Harshman; Laurent Kaiser; Lorenzo Cerutti; Melyssa Elies; Samuel Cordey
EPI_ISL_960057	University Medical Center Hamburg Eppendorf	Heinrich Pette Institute, Leibniz Institute for Experimental Virology	Adam Grundhoff; Alexis Robitaille; Johannes Knobloch; Martin Aepfelbacher; Nicole Fischer; Thomas Günther
EPI_ISL_577230, EPI_ISL_585561, EPI_ISL_612389, EPI_ISL_612400, EPI_ISL_627327, EPI_ISL_627328, EPI_ISL_997321			
see above	University of Exeter	COVID-19 Genomics UK (COG-UK) Consortium	Aaron Jeffries; Audrey Farbos; Ben Temperton; Jane Masoli; Joanna Warwick-Dugdale; Michelle Michelsen; Robyn Manley; Stephen Michell
EPI_ISL_930627, EPI_ISL_930628, EPI_ISL_1121214, EPI_ISL_1165864, EPI_ISL_1165868, EPI_ISL_1165871, EPI_ISL_1165873, EPI_ISL_1165875			
see above	University of Liège COVID-19 testing center	GIGA Medical Genomics	Bouchra Boujemla; Cécile Meex; Emmanuel André; Fabrice Bureau; Keith Durkin; Laurent Gillet; Marc Van Ranst; Maria Artesi; Marie-Pierre Hayette; Nathalie Renotte; Sébastien Bontems; Vincent Bours; Wouter Coppieters
EPI_ISL_1336879	Università degli Studi di Perugia	Istituto Zooprofilattico Sperimentale dell'Abruzzo e Molise "G. Caporale"	Ancora M; Calistri P; Camilloni B; Cammà C; Caporale M; Curini V; Di Domenico M; Di Pasquale A; Lorusso A; Mangone I; Marcacci M; Mencacci A; Puglia I; Rinaldi A; Savini G; Scialabba S
EPI_ISL_1154869	Universitätsklinikum Frankfurt - Institut für Medizinische Virologie	Robert Koch Institute	
EPI_ISL_1286470	Universitätsklinikum Heidelberg	Robert Koch Institute	
EPI_ISL_2315927, EPI_ISL_2315935, EPI_ISL_2315962, EPI_ISL_2315972, EPI_ISL_2315977, EPI_ISL_2315979, EPI_ISL_2316004			
see above	Universitätsklinikum Köln; Institut für Virologie	Robert Koch Institute	
EPI_ISL_1146858, EPI_ISL_1155977, EPI_ISL_1217906	Universitätsklinikum Leipzig - Institut für Medizinische Mikrobiologie und Virologie virologisches Labor	Robert Koch Institute	
EPI_ISL_1355695	Universitätsmedizin Mannheim	Robert Koch Institute	
EPI_ISL_2367269	VIDYMED EPALINGES	Laboratory of genomics and metagenomics	Claire Bertelli; Damien Jacot; Gilbert Greub; Sébastien Aeby; Trestan Pillonel
EPI_ISL_549087, EPI_ISL_635106, EPI_ISL_2333078, EPI_ISL_2333493, EPI_ISL_2333497, EPI_ISL_2425846, EPI_ISL_2425848, EPI_ISL_2425849, EPI_ISL_2425850, EPI_ISL_2425851, EPI_ISL_2425852, EPI_ISL_2425853			
see above	Vestfold Hospital, Toensberg Department of Microbiology	Norwegian Institute of Public Health, Department of Virology	'Kathrine Stene-Johansen; Atiya R Ali; Debech Nadia; Engebretsen Serina Beate; Garcia Llorente Ignacio; Hilde Elshaug; Hilde Synnøve Vollan; Hilde Vollan; Jon Bråte; Kamilla Heddeland Instefjord; Karoline Bragstad; Kathrine Stene-Johansen; Line Victoria Moen; Marie Paulsen Madsen; Olav Hungnes; Pedersen Benedikte Nevjen; Rasmus Riis Kopperud
EPI_ISL_1547518	Vestfold Hospital, Toensberg Department of Microbiology	Norwegian Institute of Public Health, Department of Virology	Atiya R Ali; Debech Nadia; Engebretsen Serina Beate; Garcia Llorente Ignacio; Hilde Elshaug; Hilde Vollan; Jon Bråte; Kamilla Heddeland Instefjord; Karoline Bragstad; Kathrine Stene-Johansen; Marie Paulsen Madsen; Olav Hungnes; Pedersen Benedikte Nevjen; Rasmus Riis Kopperud
EPI_ISL_729153, EPI_ISL_899117, EPI_ISL_899436, EPI_ISL_899682, EPI_ISL_899698, EPI_ISL_899701, EPI_ISL_899735, EPI_ISL_899849, EPI_ISL_899972, EPI_ISL_1002535, EPI_ISL_1002600, EPI_ISL_1002686, EPI_ISL_1002706, EPI_ISL_1002714, EPI_ISL_1002752, EPI_ISL_1002764, EPI_ISL_1002950, EPI_ISL_1003007, EPI_ISL_1003651, EPI_ISL_1003725, EPI_ISL_1004367, EPI_ISL_1004405, EPI_ISL_1004579, EPI_ISL_1004600, EPI_ISL_1004777, EPI_ISL_1059474, EPI_ISL_1059791, EPI_ISL_1120085, EPI_ISL_1129534, EPI_ISL_1361242, EPI_ISL_1408477, EPI_ISL_1408527, EPI_ISL_1408808, EPI_ISL_1496279, EPI_ISL_1496579, EPI_ISL_1598861, EPI_ISL_1750802, EPI_ISL_1750863, EPI_ISL_2375353			
see above	Viollier AG	Department of Biosystems Science and Engineering, ETH Zürich	Andrea Patrignani; Andreia Cabral de Gouvea; Catharine Aquino; Chaoran Chen; Christian Beisel; Christiane Beckmann; Christoph Noppen; David Dreifuss; Deborah Penet; Doris Popovic; Elodie Burcklen; Emmanouil Dermitzakis; Griffin White; Henri Pegeot; Ina Nissen; Ioannis Xenarios; Ivan Topolsky; Jay Tracy; Katharina Jahn; Keith Harshman; Lara Fuhrmann; Laura Neff; Lennart Opitz; Lorenzo Cerutti; Maria Domenica Moccia; Maurice Redondo; Mirjam Feldkamp; Natascha Santacroce; Niko Beerenwinkel; Noemie Santamaria de Souza; Olivier Kobel; Pedro Ferreira; Philipp Jablonski; Ralph Schlapbach; Rebecca Denes; Sarah Nadeau; Simon Grüter; Sophie Seidel; Susana Posada-Céspedes; Tanja Stadler; Timothy Sykes; Tobias Schär
EPI_ISL_1388945	Viollier AG	University Hospital Basel, Clinical Bacteriology	Adrian Egli; Alfredo Mari; Christiane Beckmann; Hans Hirsch; Helena MB Seth-Smith; Julia Bielicki; Karoline Leuzinger; Madlen Stange; Manuel Battegay; Tim Roloff
EPI_ISL_651425, EPI_ISL_651428	Virology Department, Royal Infirmary of Edinburgh, NHS Lothian / School of Biological Sciences, University of Edinburgh / Institute of Genetics and Molecular Medicine, University of Edinburgh	COVID-19 Genomics UK (COG-UK) Consortium	Balcaza C; Colquhoun R; Dewar R; Gallagher M; Hill V; Jackson B; McCrone JT; McHugh M; O'Toole Á; Rambaut A; Rooke S; Scher E; Templeton K; Williams TC; Yu X
EPI_ISL_2450756	Virology Laboratory, Department of Biomedical Sciences and Public Health, University Politecnica delle Marche	Virology and Legal Medicine Laboratories, Department of Biomedical Sciences and Public Health, University Politecnica delle Marche	Adriano Tagliabracci; Chiara Turchi; Federica Alessandrini; Filomena Melchionda; Patrizia Bagnarelli; Sara Caucci; Stefano Menzo; Valerio Onofri
EPI_ISL_1855088, EPI_ISL_1855095, EPI_ISL_1855100, EPI_ISL_2036780, EPI_ISL_2036787, EPI_ISL_2036789, EPI_ISL_2036794, EPI_ISL_2036796, EPI_ISL_2036797, EPI_ISL_2036808, EPI_ISL_2036868, EPI_ISL_2036911, EPI_ISL_2362149, EPI_ISL_2362151, EPI_ISL_2362156, EPI_ISL_2362158, EPI_ISL_2362168, EPI_ISL_2362169, EPI_ISL_2362173, EPI_ISL_2362174, EPI_ISL_2362175, EPI_ISL_2362212			
see above	Virology Laboratory, Scientific Department, Army Medical Center	Virology Laboratory, Scientific Department, Army Medical Center	Anella Monte; Anna Anselmo; Antonella Fortunato; Filippo Molinari; Florigio Lista; Francesco Giordani; Gaetano Alfano; Giancarlo Petralito; Giandomenico Cerreto; Giulia Campoli; Lucia Nicosia; Margherita De Santis; Maria Anna Spinelli; Marzia Cavalli; Riccardo De Sanctis; Rossella Brandi; Silvia Fillo; Vanessa Vera Fain
EPI_ISL_1366938, EPI_ISL_1366939, EPI_ISL_1366979, EPI_ISL_1577876, EPI_ISL_1648028, EPI_ISL_1786491, EPI_ISL_1864365, EPI_ISL_1864371, EPI_ISL_1864398, EPI_ISL_2003713, EPI_ISL_2003734, EPI_ISL_2135786, EPI_ISL_2135802, EPI_ISL_2447951, EPI_ISL_2447967, EPI_ISL_2447977, EPI_ISL_2447985			
see above	Virology, Universitätsklinikum des Saarlandes	Epigenetics, Saarland University	Jörn Walter; Kathrin Kattler; Markus Vogelgesang; Nastasja Seiwert; Sascha Tierling; Sigrun Smola; Stefan Lohse; Thorsten Pfuhl
EPI_ISL_1239709	Vitalabo Bydgoszcz	1. National Institute of Public Health - National Institute of Hygiene; 2. Eurofins Genomics Europe Sequencing GmbH	ECDC COVID-19 WGS support team; Eurofins Genomics Europe Sequencing Team; Gierczyński Rafał; Sadkowska-Todys Małgorzata; Wołkowicz Tomasz; Zacharczuk Katarzyna
EPI_ISL_574145, EPI_ISL_574150, EPI_ISL_585801, EPI_ISL_612132, EPI_ISL_627789, EPI_ISL_639547, EPI_ISL_666230, EPI_ISL_666261, EPI_ISL_680942, EPI_ISL_705442, EPI_ISL_706462, EPI_ISL_706749, EPI_ISL_706783, EPI_ISL_726270, EPI_ISL_726282, EPI_ISL_726285, EPI_ISL_726293, EPI_ISL_726298, EPI_ISL_727025, EPI_ISL_727190, EPI_ISL_742469, EPI_ISL_742636, EPI_ISL_742715, EPI_ISL_742877, EPI_ISL_743142, EPI_ISL_743219, EPI_ISL_764640, EPI_ISL_814595, EPI_ISL_814754, EPI_ISL_814860, EPI_ISL_814896, EPI_ISL_814910, EPI_ISL_814918, EPI_ISL_815071, EPI_ISL_822820, EPI_ISL_822832, EPI_ISL_823331, EPI_ISL_823333, EPI_ISL_823408, EPI_ISL_823784			
see above	Wales Specialist Virology Centre Sequencing lab: Pathogen Genomics Unit	COVID-19 Genomics UK (COG-UK) Consortium	Alec Birchley; Alexander Adams; Amy Gaskin; Angela Marchbank; Bree Gatica-Wilcox; Catherine Moore; Jason Coombes; Joanne Watkins; Joel Southgate; Johnathan Evans; Laura Gifford; Lauren Gilbert; Lee Graham; Malorie Perry; Matthew Bull; Nicole Pacchiarini; Sally Corden; Sara Kumziene-Summerhayes; Sara Rey; Sarah Taylor; Simon Cottrell; Sophie Jones; Tom Connor
EPI_ISL_922579, EPI_ISL_922879, EPI_ISL_1106846, EPI_ISL_1107176, EPI_ISL_1833128, EPI_ISL_1833131, EPI_ISL_1833136, EPI_ISL_1833138, EPI_ISL_1833141, EPI_ISL_1833142, EPI_ISL_1833143, EPI_ISL_1833145, EPI_ISL_1833146, EPI_ISL_1833149, EPI_ISL_1833151, EPI_ISL_1833166			
see above	Wales Specialist Virology Centre Sequencing lab: Pathogen Genomics Unit	Public Health Wales Microbiology Cardiff Wales Specialist Virology Centre	Alec Birchley; Alexander Adams; Amy Gaskin; Angela Marchbank; Bree Gatica-Wilcox; Catherine Moore; Jason Coombes; Joanne Watkins; Joel Southgate; Johnathan Evans; Laura Gifford; Lauren Gilbert; Lee Graham; Malorie Perry; Matthew Bull; Nicole Pacchiarini; Sally Corden; Sara Kumziene-Summerhayes; Sara Rey; Sarah Taylor; Simon Cottrell; Sophie Jones; Tom Connor
EPI_ISL_627361, EPI_ISL_627362, EPI_ISL_627364, EPI_ISL_627367, EPI_ISL_651767	West of Scotland Specialist Virology Centre, NHSGGC / MRC-University of Glasgow Centre for Virus Research	COVID-19 Genomics UK (COG-UK) Consortium	Alasdair MacLean; Alice Broos; Ana da Silva Filipe; Antonia Ho; Daniel Mair; David L Robertson; Elihu Aranday-Cortes; Emma Thomson; Guy Mollett; Igor Starinskij; James Shepherd; Jenna Nichols; Joseph Hughes; Kathy Li; Kathy Smollett; Kyriaki Nomikou; Lily Tong; Marc Niebel; Matthew Holden; Natasha Jesudason; Natasha Johnson; Patawee Asamaphan; Rachel Blacow; Rajiv Shah; Richard Orton; Rory Gunson; Sarah McDonald; Sharif Shaaban; Sreenu Vattipally; Stephen Carmichael
EPI_ISL_539580, EPI_ISL_539584, EPI_ISL_539585, EPI_ISL_539588, EPI_ISL_539591, EPI_ISL_539598, EPI_ISL_539600, EPI_ISL_539602, EPI_ISL_539606, EPI_ISL_539612, EPI_ISL_539613, EPI_ISL_539614, EPI_ISL_631304, EPI_ISL_631315, EPI_ISL_631344, EPI_ISL_631355, EPI_ISL_631356, EPI_ISL_631368, EPI_ISL_666630, EPI_ISL_666641, EPI_ISL_666650, EPI_ISL_666651, EPI_ISL_718035, EPI_ISL_718063, EPI_ISL_718077, EPI_ISL_718078, EPI_ISL_718101, EPI_ISL_770700, EPI_ISL_770707, EPI_ISL_770708, EPI_ISL_770738, EPI_ISL_770740, EPI_ISL_770741, EPI_ISL_770742, EPI_ISL_770769, EPI_ISL_770771, EPI_ISL_1347493, EPI_ISL_1347528, EPI_ISL_1347574, EPI_ISL_1347739, EPI_ISL_1988342, EPI_ISL_2380677			
see above	ZOTZ KLIMAS MVZ Düsseldorf-Centrum GbR ÜBAG für Labormedizin, Genetik, Zytologie, Pathologie	Center of Medical Microbiology, Virology, and Hospital Hygiene, University of Duesseldorf	Alexander Dilthey; Andreas Walker; Ashley-Jane Duplessis; Daniel Strelow; Jessica Nicolai; Jörg Timm; Katrin Hoffmann; Klaus Pfeffer; Lisanna Hülse; Malte Kohns Vasconcelos; Marek Korencak; Maximilian Damagnez; Nadine Lübke; Patrick Finzer; Rainer Zotz; Tobias Wienemann; Torsten Houwaart
EPI_ISL_1281384, EPI_ISL_1351394	amedes Göttingen	Robert Koch Institute	
EPI_ISL_1850570	amedes MVZ DIAMEDIS Sennestadt	Robert Koch Institute	
EPI_ISL_1146243	amedes MVZ Jena	Robert Koch Institute	

EPI_ISL_826933, EPI_ISL_827227, EPI_ISL_827401, EPI_ISL_827523, EPI_ISL_827796	deCODE genetics	deCODE genetics	Agnar Helgason; Alma Moller; Arna B Agustsdottir; Arnaldur Gylfason; Asgeir Sigurdsson; Aslaug Jonasdottir; Berglind Eiriksdottir; Bjarni Thorbjornsson; Brynjar O Jensson; Daniel F Gudbjartsson; Droplaug N Magnusdottir; Elisabet E Gardarsdottir; Emil A Thorarensen; Gardar Sveinbjornsson; Gisli Masson; Gudmundur Georgsson; Gudmundur L Norddahl; Gudrun Sigmundsdottir; Hakon Jonsson; Hannes Eggertsson; Hilma Holm; Ingileif Jonsdottir; Jona Saemundsdottir; Kamilla S Josefsdottir; Kari Stefansson; Karl G Kristinsson; Kjartan R Gudmundsson; Kristin E Sveinsdottir; Louise le Roux; Maney Sveinsdottir; Olafia S Gretarsdottir; Olafur T Magnusson; Pall Melsted; Patrick Sulem; Run Fridriksdottir; Solvi Rognvaldsson; Thora R Gunnarsdottir; Thordur Kristjansson; Thorolfur Gudnason; Unnur Thorsteinsdottir
EPI_ISL_1007943, EPI_ISL_1007976, EPI_ISL_1007979, EPI_ISL_1007982, EPI_ISL_1008715, EPI_ISL_1008721, EPI_ISL_1008722, EPI_ISL_1008724, EPI_ISL_1008747			
see above	genXone SA, Molecular Diagnostics Laboratory / NZOZ	genXone SA, Research & Development Laboratory	Aleksandra Gidlewicz; Anna Brylak-Błaszków; Grzegorz Nowicki; Jakub Grabowski; Karol Szeszko; Maciej Sykulski; Michał Kaszuba; Natalia Drwęska-Matelska; Łukasz Krych
EPI_ISL_1351559	nordlab - Partnerschaftspraxis fu?r Laboratoriumsmedizin	Robert Koch Institute	
EPI_ISL_2445641	unknown	Instituto Nacional de Saude (INSA)	Borges et al
