# Supplementary material for: Investigation of a Limited but Explosive COVID-19 Outbreak in a German Secondary School
Source: Viruses. 2022 Jan 4;14(1):87. doi: 10.3390/v14010087 (PMC8780098; doi:10.3390/v14010087)
Supplement: Supplementary file 1 [file viruses-14-00087-s001.zip › supplement_figure_1.pdf]

view of window front

room 1

view of window front

room 2 and 3, class 8.2 and 8.3
